# In vivo clustering of *oskar* mRNA is driven by RNA concentration, RNA binding proteins, and an RNA palindrome

**DOI:** 10.1091/mbc.E26-04-0176

**Published:** 2026-08-12

**Authors:** Ziqing Ye, Siran Tian, Ayse Ecer, Tatjana Trcek

**Affiliations:** ^a^Department of Biology, Johns Hopkins University, 3400 N. Charles Street, Baltimore, MD 21218; Emory University

## Abstract

mRNA organization into clusters is observed in many cellular contexts, yet the features that govern this process in vivo remain poorly understood. Using super-resolution microscopy, single-mRNA imaging, and genetic perturbations, we investigated how mRNA concentration, the double-stranded RNA-binding protein Staufen, and intermolecular base-pairing driven by an RNA palindrome influence clustering of *oskar* mRNA in *Drosophila* embryos. We find that these factors collectively optimize *oskar* clustering by promoting its dimerization and subsequent oligomerization. Both processes depend on all three factors, although oligomerization is much more sensitive to their perturbation, indicating that the driving force for *oskar* oligomerization is partially distinct from that governing dimerization. Moreover, *oskar* palindrome is a potent driver of heterotypic mRNA clustering, further supporting its in vivo role in mediating intermolecular base pairing. Finally, computational analyses identified a subset of candidate mRNAs in the early embryo that are predicted to harbor *oskar*-like palindromes. Among these, *eIF3a* mRNA emerged as a potential candidate whose clustering may likewise be driven by intermolecular base pairing. These preliminary observations raise the possibility that mRNA clustering driven by palindrome-mediated intermolecular base pairing may be more widespread than previously appreciated and may represent an important mechanism for controlling mRNA spatial organization during *Drosophila* development.

## Abstract (executive summary)

- Organization of mRNAs into clusters is widespread in cells, yet the molecular mechanisms that drive this process in vivo remain poorly understood.
- We show that clustering of *oskar* mRNA in *Drosophila* is governed by the combined actions of RNA concentration, the RNA-binding protein Staufen, and an RNA palindrome that promotes intermolecular base pairing. Although these factors drive efficient *oskar* clustering, they contribute distinctly to different stages of cluster assembly.
- We identified candidate mRNAs that may contain similar palindromes, suggesting that palindrome-mediated intermolecular base pairing may represent a broader mechanism for oligomerization and spatial organization of mRNAs in *Drosophila*.

## INTRODUCTION

Spatial organization of messenger RNAs (mRNAs) is a widespread mechanism for controlling the spatial and temporal production of proteins within cells and is particularly impactful during organismal development, where localized mRNAs influence cell fate, morphology, and body plan formation across metazoans (Holt and Bullock, 2009).

Beyond being targeted to defined cellular structures such as the endoplasmic reticulum or RNA granules, mRNAs can also organize with one another to form mRNA clusters (Little *et al.*, 2015; Trcek *et al.*, 2015; Aw *et al.*, 2016; Lu *et al.*, 2016; Pichon *et al.*, 2016; Sharma *et al.*, 2016; Cai *et al.*, 2020; Chouaib *et al.*, 2020; Gabryelska *et al.*, 2022; Cardona *et al.*, 2023; Klobucar *et al.*, 2026). These clusters, which may contain only a few transcripts, arise through intermolecular interactions involving both RNA-binding proteins (RBPs) as well as the RNAs themselves (Tian *et al.*, 2020). In this context, RNAs can serve as scaffolds that recruit RBPs to drive mRNA clustering, or they may directly promote clustering via RNA:RNA interactions (Wagner *et al.*, 2001; Jambor *et al.*, 2011; Jain and Vale, 2017; Van Treeck and Parker, 2018; Van Treeck *et al.*, 2018; Elguindy and Mendell, 2021).

Intermolecular base pairing between complementary sequences (CSs) represents a prominent mechanism of RNA-driven clustering (Eisenberg and Felsenfeld, 1967; Clever *et al.*, 1996; Ferrandon *et al.*, 1997; Jambor *et al.*, 2011; Jain and Vale, 2017; Langdon *et al.*, 2018; Van Treeck *et al.*, 2018; Tauber *et al.*, 2020; Tian *et al.*, 2025). This mechanism is well characterized in RNA viruses where genome dimerization and higher-order assembly depend on defined CS elements. For example, viruses like Influenza A, Human immunodeficiency virus-1 (HIV-1), Murine leukemia virus (MLV), and the Flock House virus (FHV) rely on intermolecular base pairing to organize and package their RNA genomes (Berkowitz *et al.*, 1996; De Tapia *et al.*, 1998; Krishna and Schneemann, 1999; Kim and Tinoco, 2000; Paillart *et al.*, 2004; Johnson and Telesnitsky, 2010; Dadonaite *et al.*, 2019; Le Sage *et al.*, 2020; Zhou and Routh, 2024), and disruption of these interactions blocks virion assembly (Berkowitz *et al.*, 1996; De Tapia *et al.*, 1998; Paillart *et al.*, 2004; Johnson and Telesnitsky, 2010; Zhou and Routh, 2024). Dimerization-competent CSs are typically structurally exposed, which facilitates recognition among mRNAs and positions them for stable and persistent interactions (Clever *et al.*, 1996; Paillart *et al.*, 2004; Hussein *et al.*, 2010; Johnson and Telesnitsky, 2010; Bou-Nader and Zhang, 2020). They are also often GC-rich, which thermodynamically enhances the stability of base pairing. For example, in HIV-1, a six-nucleotide GC-rich palindrome located at the apex of a stem-loop nucleates RNA dimerization (Marquet *et al.*, 1994; Clever *et al.*, 1996).

**FIGURE 1:**
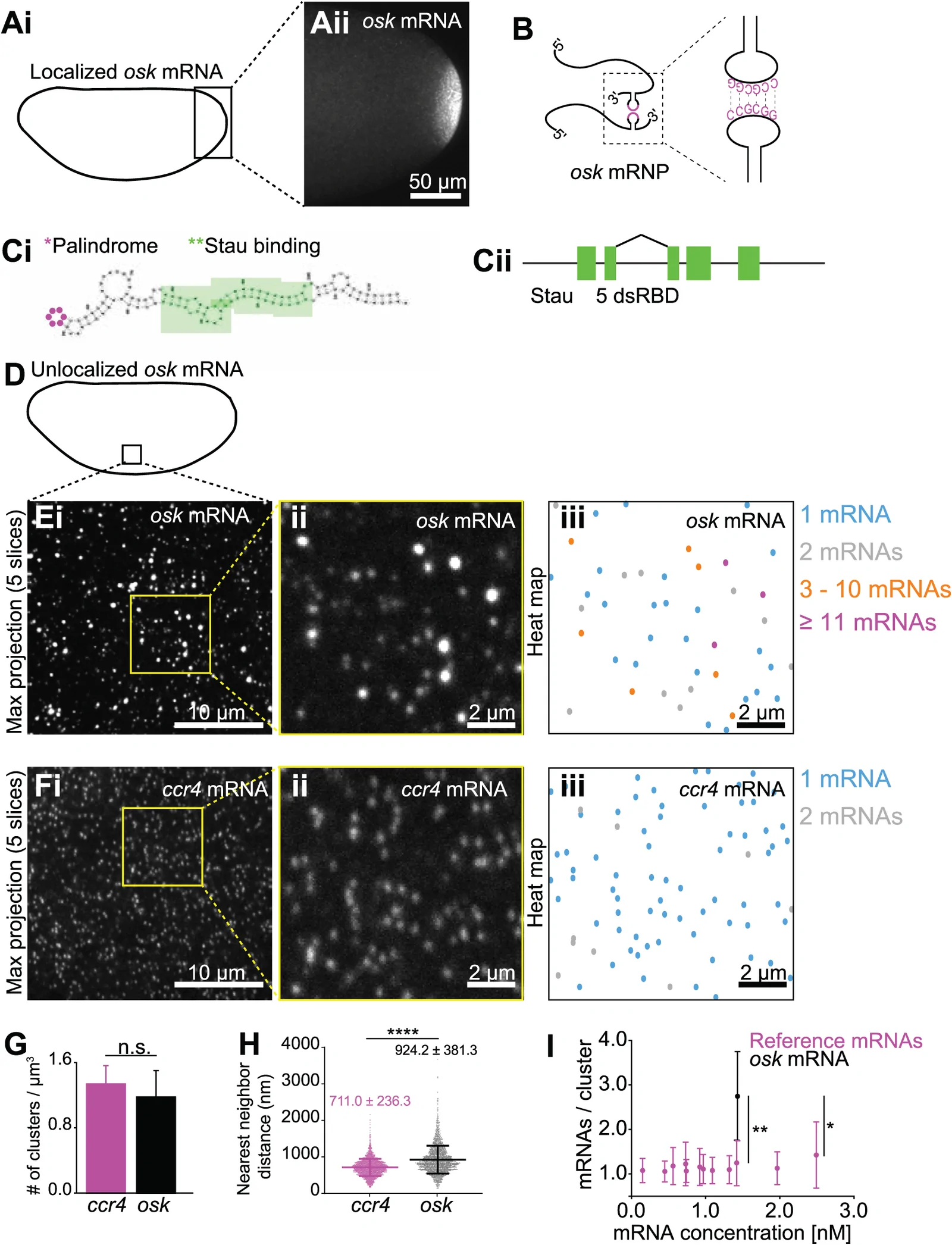
Applying smFISH to examine *osk* mRNA clustering in early embryos. **A, i and ii** Schematic of an embryo depicting the location of localized *osk* mRNA (i) accumulated at the posterior pole stained with smFISH (ii). **B** Schematic illustrating dimerization of *osk* mediated by an RNA palindrome. The palindrome (CCGCGG) is exposed atop a long RNA stem (magenta), which facilitates intermolecular base pairing of *osk* mRNAs and enables its hitchhiking (Jambor *et al.*, 2011; Jambor *et al.*, 2014). **C, i and ii.** A diagram showing the position of five dsRBDs (green boxes) of the *Drosophila* Stau (Micklem *et al.*, 2000) (i) and the position of Stau binding to the *osk* stem-loop structure (green boxes). The nucleotides of the *osk* palindrome are labeled in magenta(Jambor *et al.*, 2014) (ii). The stem-loop structure comprised nucleotides 714–827, which is the conserved region within the dimerization-competent portion of the *osk* 3′UTR defined previously (Jambor *et al.*, 2014). **D** Schematic of an embryo depicting an approximate location of a 3D region of interest (ROI) used to analyze clustering of unlocalized *osk* mRNA in Ei–iii. **Ei–iii.** Maximal projection of five consecutive optical sections of an ROI taken from the somatic region of the embryo (Ai), spaced 150 nm apart (i). Yellow ROI: zoomed-in image shown in (ii) with an accompanying heat map image showing the distribution of *osk* mRNA clusters of different sizes (iii). **F, i–iii** Maximal projection of five consecutive optical sections spaced 150 nm apart taken from an embryo stained with smFISH probes hybridizing to *ccr4* mRNA (i). Yellow ROI: zoomed-in image (ii) with an accompanying heat map image (iii). **G** Number of fluorescent *ccr4* or *osk* clusters per µm^3^ volume (density). Mean ± STDEV of five and seven 3D ROIs is plotted. Statistical significance: two-tailed *t* test. n.s.: not significant. **H** Shortest nm distance between neighboring *ccr4* or *osk* clusters. Mean ± STDEV of 2999 and 2074 *cc4* and *osk* clusters, respectively, were analyzed. Statistical significance: two-tailed *t* test. ****: *P* < 0.001. **I** Average size of *osk* mRNA clusters (black circle and error bar) and of 12 reference mRNAs (*aret, ccr4*, *CG18446*, *eIF4G2*, *gcl*, *orb, Pi3K*, *p53*, *pum*, *shutdown*, *sra* and *tao1;* magenta circles and error bars) plotted against their respective somatic concentrations. The data for the reference mRNAs were determined previously (Trcek *et al.*, 2020). All differences in cluster sizes between *osk* (black circles) and reference mRNAs (magenta circles) are statistically significant, with the *P* values ranging from 0.01 (*) to 0.004 (**; two-tailed *t* test).

Intermolecular base pairing also operates in eukaryotic mRNAs. In the filamentous fungus *Ashbya gossypii*, base pairing between *BNI1* and *SPA2* mRNAs, both essential for cell cycle progression and nuclear division, drives their organization within cells (Langdon *et al.*, 2018). This organization is important for establishing compositional specificity of biomolecular condensates associated with polarized growth and nuclear divisions in the fungus (Langdon *et al.*, 2018). Similarly, in *Drosophila*, complementary sequence interactions between the *oskar* (*osk*; Figure 1A, i and ii) and *bicoid* (*bcd*), two mRNAs that are critical for early embryogenesis, promote their respective enrichment at distinct embryonic locations (Ferrandon *et al.*, 1997; Jambor *et al.*, 2011; Jambor *et al.*, 2014).

In addition to RNA sequence features, RNA concentration and RBPs can also enhance mRNA clustering driven by CSs. For instance, dimerization of the MLV RNA is thought to occur during transcription, where local viral RNA concentration is high (Maurel and Mougel, 2010). Likewise, HIV-1 RNA dimerization frequently takes place upon RNA localization to the plasma membrane and requires the viral Gag protein (Weiss *et al.*, 1984; Muriaux *et al.*, 1996), while clustering of *BNI1*, *SPA2*, *osk* and *bcd* mRNA similarly depends on their respective RBPs (Ferrandon *et al.*, 1997; Chekulaeva *et al.*, 2006; Besse *et al.*, 2009; Langdon *et al.*, 2018; Bose *et al.*, 2024). Together, these observations suggest that RNA concentration and RBPs are important for establishing efficient CS-dependent intermolecular base pairing to promote RNA clustering. However, how these factors enhance mRNA clustering in vivo has not been systematically investigated.

Here, we dissect the contribution of mRNA concentration, RBPs, and intermolecular base pairing to the clustering of the *Drosophila osk* mRNA. *osk*’s ability to engage in intermolecular base pairing has been experimentally validated both in vitro and in vivo. Specifically, *osk* contains an exposed palindrome (CCGCGG) positioned at the apex of a stem within its 3′ untranslated region (UTR), which mediates intermolecular base pairing between *osk* mRNAs (Jambor *et al.*, 2011; Jambor *et al.*, 2014; Figure 1B). This palindrome enables one *osk* mRNA to “hitchhike” on another during its transport to the posterior pole (Jambor *et al.*, 2011; Jambor *et al.*, 2014; Figure 1B). Mutations that disrupt the palindrome abolish dimerization of *osk* 3′UTR in vitro and posterior localization of the mRNA in vivo (Jambor *et al.*, 2011). Furthermore, *trans* compensatory mutations revealed that dimerization of *osk* occurs through direct intermolecular base pairing in vivo (Jambor *et al.*, 2011), highlighting the palindrome as a critical *cis*-acting element required for *osk* mRNA localization. At the posterior pole, hundreds of *osk* mRNA copies oligomerize within founder granules (Little *et al.*, 2015; Eichler *et al.*, 2020; Trcek *et al.*, 2020; Eichler *et al.*, 2023), wherein *osk* palindrome remains engaged in intermolecular base pairing (Trcek *et al.*, 2020), acting as an “interaction sticker” (Bose *et al.*, 2024) that supports *osk* oligomerization in vivo.

While distinct RBPs promote *osk* mRNA clustering (Chekulaeva *et al.*, 2006; Besse *et al.*, 2009; Bose *et al.*, 2024), in this study we examined the contribution of the double-stranded RBP (dsRBP) Staufen (Stau) as a model to evaluate the contribution of RBPs to mRNA clustering. We focused on Stau for several key reasons. First, Stau is essential for *osk* mRNA localization to the posterior pole, and in its absence, *osk* fails to localize (Ephrussi *et al.*, 1991; Kim-Ha *et al.*, 1991; Mohr *et al.*, 2021). Since intermolecular base pairing between *osk* mRNA also enhances *osk* localization to the posterior (Jambor *et al.*, 2011), we reasoned that Stau may contribute to dimerization of *osk* as well. Second, Stau is a dsRBP (St Johnston *et al.*, 1992) that specifically recognizes the stem structure that carries the palindrome within the *osk* 3′UTR (Figure 1Ci, green segments; Laver *et al.*, 2013; Mohr *et al.*, 2021). Mutations that disrupt base pairing within this stem abolish Stau binding, while compensatory mutations that restore base pairing also restore Stau binding (Mohr *et al.*, 2021). Moreover, mutations of the first two cytosines in the palindrome disrupt *osk* dimerization in vitro and its localization in vivo (Jambor *et al.*, 2011), but preserve Stau binding (Bose *et al.*, 2024). Therefore, Stau association with the stem that carries the palindrome does not require prior dimerization. This may allow Stau to directly promote initial RNA dimerization steps. Lastly, *Drosophila* Stau contains five RNA-binding domains (Figure 1Cii; Ramos *et al.*, 2000), suggesting that it may function as a molecular bridge, linking RNA monomers and promoting their assembly as well as stabilizing intermolecular base pairing interactions required for oligomerization (Ferrandon *et al.*, 1997).

Here, we demonstrate that RNA concentration, Staufen, and the RNA palindrome together promote *osk* mRNA clustering, with *osk* oligomerization being the most strongly affected by the perturbations to these three factors. Our work suggests that this multi-layered requirement likely increases the stringency of clustering, making it a tightly regulated and context-dependent process in vivo.

## RESULTS

### Applying smFISH to examine *osk* mRNA clustering in early embryos

We combined structured illumination microscopy, a super-resolution approach, with 48 single-molecule fluorescent in situ hybridization (smFISH) probes labeling the *osk* sequence (Figure 1, D and E, i and ii; Supplemental Table S1, *Materials and Methods* section) to examine the effect of *osk* mRNA concentration, dsRBP Stau, and the RNA palindrome on clustering of unlocalized *osk* in early embryos. We examined unlocalized *osk* mRNA, specifically, to detect individual and clustered *osk* mRNAs, which would be more difficult to discern within founder granules at the posterior pole where localized *osk* exhibits enhanced clustering (Figure 1A, i and ii; Eichler *et al.*, 2023).

We quantified the *osk* cluster size, defined as the absolute number of *osk* mRNAs within each diffraction-limited smFISH spot, and *osk* mRNA concentration for each embryo, as we have done previously (Trcek *et al.*, 2015; Trcek *et al.*, 2017; Trcek *et al.*, 2020) (*Materials and Methods*). Fitting the distribution of fluorescent puncta to a double-Gaussian curve (Supplemental Figure S1A, black circles and line) revealed an *osk* population with two mean fluorescence intensities: X_0_ = 154.3 ± 2.9 arbitrary unites (a.u.), and a second mean intensity that was twice the value of X_0_ (X_1_ = 277.2 ± 25.0; *P* < 0.0001, *R^2^*: 0.96; Supplemental Table S1, *Materials and Methods* section).

Importantly, the intensity of the first Gaussian peak, which represented the population of single mRNAs, closely matched the value of 154.9 ± 4.6 a.u. determined in embryos with *osk* mRNA levels reduced *via* RNAi, whose expression was driven by a GAL4/UAS system under a strong maternal *alpha-tubulin* (*matα*) promoter (Curnutte *et al.*, 2023; referred to as “RNAi/*matα*”; *P* < 0.0001, *R^2^*: 0.97; Supplemental Table S1). We have confirmed that the reduction of *osk* expression in these embryos was strong (Supplemental Figure S1B).

Moreover, this value also closely matched the mean fluorescence intensity of 153.0 ± 4.6 a.u. recorded in embryos lacking Stau expression (referred to as “*stau^1^/stau^2^*”; *P* < 0.0001, *R^2^*: 0.95; Supplemental Table S1). We confirmed that the *stau^1^/stau^2^* embryos failed to localize *osk* at the posterior pole (Supplemental Figure S1C) and did not hatch (Supplemental Figure S1D), consistent with previous observations (Ephrussi *et al.*, 1991). Indeed, the average intensity of the second Gaussian was twice the intensity of the average intensity of the first Gaussian, which is consistent with the second Gaussian peak representing the mRNA dimers in the population (Supplemental Table S1). Therefore, our approach robustly and reproducibly detected single *osk* mRNAs across different genetic backgrounds in vivo, and enabled us to quantify the discrete number of *osk* mRNAs in oligomerized clusters.

**FIGURE 2:**
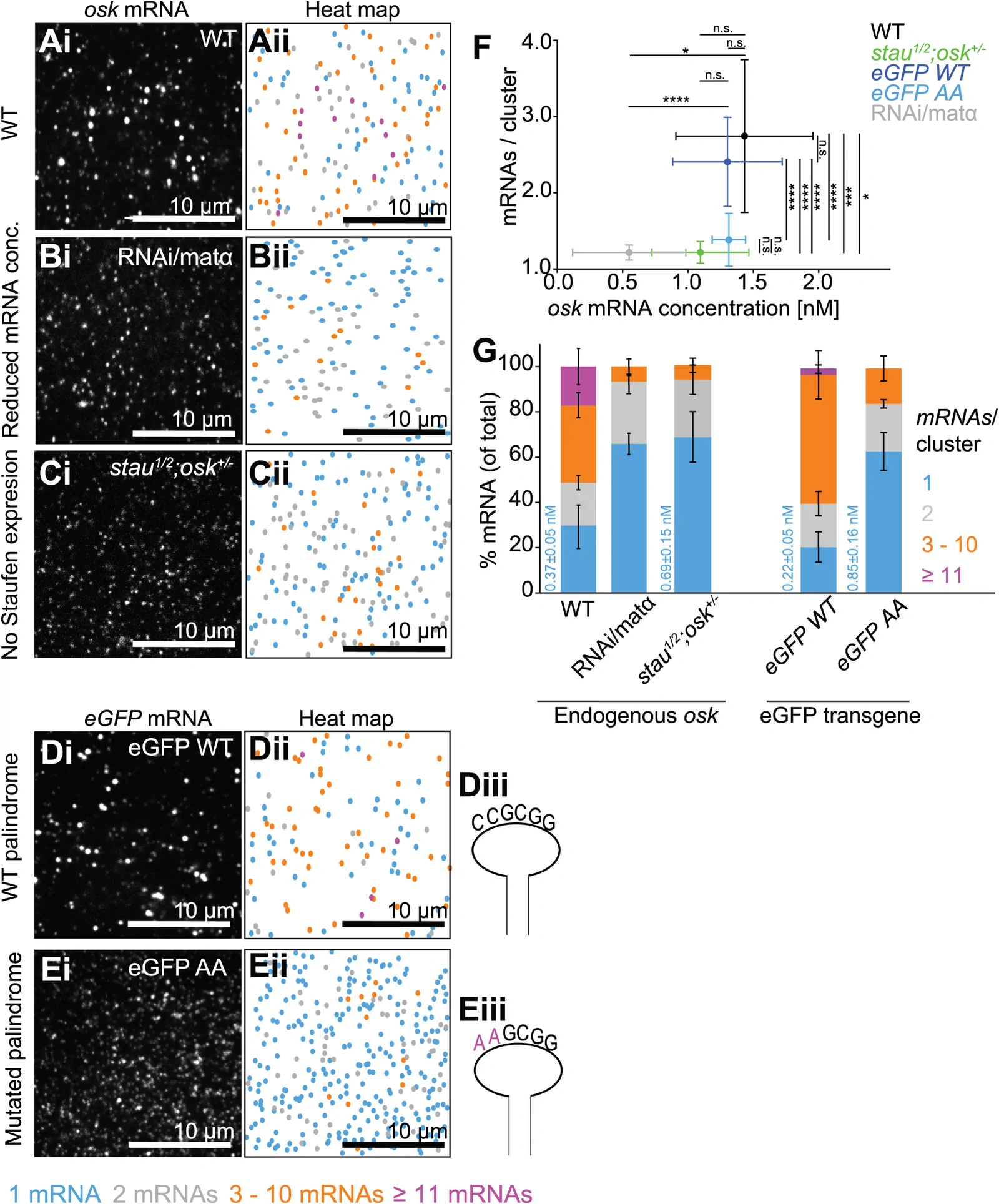
High *osk* mRNA concentration, Stau expression and the RNA palindrome drive *osk* clustering in vivo. **A–Ei–iii.** Maximal projection of five consecutive optical sections spaced 150 nm apart of a WT (Ai), RNAi/*matα* (Bi), *stau^1^/stau^2^; osk^±^* (Ci) embryos and those expressing *eGFP-WT* (Di) and *eGFP-AA* (Ei) transgenes. Corresponding heat maps depicting the spatial distribution of *osk* (Aii, Bii, and Cii), *eGFP-WT* (Dii), and *eGFP-AA* (Eii) mRNA clusters are shown. Schematic of the WT (Diii) and mutated (Eiii) exposed RNA sequence within the *osk* stem-loop. **F** Dependence of the average *osk*, *eGFP-WT* and *eGFP-AA* mRNA cluster size on its concentration (nM). Data represent mean ± STDEV from seven WT, four RNAi/*matα*, seven *stau^1^/stau^2^; osk^±^* embryos, 13 *eGFP-WT* and 12 *eGFP-AA* embryos. Statistical significance: two-tailed *t*-test. *: *P* = 0.01, ***: *P* = 0.002 and ****: *P* < 0.001. n.s.: not significant. **G** Distribution of *osk* mRNA across clusters of different sizes. Bars represent mean ± STDEV cluster size from seven WT, four RNAi/*matα*, and seven *stau^1^/stau^2^;osk^±^* embryos, 13 *eGFP-WT* and 12 *eGFP-AA* embryos. Blue numbers: the mean ± STDEV molarity of *osk*, *eGFP-WT,* and 12 *eGFP-AA* mRNA monomers (*n* = 7,7, 13, and 12 embryos, respectively) for each genotype is shown.

To assess the contribution of the *osk* palindrome to mRNA clustering in vivo, we analyzed the previously characterized engineered mRNAs in which the coding sequence (CDS) of eGFP was fused to the *osk* 3′UTR carrying either the WT *osk* CCGCGG palindrome (termed *eGFP-WT*) or mutated AAGCGG sequence (termed *eGFP-AA*; see also Figure 2, Diii and Eiii; Jambor *et al.*, 2011). We used 32 smFISH probes targeting the eGFP sequence to detect these transgenes and differentiate them from endogenous *osk* mRNA (Supplemental Table S1). Fitting the distribution of *eGFP-WT* and *eGFP-AA* fluorescent puncta to a Gaussian model revealed a population corresponding to a single mRNA for each transgene with nearly identical mean fluorescence intensities of 51.7 ± 1.4 and 50.0 ± 1.6 a.u., respectively (*P* < 0.0001, *R^2^*: 0.95 and *P* < 0.0001, *R^2^*: 0.97 for *eGFP-WT* and *eGFP-AA*, respectively; Supplemental Table S1). This highlighted the robustness and reproducibility of single mRNA detection by smFISH in vivo.

To further validate the accuracy of our single-molecule quantification, we compared *osk* mRNA concentration measured by smFISH with the value extrapolated from a standard curve. This curve was generated by correlating nanomolar (nM) concentrations of six reference mRNAs, namely *germ cell-less* (*gcl*), *tao1*, *ccr4*, *sra*, *pum*, and *eIF4G2*, as measured by smFISH, with their respective expression levels determined by RNA-sequencing data obtained from Flybase (mod *et al.*, 2010). These reference mRNAs were selected because we have previously shown that they predominantly appear as single molecules within the early embryo (Trcek *et al.*, 2015; Trcek *et al.*, 2020). Moreover, their lower expression levels minimized the potential for crowding, which could otherwise lead to undercounting and diminish the accuracy of mRNA concentration extrapolation.

We observed a strong correlation between mRNA expression levels determined by smFISH and RNA-sequencing for the six reference mRNAs (*R^2^* = 0.72; Supplemental Figure S1E), indicating that smFISH quantified mRNA concentrations across a range of gene expression levels, consistent with our previous findings (Trcek *et al.*, 2015; Trcek *et al.*, 2017). By fitting the relationship between RPKM values from RNA-seq (mod *et al.*, 2010) and smFISH-derived concentrations with a linear regression, we estimated the somatic concentration of *osk* mRNA to be 1.59 nM (Supplemental Figure S1E, green circle). This prediction closely matched the 1.43 ± 0.2 nM concentration measured by smFISH (Supplemental Figure S1E, magenta circle and error bars). These results indicated that our approach detected single *osk* mRNAs even in the presence of oligomerized species, allowing reliable quantification of *osk* mRNA concentration and its cluster sizes in vivo.

smFISH revealed that WT embryos formed a heterogeneous population of *osk* mRNA clusters, ranging from monomers to assemblies containing more than 11 *osk* transcripts (Figure 1E, i–iii). In contrast, *ccr4*, our control mRNA, predominantly formed monomers (Figure 1F, i–iii) even though *ccr4* clusters exhibited similar densities (defined as the number of clusters per volume unit) and slightly decreased cluster-to-cluster distances compared with *osk* (Figure 1, G and H). In addition, while the fluorescent intensity of *osk* clusters increased with cluster size, their diameters, measured in nanometers using a line scan, did not (Supplemental Figure S1F, i and ii). Instead, these diameters were comparable to those measured for *ccr4* clusters (Supplemental Figure S1G) and for 100 nm TertraSpeck microspheres (Supplemental Figure S1H), which were below the diffraction limit of light of our structured illumination microscope. Together, these results supported our conclusion that multiple *osk* mRNAs were organized within confined, diffraction-limited clusters within the early embryos.

Finally, mRNA clustering was specific to *osk*, as other mRNAs that were expressed at a similar or higher concentration than *osk* did not exhibit comparable clustering behavior (Figure 1I). Therefore, RNA clustering was an intrinsic property of *osk* mRNA, rather than a phenomenon driven by a limited optical resolution of the microscope.

Collectively, our results established robust and consistent detection of single *osk* mRNA using smFISH across distinct genetic backgrounds, providing strong confidence that the observed differences in *osk* cluster sizes reflect *bona fide* changes in cluster organization.

### High *osk* mRNA concentration and Stau expression increase the likelihood of *osk* clustering

Applying smFISH to WT embryos, we recorded an average *osk* cluster size of 2.74 ± 1.00 mRNAs (Figure 2, A, i and ii, and F, black circle and error bars), with 70.2% of *osk* mRNA found in clusters that contain more than one *osk* mRNA (Figure 2G). Therefore, unlocalized *osk* dimerized and oligomerized in WT embryos, consistent with previous observations (Little *et al.*, 2015; Trcek *et al.*, 2015).

Upon reducing the expression of *osk* by 2.6-fold in RNAi/*matα* embryos, *osk* cluster size was also reduced by 2.2-fold, a statistically significant difference (Figure 2, B, i and ii, and F, gray circle and error bars). Importantly, comparable *osk* concentrations yielded similar cluster sizes across different genetic backgrounds (Supplemental Figures S1B and S2, A and B), indicating that mRNA concentration rather than genetic background per se drove changes in *osk* clustering in RNAi/*matα* embryos.

In *stau^1^/stau^2^* embryos, generated by crossing flies carrying two distinct mutations in the *stau* gene, the average *osk* cluster size similarly decreased from 2.74 ± 1.00 mRNAs in WT early embryos to 1.61 ± 0.12 mRNAs in *stau^1^/stau^2^* embryos, a statistically significant change (Supplemental Figure S3A). However, loss of Stau also led to a 64% increase in total *osk* mRNA levels (Supplemental Figure S3, A and B). The mechanism behind this increase is unclear; however, it may reflect the Stau-mediated mRNA decay recorded in mammalian cells (Gong and Maquat, 2011). Therefore, to evaluate the role of Stau in *osk* clustering without the accompanying effect of *osk* mRNA concentration change, we combined the *stau^1^/stau^2^* mutations with the *osk* gene deficiency *Df(3R)p^XT103^* (Ephrussi *et al.*, 1991; hereafter “*stau^1/2^;osk^±^*”), which resulted in *osk* mRNA expression levels comparable to those recorded in WT embryos (Figure 2F, green circle and error bars, Supplemental Figure S3, A and B). Under these conditions, the average *osk* cluster size decreased by 55.5% from 2.74 ± 1.00 recorded in WT embryos to 1.22 ± 0.14 in *stau^1/2^;osk^±^* embryos, a statistically significant change (Figure 2, C, i and ii, and F). The distribution of *osk* cluster sizes paralleled that observed in RNAi/*matα* embryos (Figure 2G) despite *osk* concentration being approximately two-fold higher than the one recorded for RNAi/*matα* embryos (Figure 2F, green versus gray circle). These data indicated that high mRNA concentration and Stau expression both augmented *osk* clustering.

Next, we examined how *osk* cluster size depended on RNA concentration and Stau expression. Focusing first on oligomers, we observed that the fraction of oligomerized *osk* sharply decreased by 7.8- to 8.7-fold from 51.3% in WT to 6.6% and 5.9 ± 5.4% in RNAi/*matα* and *stau^1/2^;osk^±^* embryos, respectively (Figure 2G). Similar dependence of cluster size distribution on *osk* concentration was recorded in embryos with different genetic backgrounds (Supplemental Figure S2B). Additionally, commercially available overexpression lines generated only a modest 2.7-fold upregulation of *stau* mRNA levels (Supplemental Figure S3E), limiting our ability to further examine whether changes in Stau protein levels augment *osk* clustering. Instead, we investigated *stau^1^/stau^2^* embryos in which the proportion of dimerized *osk* doubled, from 18.9 ± 3.8% in WT early embryos to 40.7 ± 1.2% in *stau^1^/stau^2^* embryos (Supplemental Figure S3, C, i and ii, and D). In the absence of Stau, even with 64% higher total *osk* mRNA levels (Supplemental Figure S3, A and B), there were fewer oligomers as measured by the 50% reduction of oligomers in *stau^1^/stau^2^* embryos compared with WT (Supplemental Figure S3D). These results revealed that the transition of dimers into oligomers was severely impaired in the absence of Stau. Therefore, high RNA concentration and Stau both promoted *osk* mRNA oligomerization.

We then examined *osk* monomers and dimers. Reduced mRNA levels doubled the fraction of *osk* monomers in RNAi/*matα* embryos (Figure 2, B, i and ii, and G), consistent with a decreased probability of intermolecular encounters and *osk* dimerization at lower transcript abundance.

However, in *stau^1/2^;osk^±^* embryos, *osk* mRNA expression level was comparable to the one recorded in WT embryos (Figure 2F, green and black circles, Supplemental Figure S3, A and B), allowing us to compare changes in *osk* monomers and dimers directly. Specifically, we observed that the concentration of *osk* monomers doubled from 0.37 ± 0.05 nM to 0.69 ± 0.15 nM recorded for WT and *stau^1/2^; osk^±^* embryos, respectively (Figure 2G, blue numbers). A similar increase in monomer concentration was recorded in *stau^1^/stau^2^* embryos, which exhibited increased *osk* expression relative to WT (Supplemental Figure S3, A–D). Given this accumulation of *osk* monomers, which suggested impaired dimer formation, our data suggest that dsRBP Stau promoted *osk* dimerization in vivo by approximately doubling its probability.

Collectively, our data support a model in which dimers could still form in the absence of Stau, although at reduced frequencies compared with WT embryos, whereas their progression into larger *osk* mRNA clusters was much more severely impaired. These findings indicate that *osk* clustering is governed by both *osk* mRNA concentration and dsRBP Stau, with the higher-order oligomer formation being more acutely sensitive to perturbations in these factors than dimerization. This differential sensitivity suggests that dimerization and oligomerization rely on partially distinct combinations of intermolecular interactions, with oligomerization requiring additional interactions beyond those necessary for dimer formation.

### *osk* palindrome enhances mRNA dimerization and oligomerization

Next, we examined the contribution of the *osk* palindrome to mRNA clustering. In vitro, dimerization of the RNA derived from *osk* 3′UTR depends on a palindrome presented within a stem-loop structure (Figure 1B; Jambor *et al.*, 2011; Jambor *et al.*, 2014) and mutations that disrupt this sequence also abolish *osk* localization to the posterior pole in vivo (Jambor *et al.*, 2011). Additionally, substitution of the first two cytosines of the palindrome to adenosines increases the apparent dissociation constant (*K_d_*) for *osk* 3′UTR dimerization by threefold, from 90 nM observed for WT *osk* 3′UTR carrying the CCGCGG palindrome to ∼270 nM observed for *osk* 3′UTR carrying the mutated AAGCGG variant (Jambor *et al.*, 2011).

To assess this role in vivo, we analyzed clustering of *eGFP-WT*, carrying the CCGCGG palindrome, and *eGFP-AA* carrying the mutated AAGCGG sequence (Jambor *et al.*, 2011; Figure 2, D, i–iii, and E, i–iii). However, *osk* 3′UTR performs an essential noncoding function during oogenesis, and females that do not express *osk* do not lay eggs (Jenny *et al.*, 2006; Kanke *et al.*, 2015). Although the UAS-Gal4/*nanos* promoter system is active throughout oogenesis (Van Doren *et al.*, 1998), it did not drive sufficient expression of *eGFP-WT* and *eGFP-AA* transgenes in *osk^A87^/Df(3R)p^XT103^* females, which lacked endogenous *osk* RNA expression, to restore egg laying (Supplemental Figure S4, A and B).

Instead, we expressed both eGFP transgenes using the UAS-Gal4/*matα* promoter system. Unlike the UAS-Gal4/*nanos* system, which is weaker but active throughout oogenesis, UAS-Gal4/*matα* restricts transgene expression to late oogenesis and early embryogenesis (Curnutte *et al.*, 2023). As a result, the transgenes could not be analyzed independently of endogenous *osk*, necessitating that we examine their clustering alongside endogenous *osk* using spectrally distinct smFISH probes targeting their respective CDSs. Importantly, both *eGFP-WT* and *eGFP-AA* exhibited similar expression levels (Supplemental Figure S4, C and D), allowing us to compare the dimerization and oligomerization potential of *eGFP-AA* against *eGFP-WT*. We observed that while *eGFP-WT* mRNA localized at the posterior pole (fold enrichment of 1.96 ± 0.36, defined as the ratio of eGFP fluorescent levels between the posterior and outside, Supplemental Figures S4, E, i and ii, G, and H), *eGFP-AA* mRNA displayed minimal localization (fold enrichment of 1.20 ± 0.15, Supplemental Figure S4F, i and ii, G, and H). These data indicated that the intact palindrome is necessary for efficient localization of *osk* to the posterior, in agreement with published observations (Jambor *et al.*, 2011).

**FIGURE 3:**
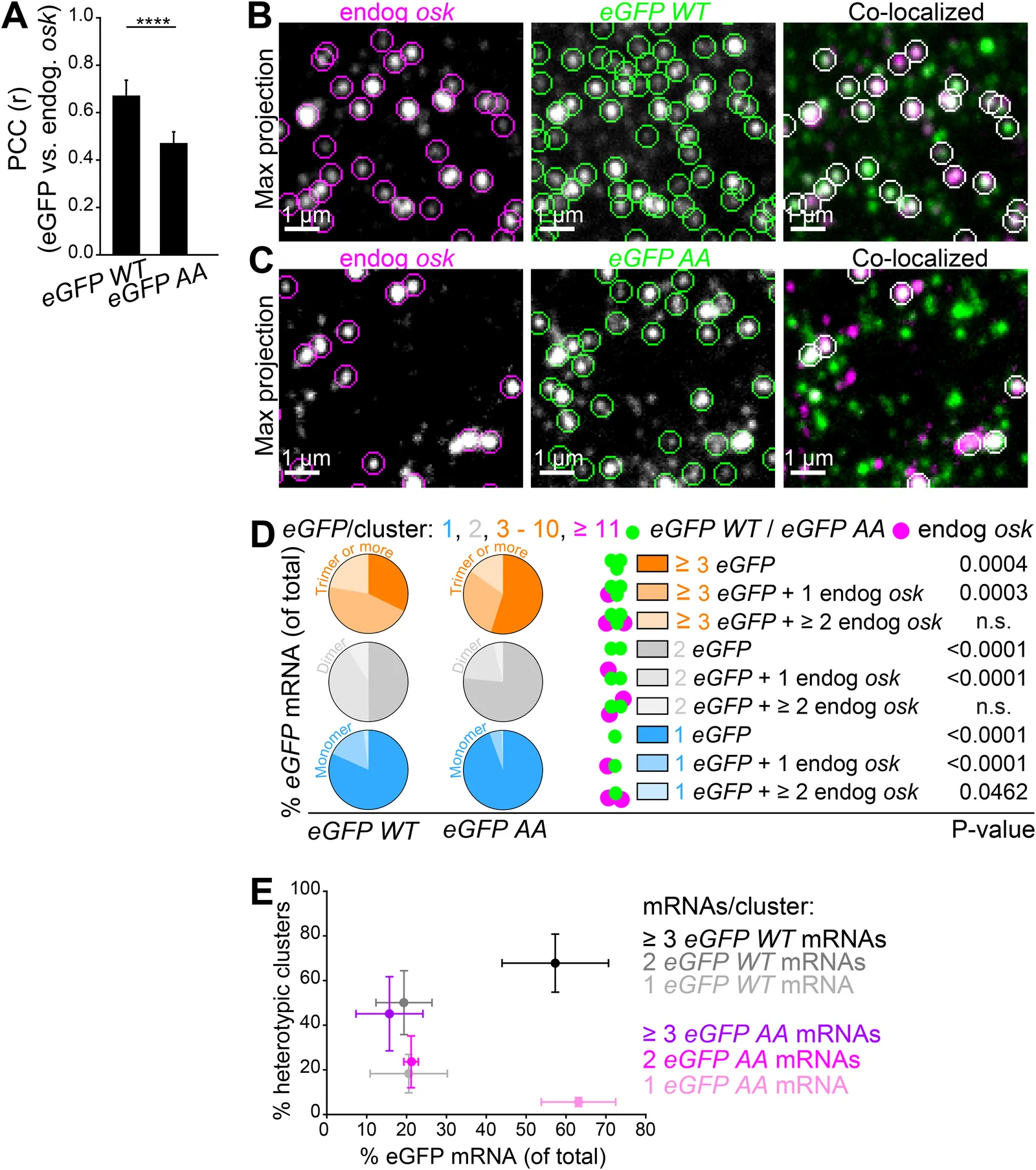
*osk* palindrome is a potent driver of heterotypic *osk* mRNA clustering. **A** Co-localization between endogenous *osk* and *eGFP-WT* or *eGFP-AA,* quantified with Pearson's correlation coefficient (PCC). Mean ± STDEV of PCC values from 13 *eGFP-WT* and 12 *eGFP-AA* embryos are shown. Statistical significance: two-tailed *t* test. ****: *P* < 0.001. **B and C** Representative images showing co-localization between endogenous *osk* (green) and *eGFP-WT* (C) or *eGFP-AA* (D) (magenta) with a 250 nm cutoff. Maximal projection of 5 consecutive optical sections of an ROI, spaced 150 nm apart are shown. **D** Pie charts show the composition of each *eGFP* cluster category based on its association with endogenous *osk* mRNA. For example, 20.6 ± 9.7% of *eGFP-WT* spots display the intensity expected for a single *eGFP-WT* mRNA (“monomers”; dark blue in the bar chart). Co-localization analysis using spot detection revealed that 90.4 ± 5.4% of these were true monomers (dark blue in the pie chart and legend), whereas 8.7 ± 4.4% and 1.0 ± 1.2% co-localized with endogenous *osk* dimers and oligomers, respectively (lighter blue shades in the pie chart and legend). Differences in heterotypic cluster size between *eGFP-WT* and *eGFP-AA* were evaluated using a two-tailed *t* test. **E** Fraction of *eGFP-WT* and *eGFP-AA* clusters with heterotypic composition per given *eGFP-WT* (black) and *eGFP-AA* (magenta) cluster size. For example, of the 63.1 ± 9.3% of *eGFP-AA* mRNA (magenta) identified as monomeric, 5.6 ± 2.0% were heterotically associating with endogenous *osk*, while 20.6 ± 9.7% of *eGFP-WT* mRNA identified as monomeric (black), 18.3 ± 8.6% were heterotipically associating with endogenous *osk*. Mean ± STDEV from 13 *eGFP-WT* and 12 *eGFP-AA* embryos is shown.

Clustering of *eGFP-WT* mirrored that of endogenous *osk* (Figure 2, A, i and ii, D, i and ii, and G): 79.4% of unlocalized *eGFP-WT* was found in clusters that contained more than one *eGFP-WT* mRNA (Figure 2G), yielding an average cluster size of 2.40 ± 0.58 mRNAs (Figure 2F, dark blue circle and error bars). This trend remained even after *eGFP-WT* clusters that associated with the endogenous *osk* mRNA were removed from the analysis (Supplemental Figure S4I; also refer to Figure 3). Therefore, like endogenous *osk*, unlocalized *eGFP-WT* mRNA efficiently dimerized and oligomerized.

In contrast, *eGFP-AA* showed impaired clustering, which mirrored that of *osk* in RNAi/*matα* embryos where the *osk* levels were reduced (Figure 2, B, i and ii, E–G). Only 36.9% of unlocalized *eGFP-AA* was found in clusters containing more than one mRNA (Figure 2G), yielding an average size of 1.31 ± 0.12 mRNAs, a statistically significant decrease compared with *eGFP-WT* (Figure 2F, light blue circle and error bars). Notably, this reduction occurred despite similar expression of *eGFP-AA* and *eGFP-WT* (1.4 ± 0.3 nM and 1.3 ± 0.4 nM, respectively; Figure 2F; Supplemental Figure S4, C and D). Consequently, the concentration of single *eGFP-AA* molecules nearly quadrupled, from 0.22 ± 0.05 nM for *eGFP-WT* mRNA to 0.85 ± 0.16 nM for *eGFP-AA* mRNA (Figure 2G, blue numbers). This trend remained after *eGFP-AA* clusters that associated with the endogenous *osk* mRNA were removed from the analysis (Supplemental Figure S4I; also refer to Figure 3). Notably, the magnitude of this decrease was comparable to the ∼three-fold reduction in dimerization potential for the *osk* 3′UTR carrying the mutated AAGCGG palindrome relative to the WT palindrome recorded in vitro (Jambor *et al.*, 2011).

These results revealed that *eGFP-AA* mRNA with a disrupted palindrome still dimerized, albeit nearly fourfold less efficiently than *eGFP-WT* containing a WT palindrome. Thus, the palindrome is required for efficient *osk* dimerization and subsequent oligomerization. Notably, although mutation of the palindrome reduced dimerization, higher-order oligomerization was affected much more severely, suggesting that oligomerization requires additional intermolecular interactions than those sufficient for dimerization.

Furthermore, mutation of the palindrome reduced dimerization approximately twofold more strongly than loss of Stau (Figure 2G), indicating that Stau alone cannot promote dimerization as effectively as the palindrome itself. Consistent with this interpretation, Stau was unable to fully compensate for the disruption of the palindrome. Thus, while both factors contribute to *osk* dimerization, the palindrome appears to play the more prominent role.

Taken together, these findings suggest that the palindrome and Stau make distinct, non-redundant contributions to *osk* dimerization, with efficient oligomerization requiring the contribution of both factors.

### The RNA palindrome is a potent driver of heterotypic *osk* mRNA clustering

To further assess the role of the palindrome in the clustering of *osk*, we analyzed the ability of *eGFP-WT* and *eGFP-AA* transgenes to heterotypically dimerize and oligomerize with endogenous *osk* using spectrally distinct smFISH probes targeting their respective CDSs.

Applying Pearson's Correlation Coefficient (PCC) analysis to evaluate co-localization, we found that *eGFP-WT* better co-localized with endogenous *osk* than *eGFP-AA* (Figure 3A), indicating that the palindrome enhances the likelihood for heterotypic mRNA clustering. Moreover, this difference in co-localization also further supported the previously reported in vivo role of the palindrome in mediating intermolecular base pairing (Jambor *et al.*, 2011).

Next, we analyzed the composition of *eGFP-WT* and *eGFP-AA* mRNA clusters. We quantified the number of *eGFP* monomers, dimers, and oligomers that co-localized with one, two, or more endogenous *osk* mRNAs. A 250-nm (nm) cutoff between the *eGFP* transgene and endogenous *osk* was used to define co-localization (Figure 3, B and C; *Materials and Methods* section; Supplemental Figure S5, A and B). This inclusive cutoff was based on our previous measurement showing that, in founder granules in embryos, *osk* forms clusters with a diameter of ∼100 nm (Trcek *et al.*, 2020), which corresponds to a spacing between neighboring *osk* clusters of approximately 100–150 nm.

We observed that of the 20.6  ± ** **9.7% of all *eGFP-WT* monomers (Figure 2G), 81.7 ± 8.6% were true monomers, with the remaining fraction co-localizing with one or more endogenous *osk* mRNAs (Figure 3D, blue pie chart). Similarly, of the 19.3  ±  7.0% and 60.1% of all *eGFP-WT* dimers and oligomers, respectively (Figure 2G), 49.9 ± 14.3% and 32.2 ± 13.0% formed homotypic clusters, respectively, while the remainder co-localized with endogenous *osk* mRNAs (Figure 3D, grey and orange pie chart, respectively).

In contrast, of the 63.1 ± 9.3% of all *eGFP-AA* monomers (Figure 2G), 94.4 ± 2.0% were true monomers, with the rest co-localizing with endogenous *osk* (Figure 3D, blue pie chart). Moreover, of the 21.1 ± 1.8% and 15.7% of all *eGFP-AA* dimers and oligomers, respectively (Figure 2G), 76.4 ± 11.6% and 54.9 ± 16.6% dimers and oligomers formed homotypic clusters, respectively, showing a statistically significant reduction in heterotypic interaction compared with *eGFP-WT* (Figure 3D, grey and orange pie charts, respectively). Importantly, this trend persisted even when the co-localization threshold was reduced to 100 nm (Supplemental Figure S5, C and D), indicating that the observed differences were robust to spatial resolution. Therefore, *osk* palindrome promoted heterotypic association between endogenous *osk* and the eGFP chimera.

Interestingly, the probability for heterotypic association applied differently to monomers, dimers, and oligomers. Specifically, even though monomers represented the majority (63.1 ± 9.3%) of *eGFP-AA* mRNAs, fewer than 6% formed heterotypic clusters with endogenous *osk* (Figure 3E). In contrast, while monomers represented only 20.6 ± 9.7% of *eGFP-WT* transcripts, they were 3.3-fold more likely than *eGFP-AA* mRNAs to form heterotypic clusters with endogenous *osk* (Figure 3E). Therefore, the palindrome was a potent enhancer of heterotypic dimerization.

In contrast, the fraction of *eGFP-AA* mRNAs that formed heterotypic clusters with endogenous *osk* increased by more than four-fold to 23.5% and 45.1%, respectively, even though these clusters contained less than 40% of all *eGFP-AA* mRNA (Figure 3E). These findings suggested that heterotypic association of a single *eGFP-AA* with endogenous *osk* is limited but becomes substantially more likely once *eGFP-AA* dimers and oligomers have formed. We observed a similar, although more pronounced trend with the *eGFP-WT* mRNA (Figure 3E).

These data suggested that the ability of *osk* monomers to form heterotypic interactions were strongly influenced by the *osk* palindrome, whereas heterotypic interactions involving dimers and oligomers were enhanced by additional interactions that may be driven by RBPs as well as other intermolecular base pairing interactions. This model is consistent with our observation that oligomerization of endogenous *osk* is mediated by multiple types of interactions, involving both RNAs and proteins (Figure 2).

Together, our results demonstrated that the RNA palindrome not only enhanced dimerization and oligomerization of *osk* mRNA but also promoted its heterotypic organization with mRNAs derived from distinct genetic loci.

### RNA palindromes may spatially organize other mRNAs in the early *Drosophila* embryo

Finally, we asked whether other *Drosophila* mRNAs may contain palindromes similar to *osk*. To address this, we identified all palindromes in transcripts of early embryos (NC 1 to 14) using an in-house script we described previously (Tian *et al.*, 2025). While RNA motifs consisting of only a few nucleotides may contribute to intermolecular interactions in vivo, we restricted our analysis to palindromes that were at least six nucleotides long as well as at least 50% GC-rich. In addition to resembling the *osk* palindrome, this threshold was supported by our previous work, which demonstrated using non-denaturing agarose gel electrophoresis that in vitro-transcribed RNAs containing shorter palindromes or palindromes with less than 66% GC content failed to dimerize (Tian *et al.*, 2025).

Using these criteria, we analyzed 11657 non-localizing transcripts (termed “ubiquitous”) representing isoforms of 4304 genes (Supplemental Table S2). Because *osk* localizes to the posterior, we reasoned that a similar palindrome-driven dimerization mechanism may contribute to the localization of other posterior-enriched mRNAs. Therefore, we separately analyzed 211 transcripts representing isoforms of 59 mRNAs previously identified as germ granule-enriched (termed “posterior localized”; Rangan *et al.*, 2009; Supplemental Table S2).

We observed that the total number of palindromes per transcript scaled linearly with transcript length (Supplemental Figure S6A) and that palindromes were modestly abundant across transcripts regardless of posterior localization (Supplemental Figure S6B). Therefore, the presence of the palindrome was not predictive of an mRNA's ability to enrich at the posterior pole. GC-rich palindromes accounted for less than half of the total palindrome density, indicating that palindromes were more frequently AT-rich than GC-rich (Supplemental Figure S6B).

**FIGURE 4:**
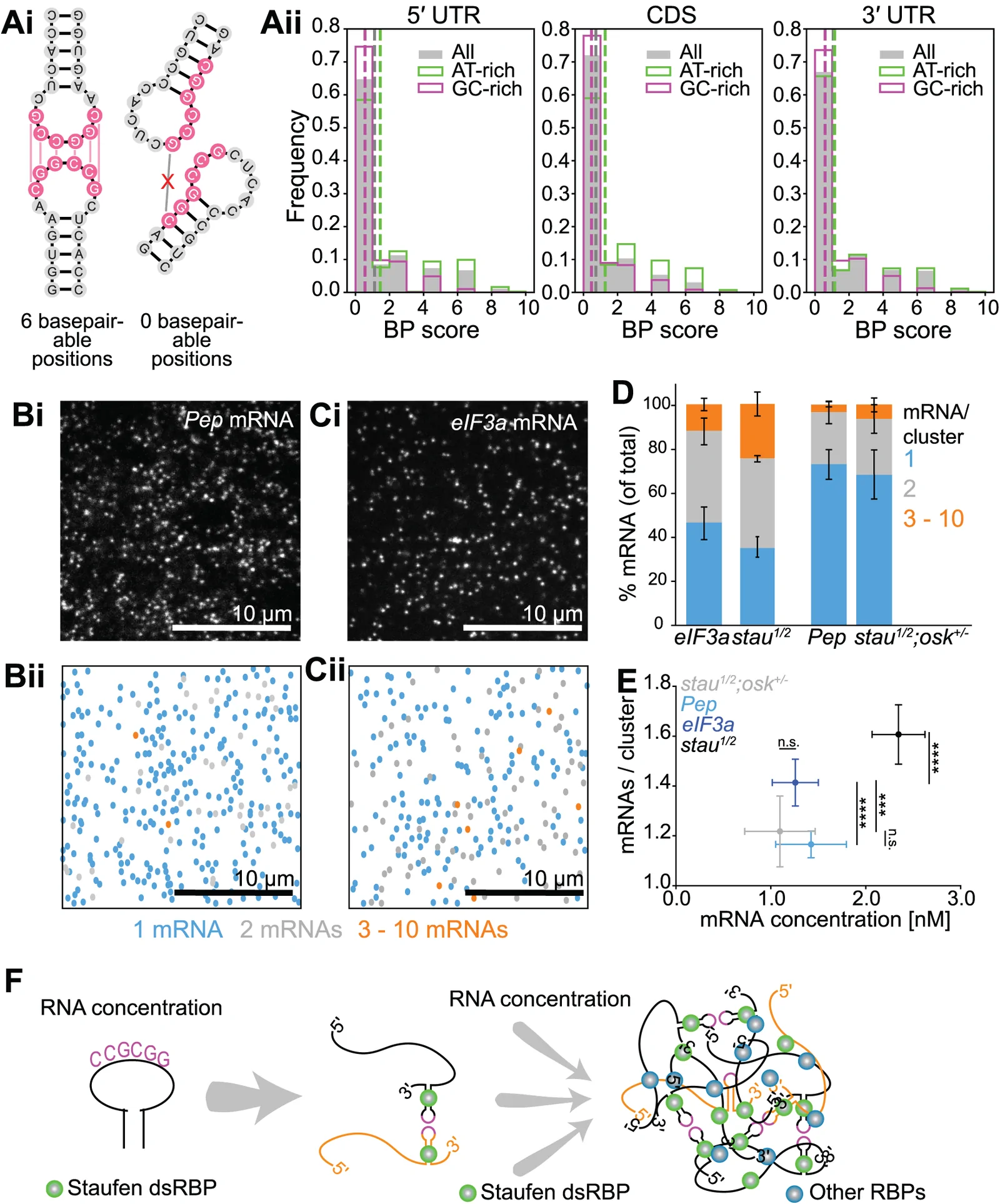
RNA palindromes may spatially organize other mRNAs in the early *Drosophila* embryo. **A, i and ii** Schematic of two palindromes presented atop an RNA stem-loop with 6 or 0 base-pairable positions (*Materials and Methods* section) (i). Histogram of the distribution of all (gray), AT-rich (green) and GC-rich (magenta) palindromes with a given number of base-pairable positions identified within the 5′UTR, CDS and 3′UTR of ubiquitous and posterior localized transcripts expressed in the early *Drosophila* embryo (ii). **Bi–Cii** Maximal projection of five consecutive optical sections of an ROI, spaced 150 nm apart, of *Pep* mRNA (Bi) and *eIF3a* mRNA (Ci) with corresponding heat maps depicting the spatial distribution of *Pep* and *eIF3a* mRNA clusters (Bii and Cii). **D** Distribution of *Pep* and *eIF3a* mRNA across clusters of varying sizes. For each cluster category, mean ± STDEV of 14 embryos per mRNA is shown. Data for *stau^1^/stau^2^* and *stau^1^/stau^2^; osk^±^* are from Supplemental Figure S3D and Figure 2G, respectively. **E** Dependence of average *osk* mRNA cluster size on its concentration (nM). For *Pep* and *eIF3a* mRNAs, data represent mean ± STDEV from 14 embryos. Data for *stau^1^/stau^2^* and *stau^1^/stau^2^; osk^±^* are from Supplemental Figure S2A and Figure 2F, respectively. Statistical significance: two-tailed *t* test. ***: *P* = 0.001; ****: *P* < 0.001. n.s.: not significant. **F** A model showing how the RNA palindrome, elevated mRNA concentration, and Stau dsRBP promote *osk* dimerization and subsequent oligomerization. Importantly, Stau and the palindrome may function as distinct, nonredundant contributors to *osk* mRNA dimerization and oligomerization, or may have interdependent activities. Moreover, the palindrome also markedly increases the likelihood of heterotypic *osk* association (depicted as interacting black and orange RNAs), indicating that the palindrome is a potent driver of heterotypic *osk* mRNA organization. Dimerization and oligomerization may be further supported by additional RBPs (blue circles) and intermolecular base pairing interactions not studied here.

Because the *osk* palindrome is both GC-rich and presented atop an RNA stem-loop (Figure 1B; Jambor *et al.*, 2011; Jambor *et al.*, 2014), we searched for GC-rich palindromes that resided within such RNA structures (*Materials and Methods* section). As the full secondary structure of the *Drosophila* transcriptome was not experimentally available, we approximated RNA structure using Vienna RNAfold. Due to the symmetrical nature of palindromes, we deemed a palindrome whose first half is embedded but second half exposed as not able to engage in intermolecular dimerization. This is because the base pairing partner of the second half is the first half. Therefore, to gain a more accurate understanding of how many palindromes may be able to dimerize, we considered the maximum number of consecutive nucleotides that were exposed in both palindrome halves, termed the “number of base-pairable positions” or “bp score” (Figure 4 Ai; Supplemental Table S2).

We observed that exposed palindromes with at least six base-pairable positions were mostly AT-rich, while those resembling the GC-rich palindrome of *osk* were extremely rare (Figure 4Aii). These findings are consistent with our recent study in which we mapped GC-rich palindromes onto the predicted RNA structure of 3′UTRs of germ granule-localized mRNAs *nanos*, *germ-cell-less* and *polar-granule-component* derived from in vivo chemical probing. Although all three contained a few GC-rich palindromes, these sequences were embedded within RNA structures and therefore unavailable for intermolecular base pairing (Tian *et al.*, 2025).

Looking at both ubiquitous and posterior localized mRNAs and their isoforms, we identified 60 (1.4%) mRNAs representing 155 (1.3%) isoforms that contained exposed, GC-rich palindromes (Supplemental Table S3). The majority of these palindromes were found in the CDS (in 38 out of 60 mRNAs and 101 out of 155 isoforms), while the 3′UTR had the least (in 9 out of 60 mRNAs and 19 out of 155 isoforms; Figure 4Aii; Supplemental Table S3). While none of these mRNAs were simultaneously highly expressed and predicted Stau targets (Supplemental Table S3), two mRNAs with GC-rich palindromes found in the CDS, *Pep* and *eIF3a*, nevertheless stood out as their expression levels recorded by RNA-seq were the most similar to the one recorded for *osk* (Supplemental Table S3).

smFISH analysis revealed that neither *Pep* nor *eIF3a* exhibited the strong clustering behavior characteristic of *osk*. Even so, 42.5 ± 6.8% and 11.3 ± 6.8% of *eIF3a* dimerized and oligomerized, respectively (Figure 4, B–D). The distribution of *eIF3a* cluster sizes was comparable to *osk* in *stau^1/2^* embryos (Figure 4D), which was noteworthy given that *eIF3a* expression was approximately two-fold lower than that of *osk* in *stau^1/2^* embryos (Figure 4E). In contrast, only 24.3 ± 6.0% and 2.8 ± 1.5% of *Pep* dimerized and oligomerized, respectively (Figure 4D), displaying a similar cluster size distribution observed for *osk* in *stau^1/2^;osk^±^* embryos (Figure 4, D and E). These data suggested that *eIF3a* mRNA may be a *bona fide* candidate transcript, whose spatial organization, although moderate, could be driven by intermolecular base pairing. Importantly, the early embryo may not provide an environment that would support abundant clustering of *eIF3a* as observed for *osk*. For example, *eIF3a* may require distinct dsRBPs, higher mRNA concentration, as well as additional intermolecular base pairing interactions for efficient clustering. Nevertheless, further experimental validation is needed to determine whether *eIF3a* represents a *bona fide* mRNA whose clustering is driven by palindrome-driven intermolecular base pairing in vivo.

In summary, our transcriptome-wide characterization revealed that while not widespread, exposed GC-rich palindromes may not be unique to *osk*. Our findings raise a possibility that palindrome-driven mRNA clustering may represent a previously underappreciated mechanism that contributes to mRNA organization during early *Drosophila* embryogenesis.

## DISCUSSION

### Persistent intermolecular base pairing requires the involvement of high RNA concentration and RBPs

Compared with in vitro studies using the isolated *osk* 3′UTR sequence (Jambor *et al.*, 2011; Bose *et al.*, 2024), the conditions that support intermolecular base pairing of *osk* RNA in vivo are more stringent and are driven by high RNA concentration, Stau, and the RNA palindrome. This principle closely mirrors RNA dimerization of HIV-1 (Moore and Hu, 2009).

HIV-1 contains a GC-rich palindrome (“GCGCGC” or “GUGCAC” depending on the subtype; Brunel *et al.*, 2002; Windbichler *et al.*, 2003) exposed within the SL1 stem–loop (Moore and Hu, 2009) that promotes HIV-1 RNA dimerization in vitro (Sakuragi *et al.*, 2016) and facilitates heterotypic RNA dimerization in cells (Moore *et al.*, 2007). However, as with *osk*, the palindrome alone is insufficient for efficient HIV-1 RNA dimerization, and additional factors participate. The HIV-1 nucleocapsid protein 7 (NCp7), which is produced by processing of the larger polyprotein Gag (Weiss *et al.*, 1984; Muriaux *et al.*, 1996) binds to the stem-loop containing the GC-rich palindrome (Cruceanu *et al.*, 2006; Abd El-Wahab *et al.*, 2014), and stabilizes RNA dimers in vitro (Darlix *et al.*, 1990; Feng *et al.*, 1996; Yuan *et al.*, 2003; Cruceanu *et al.*, 2006) and in cells (Chen *et al.*, 2016). Expression of Gag increases the population of dimeric RNAs by roughly three-fold (Chen *et al.*, 2016), paralleling the effect of Stau on *osk* dimerization in embryos (Figure 2). Thus, NCp7 and Stau appear to act analogously as molecular “glues” that stabilize palindrome-initiated RNA-RNA interactions. Notably, Stau and the palindrome may function independently to promote *osk* clustering, or they may act interdependently. Future analysis of the combined Stau/palindrome mutations is required to distinguish between these mechanisms and clarify their respective contributions to *osk* clustering.

Moreover, in vivo, dimerization of HIV-1 frequently occurs at the plasma membrane in the presence of the Gag protein (Chen *et al.*, 2016), where membrane association likely elevates local RNA concentration. Indeed, many RNA viruses assemble at sites of high RNA density, including transcription sites, the plasma membrane, or spherule invaginations derived from vesicles formed by the mitochondrial outer membrane (Maurel and Mougel, 2010; Chen *et al.*, 2016; Zhan *et al.*, 2023). This reinforces our observation that exposed base-pairing sequences and elevated local RNA concentration work together with RNA-binding proteins to enable efficient and sustained intermolecular RNA interactions in vivo.

### Multivalency and higher-order mRNA clustering

Despite their similarities, HIV-1 packages a defined RNA dimer into the virion, whereas *osk* transcripts assemble into clusters that may contain many mRNA copies (Figure 2A, i and ii; Little *et al.*, 2015; Eichler *et al.*, 2023). This difference likely reflects interactions promoted by NCp7 and Stau, other RBPs, as well as the RNA itself.

NCp7 contains two zinc finger motifs that primarily bind the stem-loop structure responsible for HIV-1 RNA dimerization, with some binding also detectable with the adjacent stem-loop (Dannull *et al.*, 1994; Muriaux *et al.*, 1996; Mailler *et al.*, 2016; Koma *et al.*, 2025). In contrast, Stau contains five dsRBDs (Figure 1Cii) and binds the palindrome-containing stem with dsRBD 3 (Figure 3Ci; Micklem *et al.*, 2000; Ramos *et al.*, 2000; Mohr *et al.*, 2021). This architecture suggests a model in which Stau stabilizes dimers through dsRBD3-mediated binding, while the remaining four dsRBDs could bridge additional *osk* transcripts, bringing them into proximity and enabling higher-order oligomerization.

Importantly, mutations in the palindrome reduce but do not abolish *osk* dimerization (Figure 2). This is consistent with in vitro studies showing that disruption of the palindrome does not abolish dimer formation of the isolated *osk* 3′UTR on gels (Jambor *et al.*, 2011; Bose *et al.*, 2024). These findings suggest that additional RNA elements may promote intermolecular RNA:RNA interactions even in the absence of the *osk* palindrome, in line with recent in vitro RNA phase-separation experiments using the *osk* 3′UTR (Bose *et al.*, 2024). Alternatively, the palindrome may mediate initial recognition, whereas other factors, including RBPs, stabilize the interaction. In this scenario, partial disruption of the palindrome would reduce, but not eliminate, dimerization.

Similarly to the mutations of the palindrome, loss of Stau strongly reduced, but did not abolish, *osk* dimerization and oligomerization (Figure 2, D, i and ii, and E, i and ii), indicating that additional factors contribute to this process. Two RBPs, the polypyrimidine tract-binding (PTB) protein and translational repressor Bruno, contribute to intermolecular *osk* interactions within founder granules at the posterior pole (Chekulaeva *et al.*, 2006; Besse *et al.*, 2009; Bose *et al.*, 2022; Bose *et al.*, 2024), and they could compensate for the lost Stau in the oligomerization of unlocalized *osk*. Both proteins exhibit multivalent RNA-binding capacity (Chekulaeva *et al.*, 2006; Snee *et al.*, 2008; Besse *et al.*, 2009; Reveal *et al.*, 2011; Bose *et al.*, 2022), and Bruno can also dimerize (Kim *et al.*, 2015), potentially reinforcing *osk* oligomerization.

Notably, *Drosophila* PTB contains four RNA recognition motifs capable of binding multiple PTB-binding sites within the *osk* 3′UTR (Besse *et al.*, 2009). Similarly, Bruno harbors three RBDs, each capable of engaging multiple Bruno response elements in *osk* (Chekulaeva *et al.*, 2006; Snee *et al.*, 2008; Reveal *et al.*, 2011; Bose *et al.*, 2022). Given the multivalent RNA-binding capacity of both PTB and Bruno, together with Bruno's ability to dimerize (Kim *et al.*, 2015) and the presence of multiple binding sites for these proteins within *osk* mRNA, collectively these proteins likely provide an additional interaction layer that reinforces oligomerization of unlocalized *osk*. Thus, *osk* clustering is supported by multivalency at both the RNA and protein levels.

### The palindrome as a determinant of compositional specificity

Interestingly, we observed that *eGFP-AA* mRNAs were 3.3-fold less likely than *eGFP-WT* mRNAs to dimerize with endogenous *osk* (Figure 3E), despite maintaining the binding sequences for Stau, Bruno or PTB (Bose *et al.*, 2024). This indicates that the palindrome itself is a potent driver of heterotypic mRNA:mRNA recognition. A similar principle operates in *Ashbya gossypii*, where base pairing between *BNI1* and *SPA2* mRNAs establishes compositional specificity of condensates associated with polarized growth (Langdon *et al.*, 2018). By analogy, our data support a model in which palindrome-mediated base pairing drives initial recognition and dimerization, while RBPs predominantly stabilize and expand assemblies.

Interestingly, a substantial fraction of reporter-derived oligomers remained homotypic (32.2 ± 13.0% for *eGFP-WT* and 54.9 ± 16.6% for *eGFP-AA*; Figure 3D), suggesting a possible demixing of oligomers as observed for mRNAs that enrich in *Drosophila* germ granules (Trcek *et al.*, 2015; Trcek *et al.*, 2020). Alternatively, dimerization and oligomerization may occur early in the *osk* mRNA life cycle. Both PTB and Bruno function in mRNA splicing in the nucleus and shuttle between the nucleus and cytoplasm (Wagner and Garcia-Blanco, 2001; Snee *et al.*, 2008; Spletter *et al.*, 2015). This raises the possibility that *osk* clustering could initiate co-transcriptionally, during or shortly after nascent transcript synthesis. This would mirror transcription-dependent dimerization proposed for the Murine leukemia virus (Maurel and Mougel, 2010). If clusters form co-transcriptionally at the site of synthesis, such assembly could ensure that endogenous *osk* and reporter alleles remain segregated once transported into the cytosol.

### RNA palindromes as a broader mechanism of mRNA organization

Our computational analysis identified 60 candidate mRNAs expressed in the early embryo that were predicted to contain palindromes similar to the one found in *osk* (Supplemental Table S3). Subsequent smFISH analysis confirmed that one of these, *eIF3a*, also exhibited light clustering behavior comparable to *osk* in *stau^1/2^* embryos (Figure 4D). Although none of these candidate mRNAs were both highly expressed and predicted Stau targets in early embryos, these parameters may change during later stages of development. Indeed, several transcripts reach expression levels comparable to or higher than *osk* at later developmental time points (Supplemental Table S3). Furthermore, efficient clustering of these mRNAs may require distinct double-stranded RNA-binding proteins (dsRBPs) as well as additional intermolecular base-pairing interactions. Notably, the *Drosophila* genome encodes at least 12 dsRBPs (DeSousa *et al.*, 2003), any of which could contribute to the spatial organization of specific transcripts during development.

Importantly, experimental validation is needed to determine whether these candidate mRNAs contain exposed palindromes that mediate mRNA clustering through intermolecular interactions in vivo. Additionally, limitations of our computational approach should also be considered. In particular, in silico RNA structure predictions may differ from structures measured by chemical probing in vivo (Rouskin *et al.*, 2014). Consequently, our RNAfold-based search may both overestimate and underestimate the number of *bona fide* candidate mRNAs whose clustering could be driven by palindrome-mediated intermolecular base pairing.

Indeed, our search failed to identify *osk*, despite evidence demonstrating that its exposed GC-rich palindrome mediates intermolecular interactions. Previous in vitro probing of the *osk* stem-loop structure revealed that in a subset of *osk* 3′UTR molecules, the first cytosine and last guanosine of the palindrome engage in intramolecular base pairing, leaving only the central four nucleotides available for intermolecular interactions (Jambor *et al.*, 2011). Because RNAfold reports ensemble structural predictions across the RNA population, this partial pairing reduces the predicted exposure of the palindrome below the six-nucleotide threshold used in our analysis, thereby excluding *osk* from the candidate list.

Despite these limitations, our study suggests that exposed GC-rich palindromes may be present in other maternally deposited mRNAs in the early *Drosophila* embryo. This may represent an important and previously underappreciated mechanism that contributes to mRNA organization and development of the early *Drosophila* embryo. More than 30% of maternally deposited transcripts exhibit spatial organization (Lecuyer *et al.*, 2007), where they play critical roles in early developmental patterning (Holt and Bullock, 2009; Chiappetta *et al.*, 2022). Thus, although exposed RNA palindromes may not be ubiquitous, they could nonetheless represent an important and previously underappreciated mechanism contributing to mRNA organization in the early *Drosophila* embryo, and potentially in other organisms.

In conclusion, intermolecular base pairing has emerged as a potent mechanism of mRNA organization. We find that although RNA palindromes can robustly promote intermolecular interactions in vivo, the cellular conditions that support such base pairing extend well beyond simple sequence complementarity. Thus, in vivo, robust mRNA clustering requires cooperation between multiple RNA motifs and RNA-binding domains. Importantly, this may impose regulatory stringency on intermolecular base pairing in cells, making RNA clustering a controlled and context-dependent process rather than a promiscuous outcome of sequence complementarity, as is often observed in vitro. This increased stringency may be critical to prevent unintended interference with translation, as reported for condensates formed by in vitro–transcribed RNAs (Trussina *et al.*, 2025).

## MATERIALS AND METHODS

### Fly lines

Flies were maintained on cornmeal molasses/yeast medium at RT or 25°C using standard procedures (Trcek *et al.*, 2017). The following fly lines were used: for imaging, embryos expressing Vasa:GFP transgene were used as “wild-type” (WT) flies (Trcek *et al.*, 2015); for egg hatching experiments, *w^1118^* (Bloomington; 5905) were used; *matα-gal4; matα-gal4 (*Curnutte et al., 2023*)*; *nosGal4VP16 (w+)/TM3* (Cinalli and Lehmann, 2013), *osk* RNAi (y[1] sc[*] v[1] sev[21]; P{y[+t7.7] v[+t1.8] = TRiP.GL01101}attP2; Bloomington; 36903); *osk^Def^* (Df(3R)*pXT103*/*TM3 Sb, Ser* (Lehmann and Nusslein-Volhard, 1986); *yw; osk^54^ st ry ss/TM3 Sb* (Lehmann and Nusslein-Volhard, 1991; Markussen *et al.*, 1995); *stau^1^ cn^1^ bw^1^*/*Cyo* (Bloomington; 1507), *cn^1^ stau^2^*/*Cyo* (Bloomington; 86573; St Johnston *et al.*, 1991); *osk^A87^*/ TM6B (gift from Elizabeth Gavis; Jenny *et al.*, 2006); w-; egfp-*oskar* 3′UTR-WT/*cyo* (Jambor *et al.*, 2011; termed *eGFP-WT*) and *osk* 3′UTR-AA/*cyo* (Jambor *et al.*, 2011; termed *eGFP-AA*), (both gift from Anne Ephrussi's lab; Jambor *et al.*, 2011); P{EP}stau[G2356] (w[*]; P{w[+mC] = EP}*stau*[G2356]/CyO, P{ry[+t7.2] = sevRas1.V12}FK1;; Bloomington; 26989) and P{EPg}*Spn55B*[HP25975] (w[*]; P{w[+mC] = EP}stau[G2356]/CyO, P{ry[+t7.2] = sevRas1.V12}FK1;; Bloomington; 22094).

### Fly crosses used to modulate *osk* mRNA concentration

To characterize the dependence of *osk* mRNA cluster size on mRNA concentration, we examined embryos laid by females in which *osk* expression was downregulated with Gal4-responsive UAS RNAi using a weak *nanos* (*nos*) promoter (Trcek *et al.*, 2020; termed “RNAi/*nosp*”) as well as in embryos laid by *osk^54^/Df(3R)p^XT103^* (termed *osk^54^/Df*) and *osk^54^/+* females; Supplemental Figures S1B and S2A). Here, *osk^54^* carries a nonsense point mutation that prevents Osk translation (Ephrussi *et al.*, 1991; Kim-Ha *et al.*, 1991), triggering nonsense-mediated mRNA decay (NMD; Kurosaki *et al.*, 2019), while *Df(3R)p^XT103^* is a deficiency that deletes the *osk* gene (Ephrussi *et al.*, 1991). In addition, we also examined embryos in NC 13-14 (termed “WT late”), in which *osk* mRNA levels have diminished due to decay (Eichler *et al.*, 2020).

We observed that RNAi/*nosp* and *osk^54^/+* embryos exhibited similar mRNA expression levels to WT embryos and accordingly formed *osk* mRNA clusters of similar sizes, ranging from 2.69 ± 1.06 (*osk^54^/+*) to 2.84 ± 0.68 (RNAi/*nosp*; Supplemental Figures S1B and 2A). Additionally, *osk^54^/Df* and WT late embryos exhibited similar mRNA expression to RNAi/*matα* and accordingly formed *osk* mRNA clusters with lower sizes ranging from 1.22 ± 0.09 (RNAi/*matα*) to 1.66 ± 0.28 (*osk^54^/Df*; Supplemental Figures S1B and S2A).

### Egg hatching assays

Approximately 20 *stau^1^/stau^2^*, their sibling *(stau^1or2^/cyo*), *stau^1^/stau^2^;osk^±^* and females were crossed with 10 *W^1118^* young males and maintained at 25°C for three days on standard cornmeal/agar media supplemented with yeast powder. Afterward, the flies were caged, supplied with an apple juice plate containing a dollop of yeast paste, and incubated at 25°C for 24 h to lay eggs (Trcek *et al.*, 2017). The apple juice plate was then collected, the yeast paste removed, and the number of eggs on it determined. The plate was then incubated at 25°C for an additional 48 h, after which the number of hatched eggs was counted. The hatching rate was determined by dividing the number of hatched eggs by the total number of eggs on the same plate.

### Egg laying assay

21 *eGFP-WT/nos-Gal4;osk^A87^/Df(3R)pXT^103^ (e.i. eGFP-WT), eGFP-AA/nos-Gal4;osk^A87^/Df(3R)pXT^103^ (e.i. eGFP-AA), eGFP-WT/nos-Gal4;osk^A87^/TM6B or eGFP-WT/nos-Gal4; Df(3R)pXT^103^/MKRS (e.i. WT-SIB)* and *eGFP-AA/nos-Gal4;osk^A87^/TM6B or eGFP-AA/nos-Gal4; Df(3R)pXT^103^/MKRS (e.i. AA-SIB)* three-to-four-day old females were crossed with 10 WT males an allowed to lay eggs on an apple juice plates for 24 h at 25°C supplemented with a dollop of yeast paste. The apple juice plate was then collected, the yeast paste removed, and the number of eggs on it determined.

### Embryo collection

Unless noted otherwise, caged flies were allowed to lay eggs at room temperature (RT) on a fresh apple juice plate containing a dollop of fresh yeast paste for 1.5 h, after which the embryos were dechorionated, fixed in 4% paraformaldehyde and the vitelline membrane removed by methanol cracking. Embryos were then stored in 100% methanol at 4°C until further use (Trcek *et al.*, 2015; Trcek *et al.*, 2017).

### Single-molecule fluorescent in situ hybridization (smFISH)

A total of 48 fluorescently labeled DNA probes were used to hybridize against *osk*, *Pep,* and *eIF3a* mRNA, and 32 were used for eGFP. This approach strongly increases signal-to-noise ratio and increases our detection sensitivity, as described previously (Trcek *et al.*, 2015; Trcek *et al.*, 2017; Trcek *et al.*, 2020; Curnutte *et al.*, 2023; Tian *et al.*, 2025). Subsequent hybridization of mRNAs with smFISH probes was carried out as described before (Trcek *et al.*, 2015; Trcek *et al.*, 2017; Trcek *et al.*, 2020). smFISH probe sequences against *osk*, *ccr4,* and *eGFP* were listed in Trcek *et al.* 2015. The following 48 smFISH probes against *Pep* were used (listed in the 5′ to 3′ direction):

ccgatccgaacgacgaaaga, taaacactccgcctatccaa, aaacgattctgcggatttcc, gttgacctttgcgttattca, taccacggaaagccatgttg, caaaattgcggttgcggttc, gtccaccatagttgttgttg, caaggcgacatgttcatacc, gtcgcatgttgttaccaaac, gattgttggccaagttgatg, ggttcctgaacagattgttc, aagtcgagaagcgatggtgg, accacgttggttgcgatttc, gtatggagactccttggtag, tagaacatgtcgttcggcac, atgtgcttcttgcacagatg, ccttgatgtggttctcgaaa, cacgcatcatcagatgggtg, aggcgatagctctcctcaat, ctcctgacggatcatgttgg, gatcgacttgagctgctctg, atgcgcttcaagcggtcaaa, gaagttcaggtcgcacatgg, acttgcgatgggtcgagatg, cttcttcagctgcaaatgtc, tgttgcactcaatgcacttg, tcaatgcgagtggcgaactc, cggacagcagatgagtatcg, ctcttcttcggtgctgatgg, tcttgaccggcttcttcttg, tttgtctcgtcagcagcttc, gtcgttgaagtcaaggatgc, cagttgtacttaggtaggcg, ctccagcttggagatcagac, tgcacaccgagcactcatag, acctcggtgtcgaagaactt, cgtacgggagtgaatctcgg, agaagttgcggtgatgagtc, gatcttggtatcgcttgatt, tcaaccttgcgcttcttacg, cgatggatcgtacagctcac, tcagcattatcatccaccat, aggagtttggactggagctg, cggagatggagaggcgtctg, tagtagcgattgtagcgacc, ttacgaattccttcgacgtc, tcctgggcgtgaatgtaaat, atctgttctttcttcatcct.

The following 48 smFISH probes against *eIF3a* were used (listed in the 5′ to 3′ direction):

gttgaggaacattcaagcgc, agattgcgaaagtcctcaga, ttcagtgggttgaaatccac, tagactgaatgcgcttgcac, ccgttctcaatgaagtccac, atgtaaggagtgagcagagc, cattatagtcacgtccttca, cctgagagatctgacgaatc, gcaagagacgctggaactta, atgttgcaaaaactggccag, accagcagtttttccaattc, atctgcatgtcattgtgacg, tgttcttctggtggtcaata, gtcagatcagtgccgaaata, cgagggcatcgattgcaaag, aaccaactgagagcggatct, ccgtgtcagaacagtagaca, ggttcgggtagacgattgaa, aatggtttaccatctggttg, tcgcgatccttgatctcatg, gtcgctgaagaatacgctgg, tttctgtcctcgatgatctt, cacgagcattattctgcttc, ttgcgctctctttcttcttg, gcttggatttcgttctgatg, ttctccttgagactcttctc, cgtctgcgagatttgctgaa, cagcttggaaagcatcttct, tccaacttcttaatgccttc, cgatttgagtttcgactgca, gcgttcgaagtagtcgattt, aaaagcggtatctcctccag, cttttcggccaagtattttt, cagaactccttgtccttaac, aatggcattctcgatgcgtg, ggatacatgcgcttgagacg, aagggcctcaaggaattcat, cttttcgacgtagagggagg, agagcagcctcaaacttttt, aataatgcggtcggctaagc, ctctttctcgcgaaggaagg, tgtgccaagcgaatctcttc, ttttcgtattgggcacgacg, ttccttgattttacgctcag, ttagatgcgcttggacggtc, tgtgaatctggctctcgtcg, cacttgtcatcgcgcgattg, catcaatgcggaaatcgcgg.

Probes were coupled to a fluorescent dye using an in-house labeling protocol as we described previously (Tian *et al.*, 2025).

### mRNA fold enrichment

To measure mRNA fold enrichment, defined as the ratio of the mRNA fluorescence between the posterior and outside, the total fluorescence intensity of a 3D ROI cropped from the two respective locations was determined. The ROI were of the same dimensions. The analysis was performed using ImageJ, as we have done previously (Tian *et al.*, 2025).

### Microscopy

Images were acquired in three dimensions (3D) with a 150 nm Z step with a vt-instant Structured Illumination Microscope (vt-iSIM; BioVision Technologies). The microscope was equipped with the 405 nm 100 mW, 488 nm 150 mW, 561 nm 150 mW, 642 nm 100 mW, and 445 nm 75 mW lasers, two ORCA-Fusion sCMOS cameras and the Leica HC PL APO 63x/1.30 GLYC CORR CS2, HC PL APO 63x/1.40 OIL CS2 and HC PL APO 100x/1.47 OIL CORR TIRF objectives as described before (Curnutte *et al.*, 2023).

### Line scan analysis to determine the dimensions and fluorescence intensity profiles

Line scans were performed as described previously (Trcek *et al.*, 2015). Briefly, for each cluster size, mRNA or bead, 20 line scans were obtained in ImageJ, and mean ± STDEV were plotted. 100 nm TetraSpeck microspheres (Thermo Scientific, T7279) were used.

### Quantifying nearest cluster-to-cluster distance

To quantify local *osk* WT and *ccr4* mRNA density, we performed a nearest-neighbor distance analysis on detected cluster coordinates. For each mRNA cluster, the Euclidean distance to its closest neighboring cluster was calculated in three dimensions. Coordinates were converted from pixel units to nanometers using voxel dimensions of 64.5 nm in x and y and 150 nm in z before distance computation. Nearest-neighbor distances were computed independently for each cluster without enforcing one-to-one assignment, allowing the same neighboring cluster to be identified for multiple reference clusters where applicable.

### mRNA concentration and cluster size measurements using smFISH

Somatic mRNA concentrations were quantified as described previously (Trcek *et al.*, 2015). In short, a 3D region of interest (ROI) with known spatial dimensions was cropped from the image and the absolute number of smFISH-labeled mRNAs within the ROI determined using Airlocalize (Lionnet *et al.*, 2011; Trcek *et al.*, 2017), allowing calculation of the nanomolar (nM) mRNA concentration within the ROI, as we have done previously (Trcek *et al.*, 2015; Trcek *et al.*, 2017).

To determine the average fluorescence intensity of a single smFISH-labeled *osk* mRNA, we initially focused on data obtained from WT embryos. We plotted the integrated fluorescence intensities of all detected spots as a histogram and fitted it to a single-peak (1) and two-peak (2) Gaussian curve using Sigmaplot, as we have done previously (Trcek *et al.*, 2017; Trcek *et al.*, 2020):




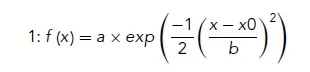







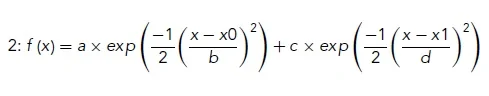




As the fluorescence intensity scales linearly with the number of fluorescently labeled probes within a diffraction-limited spot (Trcek *et al.*, 2017), we inferred the mRNA counts by normalizing the cumulative fluorescence intensity of each smFISH-labeled spot against the fluorescence intensity of a single *osk* molecule (i.e., X_0_ value), following established protocols (Trcek *et al.*, 2015; Trcek *et al.*, 2017; Trcek *et al.*, 2020).

The two-peak Gaussian curve provided a significantly better fit to our data than the single-peak Gaussian model (R^2^ of 0.96 vs. 0.75, respectively; Supplemental Table S1), which displayed a significant deviation from the measured data (Supplemental Figure S1A). This was anticipated as a considerable proportion of *osk* mRNAs formed clusters. For *osk* mRNA analyzed in WT embryos, this analysis generated mean ± STDEV fluorescence intensity of *osk* clusters containing one (X_0_) or two (X_1_) mRNAs (Supplemental Table S1), derived from fits in Supplemental Figure S1A. Fitting the distribution of *osk* fluorescent puncta for WT embryos to a two-component Gaussian curve revealed two *osk* populations, with mean fluorescence intensities of 154.3 ± 2.9 and 277.2 ± 25.0 a.u., which differed by approximately two-fold (*P* < 0.0001, R^2^:0.96, Supplemental Table S1). We interpreted these as representing clusters containing one and two *osk* mRNAs, respectively. Accordingly, for all subsequent analyses, we fitted the distribution of integrated fluorescence intensities of all detected spots to a two-peak Gaussian model. To estimate an absolute number of mRNAs within each fluorescent spot, we normalized the integrated fluorescence intensity of each smFISH-labeled spot to that of a single *osk* mRNA molecule (X_0_ value), as we have done previously (Trcek *et al.*, 2015; Trcek *et al.*, 2017; Trcek *et al.*, 2020).

To determine the size of mRNA clusters (defined as the number of mRNAs per cluster), we analyzed a 3D ROI as described above. Afterward, we determined the number of mRNAs in each cluster by normalizing its fluorescent intensity to the intensity of a single smFISH-labeled mRNA (Trcek *et al.*, 2015; Trcek *et al.*, 2017). Finally, to plot the spatial distribution of *osk* mRNA clusters as a heatmap, we used Sigmaplot as we have done previously (Trcek *et al.*, 2017; Trcek *et al.*, 2020). Notably, our interpretation of changes in *osk* mRNA clustering relies on fluorescence-intensity distributions. This approach provides greater phenotypic resolution when assessing differences between monomers and higher-order mRNA clusters, as they exhibit the largest difference in integrated fluorescence intensity.

### Quantifying total embryonic mRNA levels using qRT-PCR

Caged flies were provided with a fresh apple juice plate with a dollop of fresh yeast paste and allowed to lay eggs for 1.5 h, except for the WT late condition, which was aged for an additional hour. After dechorionation, eggs were washed with 1XPBS, and 200 µl TRIzol reagent (Invitrogen; 15596026) was added. Samples were homogenized using a pellet pestle motor (Kimble), followed by addition of 800 µl TRIzol. Samples were stored at −20°C until RNA extraction. Total RNA was purified using the Zymo RNA Clean & Concentrator kit (Zymo; R1018) according to the instructions. cDNA synthesis and qRT-PCR were performed as previously described (Tian *et al.*, 2025). *osk*, *stau,* and *Gapdh2* primer sequences were described previously (Curnutte *et al.*, 2023). The primer sequences are provided in the Supplemental Table S4. For analysis, *Act5C* or *Gapdh*2 was used as a control to calculate the relative transcript levels, which was 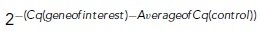.

### Co-localization analysis using spot detection

To quantify co-localization between endogenous *osk* mRNA and *eGFP* chimera (WT or AA) transcripts, images were acquired in three dimensions (3D) using an iSIM microscope with a 100x oil objective and subsequently corrected for chromatic aberration using Huygens (Scientific Volume Imaging), as done before (Trcek *et al.*, 2015; Trcek *et al.*, 2020). smFISH was performed using Quasar 670-labeled probes to detect endogenous *osk* and CalFl590-labeled probes to detect the *eGFP* chimera (WT or AA) transcripts (Trcek *et al.*, 2015). Spot detection for each fluorescence channel was carried out independently using an algorithm called Airlocalize (Trcek *et al.*, 2015; Trcek *et al.*, 2017). After thresholding, the algorithm produced 3D coordinates (x, y, and z) of all detected spots in pixel units, which were then converted to nanometers based on imaging parameters. Pairwise distances between endogenous *osk* and *eGFP* chimera (WT or AA) transcript spots were calculated using a nearest-neighbor greedy-matching approach, ensuring a one-to-one assignment between spots (Trcek *et al.*, 2015; Trcek *et al.*, 2020). Co-localized pairs were defined using distance cutoffs of 100 nm and 250 nm. Both cutoffs yielded consistent trends with the 250 nm cutoff data presented in the main text (Figure 3, B–D). Custom Python scripts, mRNA Spot Matcher and mRNA Colocalization Visualizer, were developed to identify and visualize co-localized spots, respectively. Both codes with detailed documentation are available in the X GitHub repository: https://github.com/ayseecer/mRNA_Spot_Matcher_and_Colocalization_Visualizer

### Identification of GC-rich palindromes in the *Drosophila* transcriptome

GC-rich palindromes are identified from fasta sequences downloaded from Flybase using an in-house script. Briefly, the fasta sequences of early *Drosophila* embryo genes are downloaded from Flybase in bulk, and GC-rich palindromes throughout each fasta sequence are identified as previously described (Tian *et al.*, 2025). ViennaRNA RNAfold was used to generate predicted secondary structures of each mRNA sequence. A full list of the identified palindromes is included in Supplemental Table S2. The code for identifying GC-rich palindromes in bulk and for scoring each palindrome's intermolecular base pairing propensity (“bp score”) is available in the GitHub repository: https://github.com/AnneyYeZiqing/pfind-bulk-palindrome-finder

## Supporting information





## Abbreviations used:

dsRBP: double stranded RNA binding protein
mRNAs: messenger RNAs
Osk: oskar protein
sk: oskar gene
smFISH: single molecule fluorescent in situ hybridization
stau: staufen gene
Stau: staufen protein.
