# Supplementary material for: In vivo clustering of oskar mRNA is driven by RNA concentration, RNA binding proteins, and an RNA palindrome
Source: Mol Biol Cell. 2026 Aug 12;37(10):ar92. doi: 10.1091/mbc.E26-04-0176 (PMC13549266; doi:10.1091/mbc.E26-04-0176)
Supplement: Supplementary file 1 [file mbc-37-ar92-s001.pdf]

Supplemental Materials

Molecular Biology of the Cell

Ye *et al*.

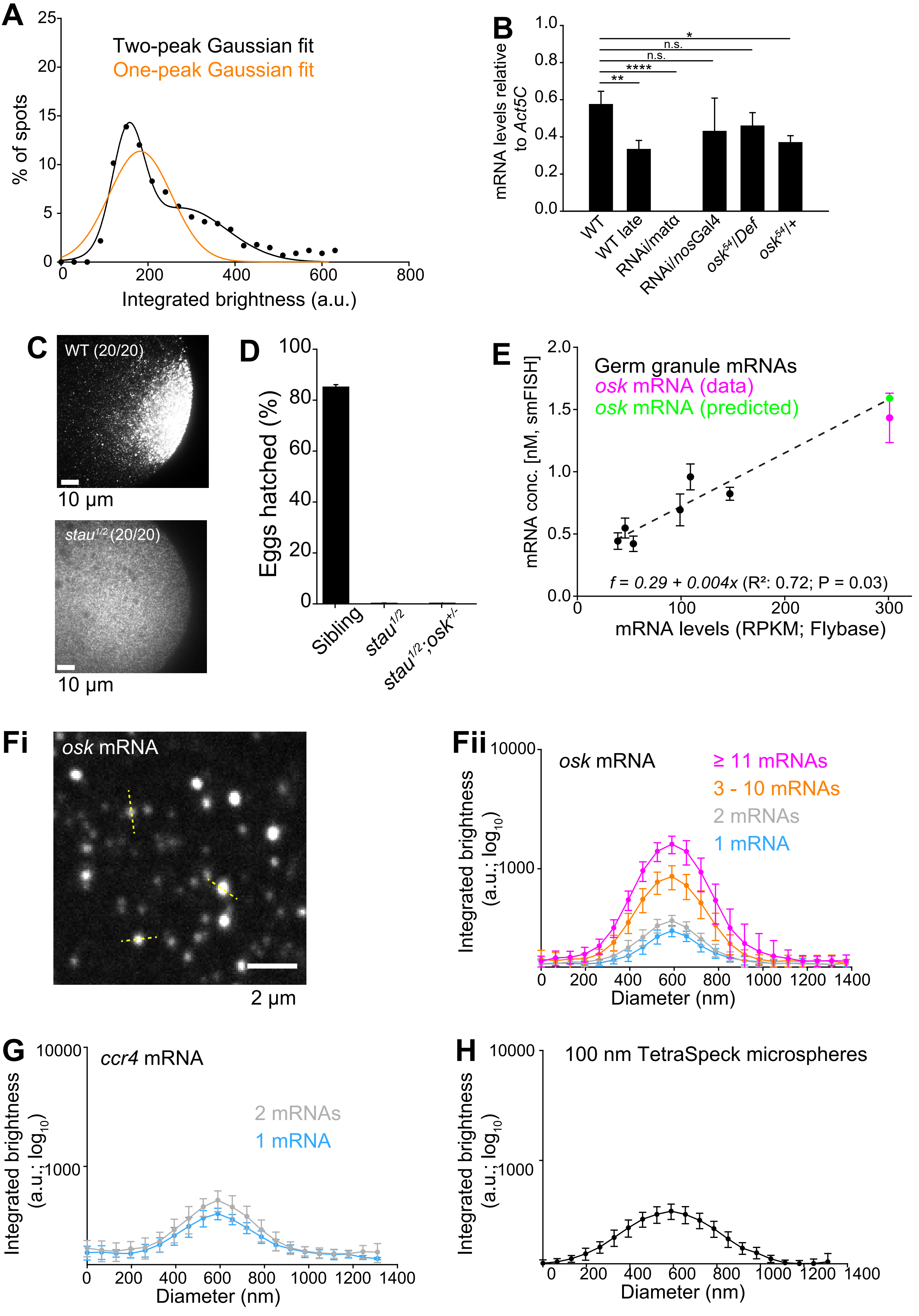

Supplemental Figure 1

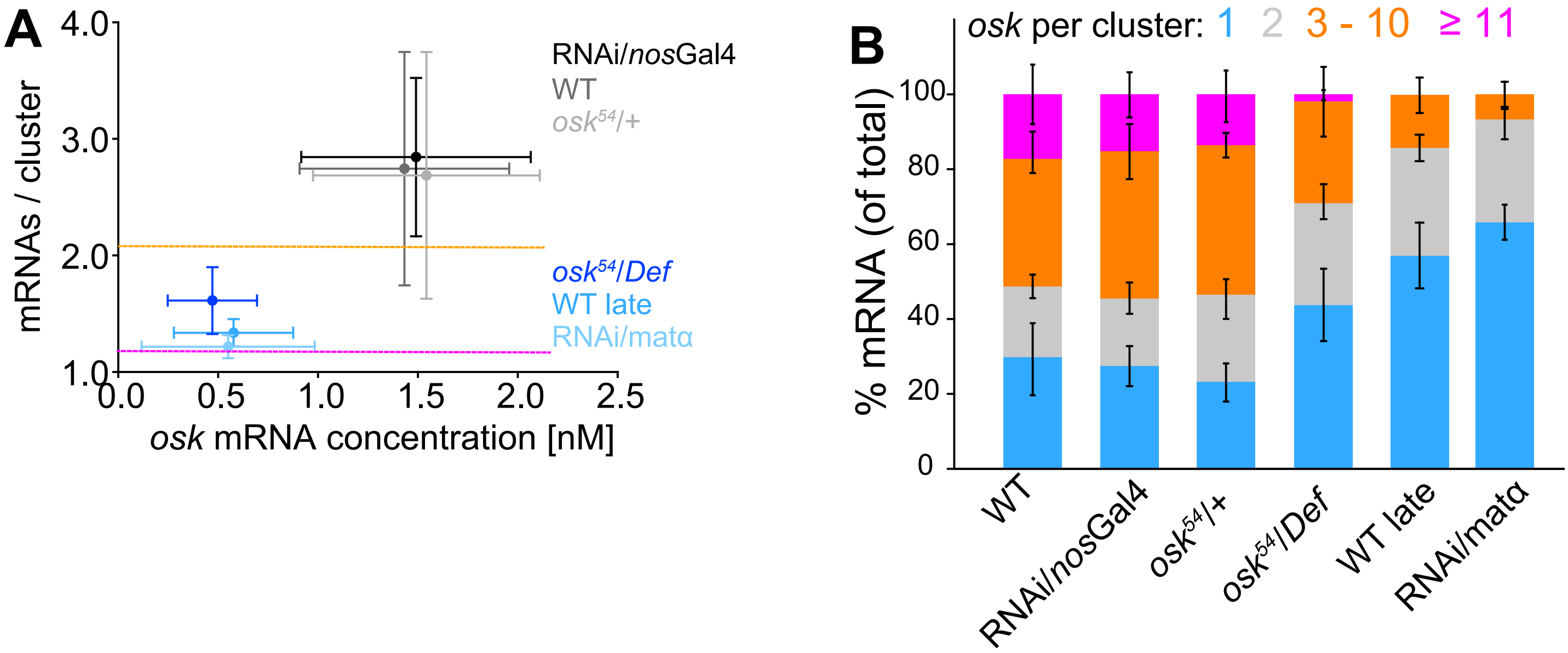

Supplemental Figure 2

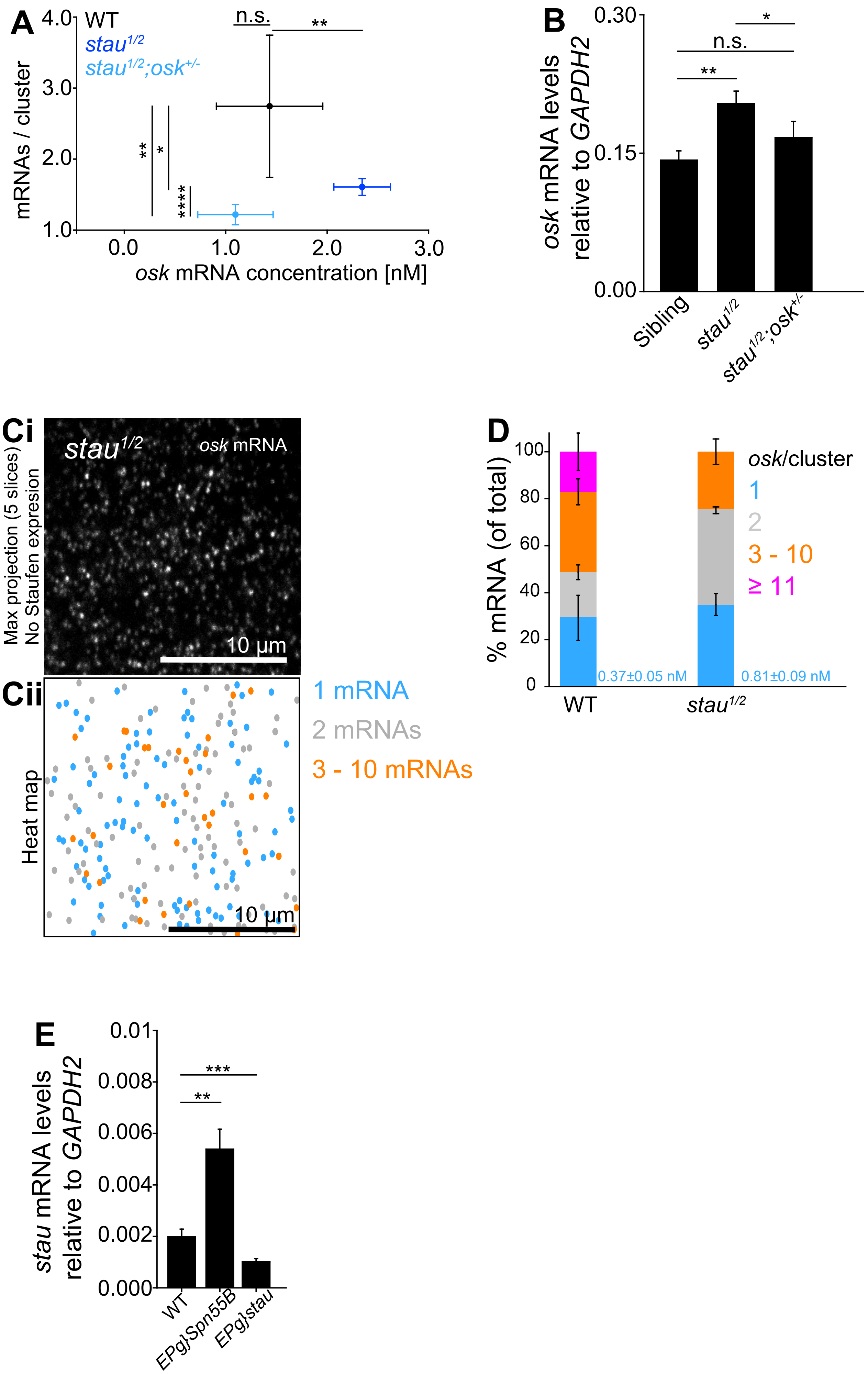

Supplemental Figure 3

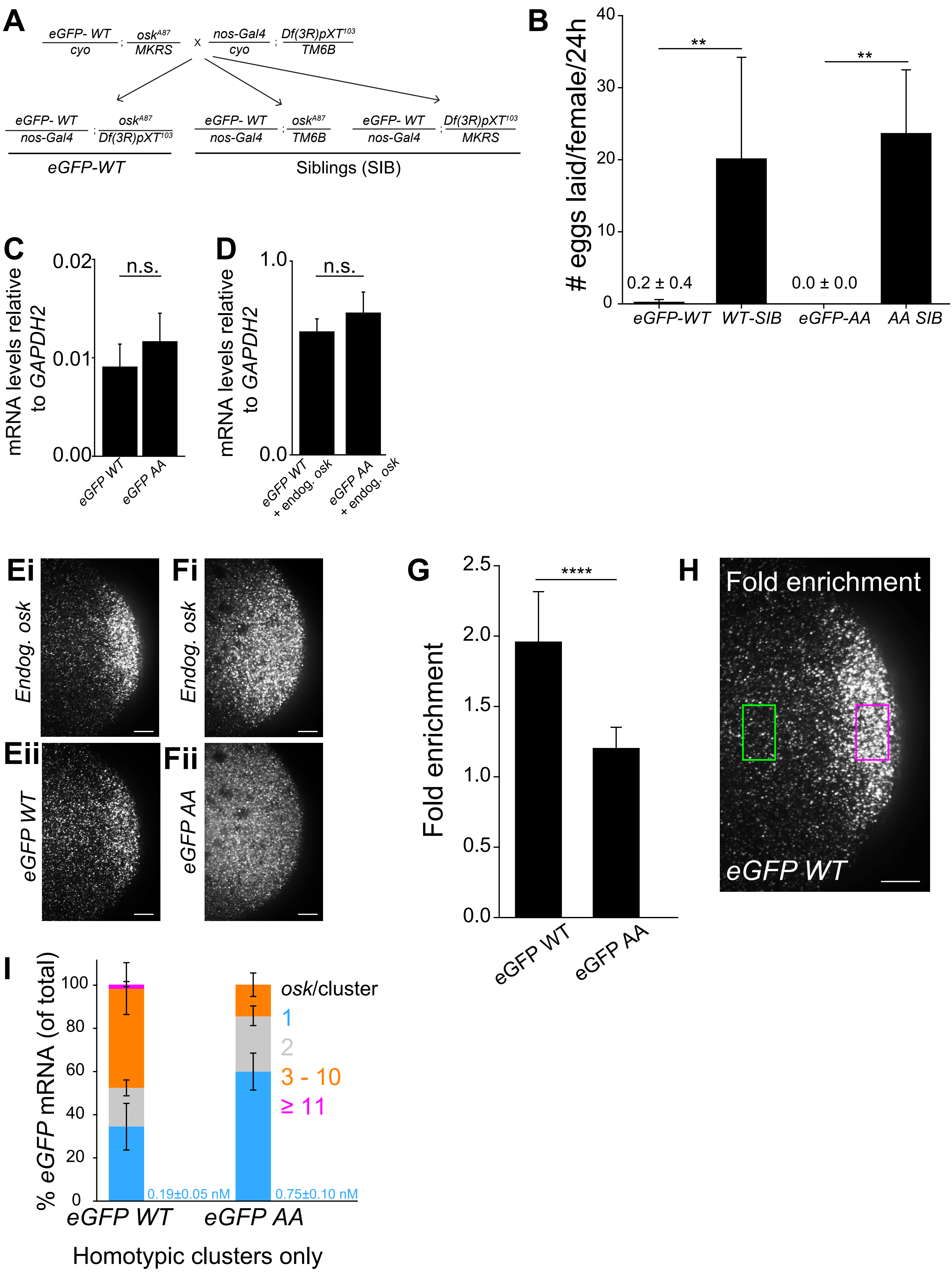

Supplemental Figure 4

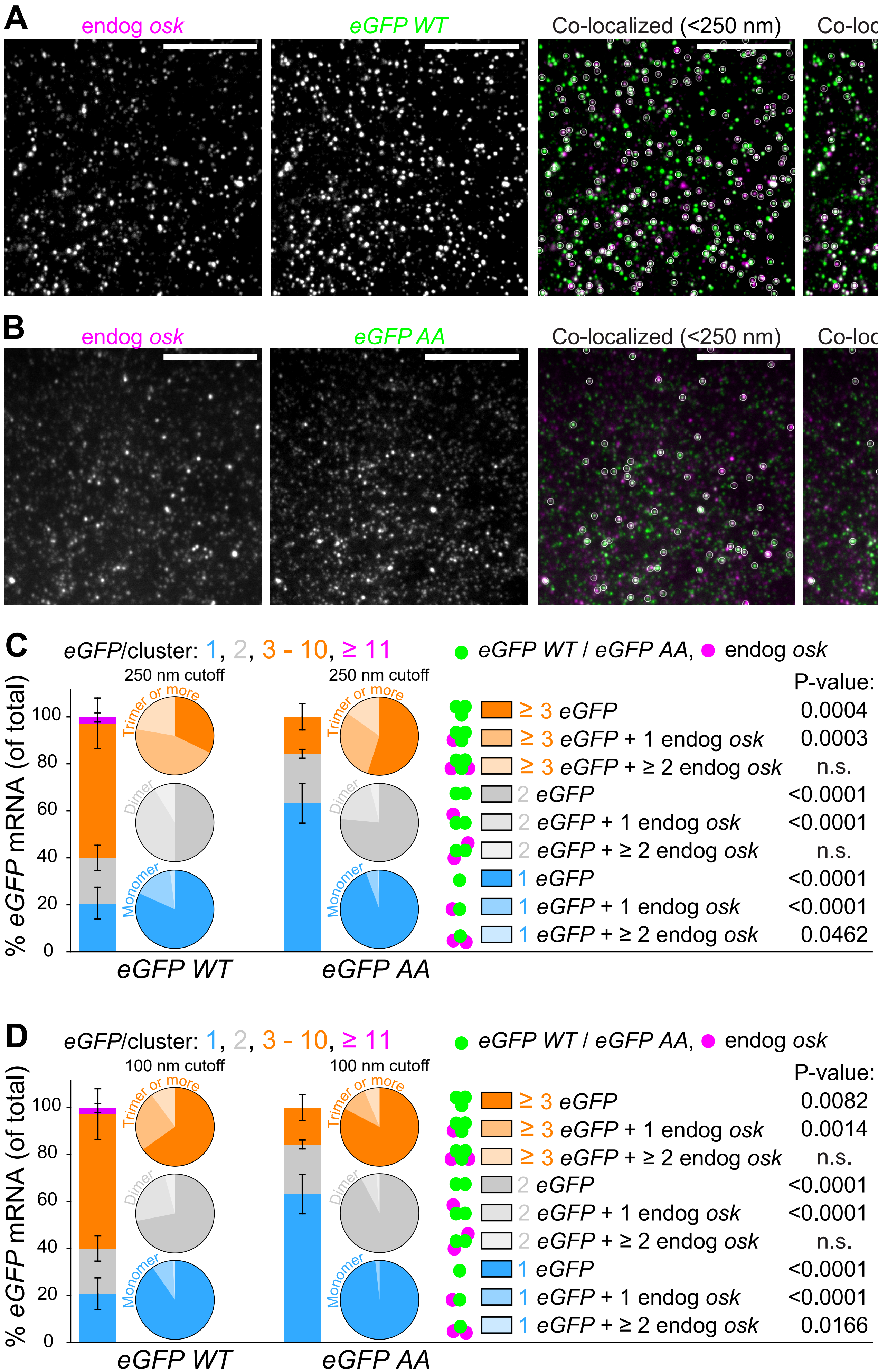

Supplemental Figure 5

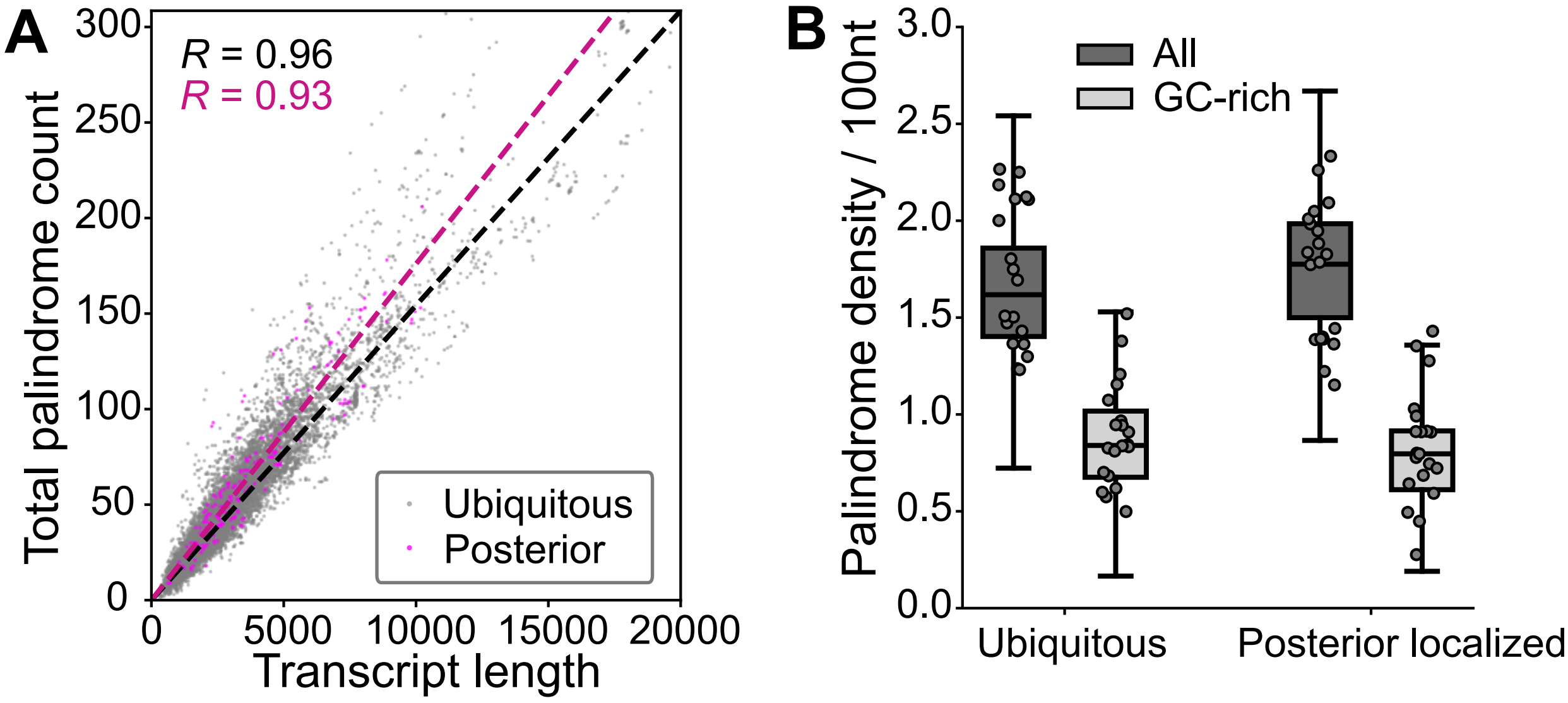

Supplemental Figure 6

Genotype	Gaussian fit	X0 (a.u.)		X1 (a.u.)		R^2
WT	2-peak	154.3±2.9	P<0.0001	277.2±25.0	P<0.0001	0.96
WT	1-peak	185.7±9.1	P<0.0001			0.75
RNAi/matα	2-peak	154.9±4.6	P<0.0001	262.4±95.0	p=0.01	0.97
stau $^{1/2}$	2-peak	153.0±4.6	P<0.0001	274.2±28.4	P<0.0001	0.95
eGFP-WT	2-peak	51.7±1.4	P<0.0001	114.5±12.7	P<0.0001	0.95
eGFP-AA	2-peak	50.0±1.6	P<0.0001	77.3±5.9	P<0.0001	0.97

**smFISH probes targeting:**	**# of probes**
osk CDS	48
osk CDS	48
osk CDS	48
osk CDS	48
eGFP CDS	32
eGFP CDS	32

transcript	startnt	endnt	sequence	ss length	structure	tr length	symbol	fbgn	numExpos
FBtr00763	289	298	CGCTGCAC	10	...)))).))	473	RasGAP1-F	FBgn00043	4
FBtr00763	321	330	TAGCCGG(	10	)))).)))((	473	RasGAP1-F	FBgn00043	1
FBtr00743	15	20	GCGCGC	6	((((((	531	Septin4-RF	FBgn02599	0
FBtr00743	16	21	CGCGCG	6	((((((	531	Septin4-RF	FBgn02599	0
FBtr00743	388	393	CAGCTG	6	..((((	531	Septin4-RF	FBgn02599	2
FBtr03322	165	170	CTGCAG	6	))((((	534	Pino-RC	FBgn00169	0
FBtr03322	411	416	AGCGCT	6	(((((.	534	Pino-RC	FBgn00169	1
FBtr03322	515	520	GCATGC	6	((.(((	534	Pino-RC	FBgn00169	1
FBtr00708	73	78	CGATCG	6	(....(	319	Tre1-RA	FBgn00466	4
FBtr00708	109	114	AGCGCT	6	(((((.	319	Tre1-RA	FBgn00466	1
FBtr03009	67	74	AGACGTCT	8	.......)	260	CG10077-F	FBgn00357	7
FBtr03009	114	119	ACCGGT	6	.).)))	260	CG10077-F	FBgn00357	2
FBtr03009	226	231	TGGCCA	6	))).))	260	CG10077-F	FBgn00357	1
FBtr03366	65	74	CGGAGCT(	10	)((((((((.	701	Tao-RI	FBgn0031(	1
FBtr03366	100	105	GCCGGC	6	((.(((	701	Tao-RI	FBgn0031(	1
FBtr03366	104	115	GCGGATAT	12	(((..(......	701	Tao-RI	FBgn0031(	8
FBtr03366	137	142	GCATGC	6	((((((	701	Tao-RI	FBgn0031(	0
FBtr03366	282	287	CTGCAG	6	..((((	701	Tao-RI	FBgn0031(	2
FBtr03366	288	293	CACGTG	6	...(((	701	Tao-RI	FBgn0031(	3
FBtr03366	308	315	CTAGCTAG	8	)).))..)	701	Tao-RI	FBgn0031(	3
FBtr03366	349	356	GCACGTG(	8	))...)))	701	Tao-RI	FBgn0031(	3
FBtr03366	354	359	TGCGCA	6	)))).)	701	Tao-RI	FBgn0031(	1
FBtr03366	453	458	CAGCTG	6	(((((.	701	Tao-RI	FBgn0031(	1
FBtr03366	467	472	TGGCCA	6	(((((.	701	Tao-RI	FBgn0031(	1
FBtr03366	478	483	CAGCTG	6	(((((.	701	Tao-RI	FBgn0031(	1
FBtr03366	515	520	TGGCCA	6	(((((.	701	Tao-RI	FBgn0031(	1
FBtr03366	623	630	CCAGCTG(	8	))))))..	701	Tao-RI	FBgn0031(	2
FBtr03366	659	666	GGTCGAC(	8	)))))...	701	Tao-RI	FBgn0031(	3
FBtr03316	188	193	CTCGAG	6	))).((	333	bru1-RF	FBgn00001	1
FBtr03316	297	302	GTCGAC	6	).)..)	333	bru1-RF	FBgn00001	3
FBtr00819	253	260	ACAGCTGT	8	).))))).	869	pum-RD	FBgn00031	2
FBtr00819	557	564	ACGCGCGT	8	..((((((	869	pum-RD	FBgn00031	2
FBtr00819	624	629	CAGCTG	6	)))))(	869	pum-RD	FBgn00031	0
FBtr00819	657	662	GAGCTC	6	))))))	869	pum-RD	FBgn00031	0
FBtr03336	14	19	TGGCCA	6	((((((	1019	pum-RG	FBgn00031	0
FBtr03336	403	410	ACAGCTGT	8	).))))).	1019	pum-RG	FBgn00031	2
FBtr03336	707	714	ACGCGCGT	8	..((((((	1019	pum-RG	FBgn00031	2
FBtr03336	774	779	CAGCTG	6	)))))(	1019	pum-RG	FBgn00031	0
FBtr03336	807	812	GAGCTC	6	))))))	1019	pum-RG	FBgn00031	0
FBtr03316	208	215	CGTTAACG	8	))..))))	456	bru1-RJ	FBgn00001	2
FBtr03018	533	538	TGGCCA	6	))))).	601	spir-RE	FBgn00034	1
FBtr03301	157	164	GCAGCTG(	8	))))))).	389	Hip14-RB	FBgn02598	1
FBtr03443	221	226	CGATCG	6	...)).	408	RhoGAP92	FBgn00387	4
FBtr03443	279	284	GCCGGC	6	))))))	408	RhoGAP92	FBgn00387	0
FBtr03078	533	538	TGGCCA	6	))))).	601	spir-RH	FBgn00034	1
FBtr03106	14	19	GCGCGC	6	(((((.	359	Bsg25D-R(	FBgn00002	1

FBtr03106	183	190	CACGCGTC	8	...(((((	359	Bsg25D-RG	FBgn00002	3
FBtr03106	314	319	CGATCG	6	...)))	359	Bsg25D-RG	FBgn00002	3
FBtr03106	345	350	CCCGGG	6	...)))	359	Bsg25D-RG	FBgn00002	3
FBtr00887	28	33	CTGCAG	6	((((((	230	gcl-RA	FBgn00056	0
FBtr00887	83	88	GCATGC	6	....))	230	gcl-RA	FBgn00056	4
FBtr00887	161	168	GCAATTGC	8	.....(((	230	gcl-RA	FBgn00056	5
FBtr00813	533	538	TGGCCA	6	))))).	601	spir-RA	FBgn00034	1
FBtr03301	289	298	CGCTGCAC	10	...)))).))	473	RasGAP1-F	FBgn00043	4
FBtr03301	321	330	TAGCCGGC	10	)))).)))((	473	RasGAP1-F	FBgn00043	1
FBtr03040	60	67	GCTTAAGC	8	((..(((.	208	Tao-RG	FBgn00310	3
FBtr03040	104	109	TGCGCA	6	)).)).	208	Tao-RG	FBgn00310	2
FBtr01145	351	356	TGCGCA	6	..))).	3478	Unr-RB	FBgn02633	3
FBtr01145	401	408	CAAGCTTG	8	...)))))	3478	Unr-RB	FBgn02633	3
FBtr01145	2008	2013	CCTAGG	6	((...(	3478	Unr-RB	FBgn02633	3
FBtr01145	3057	3064	CTTCGAAG	8	.......)	3478	Unr-RB	FBgn02633	7
FBtr00896	527	534	CAACGTTG	8	(...((((	1427	larp-RA	FBgn02616	3
FBtr00896	850	855	CTGCAG	6	)))).)	1427	larp-RA	FBgn02616	1
FBtr00896	965	970	CGGCCG	6	).((((	1427	larp-RA	FBgn02616	1
FBtr00896	966	973	GGCCGGCC	8	.((((((.	1427	larp-RA	FBgn02616	2
FBtr00896	1018	1023	CGCGCG	6	)).)))	1427	larp-RA	FBgn02616	1
FBtr00896	1087	1092	GCCGGC	6	(.((..	1427	larp-RA	FBgn02616	3
FBtr00896	1095	1102	CCGTACGG	8	((....))	1427	larp-RA	FBgn02616	4
FBtr00896	1186	1191	AGCGCT	6	))(((.	1427	larp-RA	FBgn02616	1
FBtr00896	1243	1248	CGCGCG	6	((((..	1427	larp-RA	FBgn02616	2
FBtr00896	1352	1357	GCTAGC	6	))).))	1427	larp-RA	FBgn02616	1
FBtr00896	1382	1387	GCCGGC	6	((((((	1427	larp-RA	FBgn02616	0
FBtr00896	1394	1401	CGCCGGCG	8	))))))))	1427	larp-RA	FBgn02616	0
FBtr00896	1397	1404	CGGCGCCG	8	))))).))	1427	larp-RA	FBgn02616	1
FBtr03329	40	47	CTTCGAAG	8	(.....((	461	Unr-RD	FBgn02633	5
FBtr03065	533	538	TGGCCA	6	))))).	601	spir-RF	FBgn00034	1
FBtr00790	14	19	GCGCGC	6	(((((.	359	Bsg25D-RE	FBgn00002	1
FBtr00790	183	190	CACGCGTC	8	...(((((	359	Bsg25D-RE	FBgn00002	3
FBtr00790	314	319	CGATCG	6	...)))	359	Bsg25D-RE	FBgn00002	3
FBtr00790	345	350	CCCGGG	6	...)))	359	Bsg25D-RE	FBgn00002	3
FBtr03455	351	356	TGCGCA	6	..))).	3478	Unr-RE	FBgn02633	3
FBtr03455	401	408	CAAGCTTG	8	...)))))	3478	Unr-RE	FBgn02633	3
FBtr03455	2008	2013	CCTAGG	6	((...(	3478	Unr-RE	FBgn02633	3
FBtr03455	3057	3064	CTTCGAAG	8	.......)	3478	Unr-RE	FBgn02633	7
FBtr00802	19	24	CCGCGG	6	(...((	610	Dlg5-RB	FBgn00323	3
FBtr00802	93	98	TGGCCA	6	((((..	610	Dlg5-RB	FBgn00323	2
FBtr00802	139	144	GTGCAC	6	((((..	610	Dlg5-RB	FBgn00323	2
FBtr00802	187	194	TTCCGGAA	8	...((((.	610	Dlg5-RB	FBgn00323	4
FBtr00802	194	201	ATCCGGAT	8	....((((	610	Dlg5-RB	FBgn00323	4
FBtr00802	297	302	GAGCTC	6	)))...	610	Dlg5-RB	FBgn00323	3
FBtr00802	316	321	CGCGCG	6	.))))(	610	Dlg5-RB	FBgn00323	1
FBtr00802	317	322	GCGCGC	6	))))((	610	Dlg5-RB	FBgn00323	0
FBtr00802	328	333	CAGCTG	6	((((((	610	Dlg5-RB	FBgn00323	0

FBtr00813	131	136	GCGCGC	6	)).(((	504	Arpc2-RA	FBgn00328	1
FBtr03330	90	95	TGGCCA	6	.....)	124	CG10077-I	FBgn00357	5
FBtr00704	94	99	TGGCCA	6	((((..	147	eIF2BÎ²-RA	FBgn00249	2
FBtr03366	19	24	CCGCGG	6	(...((	610	Dlg5-RD	FBgn00323	3
FBtr03366	93	98	TGGCCA	6	((((..	610	Dlg5-RD	FBgn00323	2
FBtr03366	139	144	GTGCAC	6	((((..	610	Dlg5-RD	FBgn00323	2
FBtr03366	187	194	TTCCGGAA	8	...((((.	610	Dlg5-RD	FBgn00323	4
FBtr03366	194	201	ATCCGGAT	8	....((((	610	Dlg5-RD	FBgn00323	4
FBtr03366	297	302	GAGCTC	6	)))...	610	Dlg5-RD	FBgn00323	3
FBtr03366	316	321	CGCGCG	6	.))))(	610	Dlg5-RD	FBgn00323	1
FBtr03366	317	322	GCGCGC	6	))))((	610	Dlg5-RD	FBgn00323	0
FBtr03366	328	333	CAGCTG	6	((((((	610	Dlg5-RD	FBgn00323	0
FBtr00747	132	141	CCGAATTC	10	)))..)))...	503	Tao-RE	FBgn0031(	4
FBtr00747	151	156	CTCGAG	6	.(((..	503	Tao-RE	FBgn0031(	3
FBtr00747	187	192	ACGCGT	6	.(..((	503	Tao-RE	FBgn0031(	3
FBtr00747	425	432	CCAGCTGC	8	(.......	503	Tao-RE	FBgn0031(	7
FBtr00747	461	468	GGTCGAC(	8	)(((...(	503	Tao-RE	FBgn0031(	3
FBtr00819	14	19	TGGCCA	6	((((((	1019	pum-RC	FBgn00031	0
FBtr00819	403	410	ACAGCTGT	8	).))))).	1019	pum-RC	FBgn00031	2
FBtr00819	707	714	ACGCGCG"	8	..((((((	1019	pum-RC	FBgn00031	2
FBtr00819	774	779	CAGCTG	6	)))))(	1019	pum-RC	FBgn00031	0
FBtr00819	807	812	GAGCTC	6	))))))	1019	pum-RC	FBgn00031	0
FBtr00832	56	61	ACGCGT	6	.)))))	307	sra-RA	FBgn00863	1
FBtr00832	81	92	TGGAAGC"	12	))))........	307	sra-RA	FBgn00863	8
FBtr00832	131	136	GTGCAC	6	(((.((	307	sra-RA	FBgn00863	1
FBtr00832	276	281	TGGCCA	6	(((...	307	sra-RA	FBgn00863	3
FBtr00869	65	70	GCGCGC	6	((...(	338	MESR4-RA	FBgn00342	3
FBtr00869	66	71	CGCGCG	6	(...((	338	MESR4-RA	FBgn00342	3
FBtr00869	240	247	GCAGCTG(	8	).))..))	338	MESR4-RA	FBgn00342	3
FBtr00869	311	316	GCATGC	6	((((((	338	MESR4-RA	FBgn00342	0
FBtr00803	90	95	CGTACG	6	((((((	1046	bru1-RD	FBgn00001	0
FBtr00803	113	118	TCGCGA	6	).))((	1046	bru1-RD	FBgn00001	1
FBtr00803	302	307	GGGCCC	6	((((((	1046	bru1-RD	FBgn00001	0
FBtr00803	633	638	GGATCC	6	((((((	1046	bru1-RD	FBgn00001	0
FBtr00803	921	926	GGATCC	6	))))))	1046	bru1-RD	FBgn00001	0
FBtr00779	123	128	GTCGAC	6	).))))	150	Pino-RB	FBgn00169	1
FBtr00780	92	97	AGCGCT	6	.(((..	653	dock-RB	FBgn00105	3
FBtr00780	265	270	CCTAGG	6	(....)	653	dock-RB	FBgn00105	4
FBtr00780	448	453	TCCGGA	6	((((.(	653	dock-RB	FBgn00105	1
FBtr00780	603	610	CCAGCTGC	8	)).)))))	653	dock-RB	FBgn00105	1
FBtr00780	627	632	CCGCGG	6	).)).)	653	dock-RB	FBgn00105	2
FBtr00766	17	22	GGCGCC	6	...)))	3538	Unr-RA	FBgn02633	3
FBtr00766	24	29	GCTAGC	6	)).)))	3538	Unr-RA	FBgn02633	1
FBtr00766	411	416	TGCGCA	6	..))).	3538	Unr-RA	FBgn02633	3
FBtr00766	461	468	CAAGCTTG	8	...)))))	3538	Unr-RA	FBgn02633	3
FBtr00766	2068	2073	CCTAGG	6	((...(	3538	Unr-RA	FBgn02633	3
FBtr00766	3117	3124	CTTCGAAG	8	.......)	3538	Unr-RA	FBgn02633	7

FBtr03312	318	327	TAGGCGC(	10	)))).)))).	360	Pi3K21B-R	FBgn0020(	2
FBtr03393	28	33	CTGCAG	6	(((...	275	gcl-RB	FBgn0005(	3
FBtr03393	83	88	GCATGC	6	....))	275	gcl-RB	FBgn0005(	4
FBtr03393	161	168	GCAATTG(	8	(((..(((	275	gcl-RB	FBgn0005(	2
FBtr03346	42	47	TGCGCA	6	))))).	914	aqz-RC	FBgn0286!	1
FBtr03346	188	193	ACGCGT	6	)).(((	914	aqz-RC	FBgn0286!	1
FBtr03346	286	291	TGCGCA	6	(((...	914	aqz-RC	FBgn0286!	3
FBtr03346	370	377	CTGATCAG	8	(((((.((	914	aqz-RC	FBgn0286!	1
FBtr03346	536	543	ACAGCTGT	8	...))).)	914	aqz-RC	FBgn0286!	4
FBtr00869	96	101	AGCGCT	6	.)).).	310	CG6805-R/	FBgn0034:	3
FBtr00869	262	267	GACGTC	6	).))))	310	CG6805-R/	FBgn0034:	1
FBtr00747	75	82	CCAGCTG(	8	(.......	153	Tao-RD	FBgn0031(	7
FBtr00747	111	118	GGTCGAC(	8	)(((...(	153	Tao-RD	FBgn0031(	3
FBtr00704	538	543	GAGCTC	6	..))))	722	CG2865-R/	FBgn0023!	2
FBtr03348	106	111	CGCGCG	6	))))).	523	RapGAP1-I	FBgn0264{	1
FBtr03348	118	123	TCGCGA	6	((.(((	523	RapGAP1-I	FBgn0264{	1
FBtr03051	392	397	GAGCTC	6	(((..(	689	pum-RF	FBgn0003:	2
FBtr03051	681	686	GCTAGC	6	).))).	689	pum-RF	FBgn0003:	2
FBtr03341	222	227	AGGCCT	6	))))((	382	cta-RB	FBgn0000:	0
FBtr00743	15	20	GCGCGC	6	((((((	700	Septin4-RI	FBgn0259!	0
FBtr00743	16	21	CGCGCG	6	((((((	700	Septin4-RI	FBgn0259!	0
FBtr00743	320	327	GCAGCTG(	8	((((((((	700	Septin4-RI	FBgn0259!	0
FBtr00743	557	562	CAGCTG	6	))))).	700	Septin4-RI	FBgn0259!	1
FBtr00819	392	397	GAGCTC	6	(((..(	689	pum-RE	FBgn0003:	2
FBtr00819	681	686	GCTAGC	6	).))).	689	pum-RE	FBgn0003:	2
FBtr00819	125	130	CACGTG	6	.....)	434	osk-RC	FBgn0003(	5
FBtr00819	188	193	CAGCTG	6	.))))(	434	osk-RC	FBgn0003(	1
FBtr00819	226	235	CCGCATG(	10	(((....)))	434	osk-RC	FBgn0003(	4
FBtr00819	325	330	GCCGGC	6	((.(((	434	osk-RC	FBgn0003(	1
FBtr03014	186	191	AGCGCT	6	..((((	1026	orb-RF	FBgn0004{	2
FBtr03014	437	444	CTGGCCA(	8	)))))...	1026	orb-RF	FBgn0004{	3
FBtr03014	508	513	GCCGGC	6	((((..	1026	orb-RF	FBgn0004{	2
FBtr03014	688	693	GACGTC	6	((((.(	1026	orb-RF	FBgn0004{	1
FBtr03014	730	737	GCCGCGG(	8	)))).(((	1026	orb-RF	FBgn0004{	1
FBtr03316	342	347	GGATCC	6	)))...	353	bru1-RG	FBgn0000:	3
FBtr03330	32	37	TGCGCA	6	(((...	55	del-RB	FBgn0086:	3
FBtr00819	42	47	TGCGCA	6	))))).	1298	aqz-RA	FBgn0286!	1
FBtr00819	188	193	ACGCGT	6	)).(((	1298	aqz-RA	FBgn0286!	1
FBtr00819	286	291	TGCGCA	6	(((...	1298	aqz-RA	FBgn0286!	3
FBtr00819	500	505	CAGCTG	6	.(((((	1298	aqz-RA	FBgn0286!	1
FBtr00819	920	927	ACAGCTGT	8	...))).)	1298	aqz-RA	FBgn0286!	4
FBtr00743	15	20	GCGCGC	6	((((((	677	Septin4-R(	FBgn0259!	0
FBtr00743	16	21	CGCGCG	6	((((((	677	Septin4-R(	FBgn0259!	0
FBtr00743	320	327	GCAGCTG(	8	((((((((	677	Septin4-R(	FBgn0259!	0
FBtr00743	534	539	CAGCTG	6	...)))	677	Septin4-R(	FBgn0259!	3
FBtr03433	39	46	CGCATGC(	8	((.(((((	665	Nost-RH	FBgn0259:	1
FBtr03433	334	341	TCAGCTGA	8	(((((..(	665	Nost-RH	FBgn0259:	2

FBtr03433	656	661	TGGCCA	6	.)))..	665	Nost-RH	FBgn0259	3
FBtr00843	3	8	TGGCCA	6	(..(((	908	orb-RA	FBgn0004	2
FBtr00843	90	99	TGCCGCG	10	((((((((.(	908	orb-RA	FBgn0004	1
FBtr00843	501	506	CAGCTG	6	))))))	908	orb-RA	FBgn0004	0
FBtr00843	554	563	TGGAGCT	10	).)))))...	908	orb-RA	FBgn0004	4
FBtr00843	673	678	AGGCCT	6	.)).))	908	orb-RA	FBgn0004	2
FBtr00843	676	685	CCTGCGC	10	.))).)..))	908	orb-RA	FBgn0004	4
FBtr00843	732	737	GCCGGC	6	).))))	908	orb-RA	FBgn0004	1
FBtr01002	161	166	AGCGCT	6	.(.(((	303	orb-RD	FBgn0004	2
FBtr01002	296	301	GTGCAC	6	))))..	303	orb-RD	FBgn0004	2
FBtr03029	8	13	GACGTC	6	((((..	157	Nost-RG	FBgn0259	2
FBtr03029	17	26	TCGTCGA	10	.)))))))))	157	Nost-RG	FBgn0259	1
FBtr03029	64	69	ACGCGT	6	))).((	157	Nost-RG	FBgn0259	1
FBtr00767	13	18	GGCGCC	6	(((((.	496	pbl-RB	FBgn0003	1
FBtr00767	71	76	ACGCGT	6	((....	496	pbl-RB	FBgn0003	4
FBtr00767	324	329	GCGCGC	6	))))))	496	pbl-RB	FBgn0003	0
FBtr00767	484	489	CTGCAG	6	.)))..	496	pbl-RB	FBgn0003	3
FBtr03025	14	19	GCGCGC	6	(((((.	359	Bsg25D-RE	FBgn0000	1
FBtr03025	183	190	CACGCGT	8	...(((((	359	Bsg25D-RE	FBgn0000	3
FBtr03025	314	319	CGATCG	6	...)))	359	Bsg25D-RE	FBgn0000	3
FBtr03025	345	350	CCCGGG	6	...)))	359	Bsg25D-RE	FBgn0000	3
FBtr03464	164	169	GCGCGC	6	)).(((	537	Arpc2-RB	FBgn0032	1
FBtr00780	502	507	GCATGC	6	))))).	1658	cbt-RB	FBgn0043	1
FBtr00780	543	548	CAGCTG	6	((.(((	1658	cbt-RB	FBgn0043	1
FBtr00780	735	740	TGGCCA	6	(.....	1658	cbt-RB	FBgn0043	5
FBtr00780	806	811	GACGTC	6	(.((..	1658	cbt-RB	FBgn0043	3
FBtr00780	913	918	GCATGC	6	)))(((	1658	cbt-RB	FBgn0043	0
FBtr00780	1153	1158	CAGCTG	6	.(((((	1658	cbt-RB	FBgn0043	1
FBtr00780	1325	1332	CGCCGGC	8	))))))((	1658	cbt-RB	FBgn0043	0
FBtr03336	392	397	GAGCTC	6	(((..(	689	pum-RH	FBgn0003	2
FBtr03336	681	686	GCTAGC	6	).))).	689	pum-RH	FBgn0003	2
FBtr03092	72	81	CCGAATTC	10	......))).	443	Tao-RH	FBgn0031	7
FBtr03092	91	96	CTCGAG	6	.((((.	443	Tao-RH	FBgn0031	2
FBtr03092	127	132	ACGCGT	6	.(..((	443	Tao-RH	FBgn0031	3
FBtr03092	365	372	CCAGCTG	8	(.......	443	Tao-RH	FBgn0031	7
FBtr03092	401	408	GGTCGAC	8	(.((....	443	Tao-RH	FBgn0031	5
FBtr00813	533	538	TGGCCA	6	))))).	601	spir-RB	FBgn0003	1
FBtr00760	192	197	CAGCTG	6	..(((.	985	chrb-RC	FBgn0036	3
FBtr00760	202	207	AGCGCT	6	...)))	985	chrb-RC	FBgn0036	3
FBtr00760	266	273	GCGGCCG	8	.)))).))	985	chrb-RC	FBgn0036	2
FBtr00760	798	805	ACGTACG	8	.)))))))	985	chrb-RC	FBgn0036	1
FBtr00760	969	976	GTTGCAA	8	)))))..)	985	chrb-RC	FBgn0036	2
FBtr03029	39	46	CGCATGC	8	((.(((((	665	Nost-RD	FBgn0259	1
FBtr03029	334	341	TCAGCTG	8	(((((..(	665	Nost-RD	FBgn0259	2
FBtr03029	656	661	TGGCCA	6	.)))..	665	Nost-RD	FBgn0259	3
FBtr00862	54	59	GCCGGC	6	..))))	473	exu-RA	FBgn0000	2
FBtr00862	184	189	CAGCTG	6	..))))	473	exu-RA	FBgn0000	2

FBtr00862	260	265	CCTAGG	6	..))))	473	exu-RA	FBgn0000(	2
FBtr00862	308	315	TCGTACGA	8	.)))).))	473	exu-RA	FBgn0000(	2
FBtr00803	188	193	CTCGAG	6	))).((	333	bru1-RA	FBgn0000:	1
FBtr00803	297	302	GTCGAC	6	).)..)	333	bru1-RA	FBgn0000:	3
FBtr03089	471	476	TGCGCA	6	((.(((	3598	Unr-RC	FBgn0263:	1
FBtr03089	521	528	CAAGCTTG	8	...)))))	3598	Unr-RC	FBgn0263:	3
FBtr03089	2128	2133	CCTAGG	6	((...(	3598	Unr-RC	FBgn0263:	3
FBtr03089	3177	3184	CTTCGAAG	8	.......)	3598	Unr-RC	FBgn0263:	7
FBtr03335	157	164	GCAGCTG(	8	))))))).	389	Hip14-RC	FBgn0259:	1
FBtr00717	2	7	CGCGCG	6	.(((((	195	mei-S332-I	FBgn0002:	1
FBtr00717	3	8	GCGCGC	6	((((((	195	mei-S332-I	FBgn0002:	0
FBtr00717	32	37	GTGCAC	6	))))((	195	mei-S332-I	FBgn0002:	0
FBtr03063	77	84	ACTGCAGT	8	..(((((.	477	larp-RH	FBgn0261(	3
FBtr03063	137	142	CAGCTG	6	..))))	477	larp-RH	FBgn0261(	2
FBtr03063	159	164	CGGCCG	6	.(((((	477	larp-RH	FBgn0261(	1
FBtr03063	184	189	TGGCCA	6	))))).	477	larp-RH	FBgn0261(	1
FBtr03063	227	232	CTGCAG	6	((((..	477	larp-RH	FBgn0261(	2
FBtr03005	188	193	CTCGAG	6	))).((	333	bru1-RE	FBgn0000:	1
FBtr03005	297	302	GTCGAC	6	).)..)	333	bru1-RE	FBgn0000:	3
FBtr03312	32	43	AACAGCG(	12	(.((.(((...(	553	Pi3K21B-R	FBgn0020(	5
FBtr03312	322	327	TCGCGA	6	..((((	553	Pi3K21B-R	FBgn0020(	2
FBtr03312	376	383	GCTATAG(	8	...)))))	553	Pi3K21B-R	FBgn0020(	3
FBtr03312	382	391	GCGATAT(	10	))..))))))	553	Pi3K21B-R	FBgn0020(	2
FBtr03312	388	393	TCGCGA	6	))))((	553	Pi3K21B-R	FBgn0020(	0
FBtr03312	465	472	GCTGCAG(	8	(((((...	553	Pi3K21B-R	FBgn0020(	3
FBtr00890	102	107	TGCGCA	6	((..((	869	myo-RC	FBgn0026:	2
FBtr00890	444	449	GCATGC	6	((((((	869	myo-RC	FBgn0026:	0
FBtr00890	707	714	TGTGCACA	8	)))))..)	869	myo-RC	FBgn0026:	2
FBtr00890	811	816	CTGCAG	6	)))))(	869	myo-RC	FBgn0026:	0
FBtr03366	132	141	CCGAATTC	10	)))..)))...	503	Tao-RJ	FBgn0031(	4
FBtr03366	151	156	CTCGAG	6	.(((..	503	Tao-RJ	FBgn0031(	3
FBtr03366	187	192	ACGCGT	6	.(..((	503	Tao-RJ	FBgn0031(	3
FBtr03366	425	432	CCAGCTG(	8	(.......	503	Tao-RJ	FBgn0031(	7
FBtr03366	461	468	GGTCGAC(	8	)(((...(	503	Tao-RJ	FBgn0031(	3
FBtr01107	276	281	TGGCCA	6	.(((..	591	CG11597-I	FBgn0036:	3
FBtr01107	277	286	GGCCATG(	10	(((....)))	591	CG11597-I	FBgn0036:	4
FBtr01107	329	336	TTGGCCAA	8	((((...(	591	CG11597-I	FBgn0036:	3
FBtr01107	568	573	GACGTC	6	))))))	591	CG11597-I	FBgn0036:	0
FBtr00890	102	107	TGCGCA	6	((..((	386	myo-RA	FBgn0026:	2
FBtr03329	479	484	GAGCTC	6	..))))	663	CG2865-RI	FBgn0023:	2
FBtr03073	124	129	GAGCTC	6	((((((	292	CG43367-I	FBgn0263:	0
FBtr03073	238	243	GCTAGC	6	((....	292	CG43367-I	FBgn0263:	4
FBtr03449	131	138	CGGCGCC(	8	((((...)	246	chrb-RE	FBgn0036:	3
FBtr03449	186	193	CGCCGGC(	8	)..))).(	246	chrb-RE	FBgn0036:	3
FBtr03449	205	210	GACGTC	6	.(((..	246	chrb-RE	FBgn0036:	3
FBtr03449	206	215	ACGTCGA(	10	(((....)))	246	chrb-RE	FBgn0036:	4
FBtr03449	220	229	GATGGCCA	10	))..))).))	246	chrb-RE	FBgn0036:	3

FBtr03344	3	8	TGGCCA	6	(..(((	908	orb-RG	FBgn00048	2
FBtr03344	90	99	TGCCGCG(	10	((((((((.(	908	orb-RG	FBgn00048	1
FBtr03344	501	506	CAGCTG	6	))))))	908	orb-RG	FBgn00048	0
FBtr03344	554	563	TGGAGCT(	10	).)))))...	908	orb-RG	FBgn00048	4
FBtr03344	673	678	AGGCCT	6	.)).))	908	orb-RG	FBgn00048	2
FBtr03344	676	685	CCTGCGCA	10	.))).)..))	908	orb-RG	FBgn00048	4
FBtr03344	732	737	GCCGGC	6	).))))	908	orb-RG	FBgn00048	1
FBtr00890	102	107	TGCGCA	6	((..((	510	myo-RD	FBgn0026	2
FBtr00890	452	457	CTGCAG	6	))))))	510	myo-RD	FBgn0026	0
FBtr03402	65	70	GCGCGC	6	((...(	338	MESR4-RD	FBgn0034	3
FBtr03402	66	71	CGCGCG	6	(...((	338	MESR4-RD	FBgn0034	3
FBtr03402	240	247	GCAGCTG(	8	).))..))	338	MESR4-RD	FBgn0034	3
FBtr03402	311	316	GCATGC	6	((((((	338	MESR4-RD	FBgn0034	0
FBtr00891	40	45	GACGTC	6	))))).)	297	CG31998-I	FBgn0051	1
FBtr00896	72	77	CAGCTG	6	))))))	612	eIF4G2-RA	FBgn0260	0
FBtr00896	154	161	GCAATTG(	8	).))))))	612	eIF4G2-RA	FBgn0260	1
FBtr00896	239	244	CGTACG	6	..))))	612	eIF4G2-RA	FBgn0260	2
FBtr00896	373	378	TCCGGA	6	))))))	612	eIF4G2-RA	FBgn0260	0
FBtr00896	596	605	CCTTCGAA	10	.)))))).))	612	eIF4G2-RA	FBgn0260	2
FBtr03348	259	264	CTGCAG	6	).))))	1178	RapGAP1-I	FBgn0264	1
FBtr03348	990	995	TCCGGA	6	..).))	1178	RapGAP1-I	FBgn0264	3
FBtr03348	1094	1099	CTGCAG	6	))))).)	1178	RapGAP1-I	FBgn0264	1
FBtr03065	96	101	AGCGCT	6	.)).).	310	CG6805-RI	FBgn0034	3
FBtr03065	262	267	GACGTC	6	).))))	310	CG6805-RI	FBgn0034	1
FBtr03011	136	143	GCAATTG(	8	((..((((	622	larp-RD	FBgn0261	2
FBtr03011	320	331	CGTGTTAA	12	((((....))))	622	larp-RD	FBgn0261	4
FBtr03011	364	369	CGTACG	6	))))).	622	larp-RD	FBgn0261	1
FBtr00743	44	49	GCGCGC	6	((((.(	225	Septin4-RA	FBgn0259	1
FBtr00743	45	50	CGCGCG	6	(((.((	225	Septin4-RA	FBgn0259	1
FBtr00743	82	87	CAGCTG	6	(.((((	225	Septin4-RA	FBgn0259	1
FBtr00779	165	170	CTGCAG	6	)(((((	483	Pino-RA	FBgn0016	0
FBtr00779	411	416	AGCGCT	6	..))))	483	Pino-RA	FBgn0016	2
FBtr03106	14	19	GCGCGC	6	(((((.	359	Bsg25D-RF	FBgn0000	1
FBtr03106	183	190	CACGCGT(	8	...(((((	359	Bsg25D-RF	FBgn0000	3
FBtr03106	314	319	CGATCG	6	...)))	359	Bsg25D-RF	FBgn0000	3
FBtr03106	345	350	CCCGGG	6	...)))	359	Bsg25D-RF	FBgn0000	3
FBtr00813	48	53	CTGCAG	6	.(((((	342	spir-RC	FBgn0003	1
FBtr03444	67	74	TGCCGGCA	8	)...((((	523	sra-RB	FBgn0086	3
FBtr03444	195	200	CAGCTG	6	..))).	523	sra-RB	FBgn0086	3
FBtr03444	282	287	GCTAGC	6	))))))	523	sra-RB	FBgn0086	0
FBtr03073	124	129	GAGCTC	6	((((((	292	CG43367-I	FBgn0263	0
FBtr03073	238	243	GCTAGC	6	((....	292	CG43367-I	FBgn0263	4
FBtr03366	19	24	CCGCGG	6	(...((	545	Dlg5-RC	FBgn0032	3
FBtr03366	93	98	TGGCCA	6	((((..	545	Dlg5-RC	FBgn0032	2
FBtr03366	139	144	GTGCAC	6	((((..	545	Dlg5-RC	FBgn0032	2
FBtr03366	187	194	TTCCGGAA	8	...((((.	545	Dlg5-RC	FBgn0032	4
FBtr03366	194	201	ATCCGGAT	8	....((((	545	Dlg5-RC	FBgn0032	4

FBtr03366	297	302	GAGCTC	6	)))...	545	Dlg5-RC	FBgn0032:	3
FBtr03366	316	321	CGCGCG	6	.))))(	545	Dlg5-RC	FBgn0032:	1
FBtr03366	317	322	GCGCGC	6	))))((	545	Dlg5-RC	FBgn0032:	0
FBtr03366	328	333	CAGCTG	6	((((..	545	Dlg5-RC	FBgn0032:	2
FBtr00819	64	69	CGATCG	6	.....(	548	pum-RB	FBgn0003:	5
FBtr00819	260	265	CAGCTG	6	).((((	548	pum-RB	FBgn0003:	1
FBtr03063	77	84	ACTGCAGT	8	..(((((.	473	larp-RG	FBgn0261(	3
FBtr03063	137	142	CAGCTG	6	..))))	473	larp-RG	FBgn0261(	2
FBtr03063	159	164	CGGCCG	6	.(((((	473	larp-RG	FBgn0261(	1
FBtr03063	184	189	TGGCCA	6	))))).	473	larp-RG	FBgn0261(	1
FBtr03063	227	232	CTGCAG	6	((((..	473	larp-RG	FBgn0261(	2
FBtr03021	282	287	TGGCCA	6	.(((..	711	CG11597-I	FBgn0036:	3
FBtr03021	283	292	GGCCATG(	10	(((....)))	711	CG11597-I	FBgn0036:	4
FBtr03021	449	456	TTGGCCA/	8	((((...(	711	CG11597-I	FBgn0036:	3
FBtr03021	688	693	GACGTC	6	))))))	711	CG11597-I	FBgn0036:	0
FBtr03329	92	97	AGCGCT	6	.(((.(	770	dock-RD	FBgn0010!	2
FBtr03329	265	270	CCTAGG	6	(....)	770	dock-RD	FBgn0010!	4
FBtr03329	448	453	TCCGGA	6	((((.(	770	dock-RD	FBgn0010!	1
FBtr03329	720	727	CCAGCTG(	8	)).)))))	770	dock-RD	FBgn0010!	1
FBtr03329	744	749	CCGCGG	6	).)).)	770	dock-RD	FBgn0010!	2
FBtr03328	330	337	ACGGCCG	8	))))))))	356	CG13933-I	FBgn0035:	0
FBtr00819	255	262	ACAGCTGT	8	).))))).	871	pum-RA	FBgn0003:	2
FBtr00819	559	566	ACGCGCG	8	..((((((	871	pum-RA	FBgn0003:	2
FBtr00819	626	631	CAGCTG	6	)))))(	871	pum-RA	FBgn0003:	0
FBtr00819	659	664	GAGCTC	6	))))))	871	pum-RA	FBgn0003:	0
FBtr03348	150	155	CAGCTG	6	..))).	440	RapGAP1-I	FBgn0264!	3
FBtr03078	533	538	TGGCCA	6	))))).	601	spir-RG	FBgn0003:	1
FBtr00763	289	298	CGCTGCA(	10	...)))).))	473	RasGAP1-I	FBgn0004:	4
FBtr00763	321	330	TAGCCGG(	10	)))).)))((	473	RasGAP1-I	FBgn0004:	1
FBtr00747	60	67	GCTTAAG(	8	(((..(((	416	Tao-RB	FBgn0031(	2
FBtr00747	104	109	TGCGCA	6	)(((((	416	Tao-RB	FBgn0031(	0
FBtr03346	42	47	TGCGCA	6	))))).	683	aqz-RD	FBgn0286!	1
FBtr03346	305	312	ACAGCTGT	8	...))).)	683	aqz-RD	FBgn0286!	4
FBtr03063	29	34	CGATCG	6	((((((	420	larp-RF	FBgn0261(	0
FBtr03063	73	78	CAGCTG	6	..((((	420	larp-RF	FBgn0261(	2
FBtr03063	85	90	CACGTG	6	))))).	420	larp-RF	FBgn0261(	1
FBtr03063	101	112	CACCACGT	12	((((....))))	420	larp-RF	FBgn0261(	4
FBtr03063	189	194	CAGCTG	6	(((((.	420	larp-RF	FBgn0261(	1
FBtr03312	32	43	AACAGCG(	12	(.((.(((...(	553	Pi3K21B-R	FBgn0020(	5
FBtr03312	322	327	TCGCGA	6	..((((	553	Pi3K21B-R	FBgn0020(	2
FBtr03312	376	383	GCTATAG(	8	...)))))	553	Pi3K21B-R	FBgn0020(	3
FBtr03312	382	391	GCGATAT(	10	))..))))))	553	Pi3K21B-R	FBgn0020(	2
FBtr03312	388	393	TCGCGA	6	))))((	553	Pi3K21B-R	FBgn0020(	0
FBtr03312	465	472	GCTGCAG(	8	(((((...	553	Pi3K21B-R	FBgn0020(	3
FBtr01125	73	80	TCCATGG/	8	.....))(	594	pgc-RA	FBgn0016(	5
FBtr01125	127	132	GCTAGC	6	))....	594	pgc-RA	FBgn0016(	4
FBtr01125	225	230	CGCGCG	6	)))))(	594	pgc-RA	FBgn0016(	0

FBtr01125	334	339	TCCGGA	6	..))).	594	pgc-RA	FBgn0016(	3
FBtr01125	415	422	TCCGCGG/	8	(.(.((((	594	pgc-RA	FBgn0016(	2
FBtr01125	486	491	GTGCAC	6	).))))	594	pgc-RA	FBgn0016(	1
FBtr00743	15	20	GCGCGC	6	((((((	696	Septin4-RE	FBgn0259!	0
FBtr00743	16	21	CGCGCG	6	((((((	696	Septin4-RE	FBgn0259!	0
FBtr00743	320	327	GCAGCTG(	8	((((((((	696	Septin4-RE	FBgn0259!	0
FBtr00743	553	558	CAGCTG	6	....))	696	Septin4-RE	FBgn0259!	4
FBtr03039	132	141	CCGAATTC	10	)))..))))..	503	Tao-RF	FBgn0031(	4
FBtr03039	151	156	CTCGAG	6	.(((..	503	Tao-RF	FBgn0031(	3
FBtr03039	187	192	ACGCGT	6	.(..((	503	Tao-RF	FBgn0031(	3
FBtr03039	425	432	CCAGCTG(	8	(.......	503	Tao-RF	FBgn0031(	7
FBtr03039	461	468	GGTCGAC(	8	)(((...(	503	Tao-RF	FBgn0031(	3
FBtr00780	92	97	AGCGCT	6	....))	1184	dock-RA	FBgn0010!	4
FBtr00780	265	270	CCTAGG	6	(....)	1184	dock-RA	FBgn0010!	4
FBtr00780	448	453	TCCGGA	6	((((.(	1184	dock-RA	FBgn0010!	1
FBtr00780	720	727	CCAGCTG(	8	))..))))	1184	dock-RA	FBgn0010!	2
FBtr00780	744	749	CCGCGG	6	).))))	1184	dock-RA	FBgn0010!	1
FBtr00780	796	801	TGCGCA	6	)...))	1184	dock-RA	FBgn0010!	3
FBtr00780	861	866	CAGCTG	6	..))))	1184	dock-RA	FBgn0010!	2
FBtr00780	1046	1051	CTGCAG	6	.....(	1184	dock-RA	FBgn0010!	5
FBtr00780	1085	1090	AGCGCT	6	)).)).	1184	dock-RA	FBgn0010!	2
FBtr00780	1161	1166	GCCGGC	6	)).)).	1184	dock-RA	FBgn0010!	2
FBtr00803	93	98	CGGCCG	6	((....	500	bru1-RB	FBgn0000:	4
FBtr00803	489	494	GGATCC	6	.)))..	500	bru1-RB	FBgn0000:	3
FBtr03100	502	507	GCATGC	6	))))).	776	cbt-RC	FBgn0043:	1
FBtr03100	543	548	CAGCTG	6	((.(((	776	cbt-RC	FBgn0043:	1
FBtr01111	222	227	AGGCCT	6	))))((	382	cta-RA	FBgn0000:	0
FBtr00767	13	18	GGCGCC	6	(((((.	496	pbl-RA	FBgn0003(	1
FBtr00767	71	76	ACGCGT	6	((....	496	pbl-RA	FBgn0003(	4
FBtr00767	324	329	GCGCGC	6	))))))	496	pbl-RA	FBgn0003(	0
FBtr00767	484	489	CTGCAG	6	.)))..	496	pbl-RA	FBgn0003(	3
FBtr00813	533	538	TGGCCA	6	))))).	601	spir-RD	FBgn0003-	1
FBtr03392	533	538	TGGCCA	6	))))).	601	spir-RJ	FBgn0003-	1
FBtr00802	19	24	CCGCGG	6	(...((	481	Dlg5-RA	FBgn0032:	3
FBtr00802	93	98	TGGCCA	6	((((..	481	Dlg5-RA	FBgn0032:	2
FBtr00802	139	144	GTGCAC	6	).((((	481	Dlg5-RA	FBgn0032:	1
FBtr00802	187	194	TTCCGGA/	8	)(((((..	481	Dlg5-RA	FBgn0032:	2
FBtr00802	194	201	ATCCGGAT	8	..))))).	481	Dlg5-RA	FBgn0032:	3
FBtr00802	233	238	GAGCTC	6	(((...	481	Dlg5-RA	FBgn0032:	3
FBtr00802	252	257	CGCGCG	6	).))).	481	Dlg5-RA	FBgn0032:	2
FBtr00802	253	258	GCGCGC	6	.))).)	481	Dlg5-RA	FBgn0032:	2
FBtr00802	264	269	CAGCTG	6	))))))	481	Dlg5-RA	FBgn0032:	0
FBtr03082	40	45	GACGTC	6	((((..	685	CG31998-I	FBgn0051!	2
FBtr03082	252	263	TGCACTAG	12	.(((.(((....	685	CG31998-I	FBgn0051!	6
FBtr03082	573	578	CAGCTG	6	....))	685	CG31998-I	FBgn0051!	4
FBtr00883	59	66	GCGATCG(	8	..((((((	280	Lime-RA	FBgn0033-	2
FBtr00883	101	108	CTGCGCA(	8	((.(((..	280	Lime-RA	FBgn0033-	3

FBtr00883	198	207	TGGCCGG(	10	.((.......	280	Lime-RA	FBgn0033	8
FBtr03319	398	403	GAGCTC	6	))).))	427	bru1-RK	FBgn0000	1
FBtr03402	73	78	CGATCG	6	(....(	319	Tre1-RC	FBgn0046	4
FBtr03402	109	114	AGCGCT	6	(((((.	319	Tre1-RC	FBgn0046	1
FBtr00896	10	15	GTGCAC	6	((((..	1439	larp-RB	FBgn0261	2
FBtr00896	539	546	CAACGTTG	8	(...((((	1439	larp-RB	FBgn0261	3
FBtr00896	862	867	CTGCAG	6	)))).)	1439	larp-RB	FBgn0261	1
FBtr00896	977	982	CGGCCG	6	(.....	1439	larp-RB	FBgn0261	5
FBtr00896	978	985	GGCCGGC(	8	.....)))	1439	larp-RB	FBgn0261	5
FBtr00896	1030	1035	CGCGCG	6	.(((.(	1439	larp-RB	FBgn0261	2
FBtr00896	1099	1104	GCCGGC	6	))))))	1439	larp-RB	FBgn0261	0
FBtr00896	1107	1114	CCGTACG(	8	))))..((	1439	larp-RB	FBgn0261	2
FBtr00896	1198	1203	AGCGCT	6	))))))	1439	larp-RB	FBgn0261	0
FBtr00896	1255	1260	CGCGCG	6	((((..	1439	larp-RB	FBgn0261	2
FBtr00896	1364	1369	GCTAGC	6	))).))	1439	larp-RB	FBgn0261	1
FBtr00896	1394	1399	GCCGGC	6	((((((	1439	larp-RB	FBgn0261	0
FBtr00896	1406	1413	CGCCGGC(	8	))))))))	1439	larp-RB	FBgn0261	0
FBtr00896	1409	1416	CGGCGCC(	8	))))))..	1439	larp-RB	FBgn0261	2
FBtr00815	32	37	TGCGCA	6	(((...	55	del-RA	FBgn0086	3
FBtr00769	67	74	AGACGTCT	8	....)...	247	CG10077-I	FBgn0035	7
FBtr00769	114	119	ACCGGT	6	...)))	247	CG10077-I	FBgn0035	3
FBtr00769	213	218	TGGCCA	6	))).))	247	CG10077-I	FBgn0035	1
FBtr03316	93	98	CGGCCG	6	.))))(	298	bru1-RH	FBgn0000	1
FBtr00719	44	49	CCCGGG	6	....))	208	CycB-RB	FBgn0000	4
FBtr03443	42	49	TCTGCAGA	8	)).((..(	661	eIF4G2-RC	FBgn0260	3
FBtr03443	236	241	GCTAGC	6	(((((.	661	eIF4G2-RC	FBgn0260	1
FBtr03443	288	293	CGTACG	6	...)))	661	eIF4G2-RC	FBgn0260	3
FBtr03443	422	427	TCCGGA	6	))))))	661	eIF4G2-RC	FBgn0260	0
FBtr03443	645	654	CCTTCGAA	10	.))))))..).	661	eIF4G2-RC	FBgn0260	3
FBtr00743	15	20	GCGCGC	6	((((((	363	Septin4-RE	FBgn0259	0
FBtr00743	16	21	CGCGCG	6	((((((	363	Septin4-RE	FBgn0259	0
FBtr00743	220	225	CAGCTG	6	(.((((	363	Septin4-RE	FBgn0259	1
FBtr03392	533	538	TGGCCA	6	))))).	601	spir-RI	FBgn0003	1
FBtr03402	96	101	GAGCTC	6	(...((	861	MESR4-RC	FBgn0034	3
FBtr03402	339	344	GTGCAC	6	((((..	861	MESR4-RC	FBgn0034	2
FBtr03402	436	445	GGGGCGC	10	....))))))	861	MESR4-RC	FBgn0034	4
FBtr03402	588	593	GCGCGC	6	)))).)	861	MESR4-RC	FBgn0034	1
FBtr03402	589	594	CGCGCG	6	))).).	861	MESR4-RC	FBgn0034	2
FBtr03402	763	770	GCAGCTG(	8	)..)))))	861	MESR4-RC	FBgn0034	2
FBtr03402	834	839	GCATGC	6	))..))	861	MESR4-RC	FBgn0034	2
FBtr03011	602	609	CAACGTTG	8	(...((((	1502	larp-RE	FBgn0261	3
FBtr03011	925	930	CTGCAG	6	)))).)	1502	larp-RE	FBgn0261	1
FBtr03011	1040	1045	CGGCCG	6	(.....	1502	larp-RE	FBgn0261	5
FBtr03011	1041	1048	GGCCGGC(	8	.....)))	1502	larp-RE	FBgn0261	5
FBtr03011	1093	1098	CGCGCG	6	.(((.(	1502	larp-RE	FBgn0261	2
FBtr03011	1162	1167	GCCGGC	6	))))))	1502	larp-RE	FBgn0261	0
FBtr03011	1170	1177	CCGTACG(	8	))))..((	1502	larp-RE	FBgn0261	2

FBtr03011	1261	1266	AGCGCT	6	))))))	1502	larp-RE	FBgn0261(	0
FBtr03011	1318	1323	CGCGCG	6	((((..	1502	larp-RE	FBgn0261(	2
FBtr03011	1427	1432	GCTAGC	6	))).))	1502	larp-RE	FBgn0261(	1
FBtr03011	1457	1462	GCCGGC	6	((((((	1502	larp-RE	FBgn0261(	0
FBtr03011	1469	1476	CGCCGGC(	8	))))))))	1502	larp-RE	FBgn0261(	0
FBtr03011	1472	1479	CGGCGCC(	8	)))))))..	1502	larp-RE	FBgn0261(	2
FBtr03429	14	19	GCGCGC	6	(((((.	359	Bsg25D-RI	FBgn0000:	1
FBtr03429	183	190	CACGCGT(	8	...(((((	359	Bsg25D-RI	FBgn0000:	3
FBtr03429	314	319	CGATCG	6	...)))	359	Bsg25D-RI	FBgn0000:	3
FBtr03429	345	350	CCCGGG	6	...)))	359	Bsg25D-RI	FBgn0000:	3
FBtr00829	79	84	TCCGGA	6	...)))	160	spn-B-RA	FBgn0003‹	3
FBtr03330	161	166	TGCGCA	6	))).))	184	del-RC	FBgn0086:	1
FBtr03350	42	47	CGTACG	6	.(.(((	263	nos-RB	FBgn0002!	2
FBtr03350	163	168	TCGCGA	6	(.....	263	nos-RB	FBgn0002!	5
FBtr03029	39	46	CGCATGC(	8	((.(((((	665	Nost-RC	FBgn0259:	1
FBtr03029	334	341	TCAGCTGA	8	(((((..(	665	Nost-RC	FBgn0259:	2
FBtr03029	656	661	TGGCCA	6	.))))..	665	Nost-RC	FBgn0259:	3
FBtr00837	42	47	CGTACG	6	.(.(((	263	nos-RA	FBgn0002!	2
FBtr00837	163	168	TCGCGA	6	(.....	263	nos-RA	FBgn0002!	5
FBtr00819	42	47	TGCGCA	6	))))).	914	aqz-RB	FBgn0286!	1
FBtr00819	188	193	ACGCGT	6	)).(((	914	aqz-RB	FBgn0286!	1
FBtr00819	286	291	TGCGCA	6	(((...	914	aqz-RB	FBgn0286!	3
FBtr00819	370	377	CTGATCAG	8	(((((.((	914	aqz-RB	FBgn0286!	1
FBtr00819	536	543	ACAGCTGT	8	...))).)	914	aqz-RB	FBgn0286!	4
FBtr00747	60	67	GCTTAAG(	8	((..(((.	208	Tao-RA	FBgn0031(	3
FBtr00747	104	109	TGCGCA	6	)).)).	208	Tao-RA	FBgn0031(	2
FBtr03000	8	13	GACGTC	6	((((..	196	Nost-RE	FBgn0259:	2
FBtr03000	17	26	TCGTCGA(	10	.)))))))))	196	Nost-RE	FBgn0259:	1
FBtr03000	64	69	ACGCGT	6	))).((	196	Nost-RE	FBgn0259:	1
FBtr00843	161	166	AGCGCT	6	.(.(((	303	orb-RB	FBgn0004!	2
FBtr00843	296	301	GTGCAC	6	))))..	303	orb-RB	FBgn0004!	2
FBtr00760	276	281	TGGCCA	6	.(((..	354	CG11597-I	FBgn0036:	3
FBtr00760	277	286	GGCCATG(	10	(((....)))	354	CG11597-I	FBgn0036:	4
FBtr00760	331	336	GACGTC	6	))....	354	CG11597-I	FBgn0036:	4
FBtr03073	31	36	TGGCCA	6	.)))))	741	CG43367-I	FBgn0263:	1
FBtr03073	51	56	CTGCAG	6	..((((	741	CG43367-I	FBgn0263:	2
FBtr03073	260	269	GTGCCGG(	10	((..((((..	741	CG43367-I	FBgn0263:	4
FBtr03073	463	474	AGTCAGCT	12	))))))..(((.	741	CG43367-I	FBgn0263:	3
FBtr01130	96	101	GAGCTC	6	(...((	861	MESR4-RB	FBgn0034:	3
FBtr01130	339	344	GTGCAC	6	((((..	861	MESR4-RB	FBgn0034:	2
FBtr01130	436	445	GGGGCGC	10	....))))))	861	MESR4-RB	FBgn0034:	4
FBtr01130	588	593	GCGCGC	6	)))).)	861	MESR4-RB	FBgn0034:	1
FBtr01130	589	594	CGCGCG	6	))).).	861	MESR4-RB	FBgn0034:	2
FBtr01130	763	770	GCAGCTG(	8	)..)))))	861	MESR4-RB	FBgn0034:	2
FBtr01130	834	839	GCATGC	6	))..))	861	MESR4-RB	FBgn0034:	2
FBtr00727	330	337	ACGGCCG	8	))))))))	356	CG13933-I	FBgn0035:	0
FBtr01003	331	336	CTGCAG	6	))))..	434	fabp-RA	FBgn0037!	2

FBtr01003	395	400	AGCGCT	6	))))..	434	fabp-RA	FBgn0037!	2
FBtr00755	157	164	GCAGCTG(	8	))))))).	389	Hip14-RA	FBgn0259!	1
FBtr03073	124	129	GAGCTC	6	))))((	1094	CG43367-I	FBgn0263:	0
FBtr03073	238	243	GCTAGC	6	((((((	1094	CG43367-I	FBgn0263:	0
FBtr03073	384	389	TGGCCA	6	.)))))	1094	CG43367-I	FBgn0263:	1
FBtr03073	404	409	CTGCAG	6	.....)	1094	CG43367-I	FBgn0263:	5
FBtr03073	613	622	GTGCCGG(	10	((..((((..	1094	CG43367-I	FBgn0263:	4
FBtr03073	816	827	AGTCAGCT	12	))))))..(((.	1094	CG43367-I	FBgn0263:	3
FBtr03443	92	97	CAGCTG	6	..))).	348	RhoGAP92	FBgn0038:	3
FBtr03443	273	280	GGTTAAC(	8	.)))..))	348	RhoGAP92	FBgn0038:	3
FBtr00780	92	97	AGCGCT	6	...(((	618	dock-RC	FBgn0010!	3
FBtr00780	265	270	CCTAGG	6	(....)	618	dock-RC	FBgn0010!	4
FBtr00780	448	453	TCCGGA	6	((((.(	618	dock-RC	FBgn0010!	1
FBtr00780	535	542	CTCCGGA(	8	))))((.(	618	dock-RC	FBgn0010!	1
FBtr00780	568	575	CCAGCTG(	8	)..)))))	618	dock-RC	FBgn0010!	2
FBtr00780	592	597	CCGCGG	6	).)).)	618	dock-RC	FBgn0010!	2

trans bp	trans bp score
xxoxxxxox:	1
xxxxxxxxxx	0
xxxxxx	0
xxxxxx	0
xxxxxx	0
xxxxxx	0
xxxxxx	0
xxxxxx	0
xoooox	4
xxxxxx	0
xoooooox	6
xxxxxx	0
xxxxxx	0
xxxxxxxxxx	0
xxxxxx	0
xxxooxxoo	2
xxxxxx	0
xxxxxx	0
xxxxxx	0
xxoxxoxx	1
xxxooxxx	2
xxxxxx	0
xxxxxx	0
xxxxxx	0
xxxxxx	0
xxxxxx	0
xxxxxxxx	0
xxxxxxxx	0
xxxxxx	0
xoxxox	1
xxxxxxxx	0
xxxxxxxx	0
xxxxxx	0
xxxxxx	0
xxxxxx	0
xxxxxxxx	0
xxxxxxxx	0
xxxxxx	0
xxxxxx	0
xxxxxxxx	0
xxxxxx	0
xxxxxxxx	0
oxxxxo	1
xxxxxx	0
xxxxxx	0
xxxxxx	0

xxxxxxxx	0
xxxxxx	0
xxxxxx	0
xxxxxx	0
xxooxx	2
xxxooxxx	2
xxxxxx	0
xxoxxxxoxx	1
xxxxxxxxxx	0
xxxxxxxx	0
xxxxxx	0
oxxxxo	1
xxxxxxxx	0
xxooxx	2
xoooooox	6
xxxxxxxx	0
xxxxxx	0
xxxxxx	0
oxxxxxxo	1
xxxxxx	0
xoxxox	1
xxooooxx	4
xxxxxx	0
xxxxxx	0
xxxxxx	0
xxxxxx	0
xxxxxxxx	0
xxxxxxxx	0
xxooooxx	4
xxxxxx	0
xxxxxx	0
xxxxxxxx	0
xxxxxx	0
xxxxxx	0
oxxxxo	1
xxxxxxxx	0
xxooxx	2
xoooooox	6
xxooxx	2
xxxxxx	0
xxxxxx	0
oxxxxxxo	1
xxxxxxxx	0
xxxxxx	0
xxxxxx	0
xxxxxx	0
xxxxxx	0

xxxxxx	0
xoooox	4
xxxxxx	0
xxooxx	2
xxxxxx	0
xxxxxx	0
oxxxxxxo	1
xxxxxxxx	0
xxxxxx	0
xxxxxx	0
xxxxxx	0
xxxxxx	0
xxxxxxxxxx	0
oxxxxo	1
xxooxx	2
xoooooox	6
xxxxxxxx	0
xxxxxx	0
xxxxxxxx	0
xxxxxxxx	0
xxxxxx	0
xxxxxx	0
xxxxxx	0
xxxxoooox	4
xxxxxx	0
xxxxxx	0
xxooxx	2
xxooxx	2
xxxxxxxx	0
xxxxxx	0
xxxxxx	0
xxxxxx	0
xxxxxx	0
xxxxxx	0
xxxxxx	0
xxxxxx	0
oxxxxo	1
xoooox	4
xxxxxx	0
xxxxxxxx	0
xoxxox	1
xxxxxx	0
xxxxxx	0
oxxxxo	1
xxxxxxxx	0
xxooxx	2
xoooooox	6

xxxxxxxxxxx	0
xxxxxx	0
xxooxx	2
xxxooxxx	2
xxxxxx	0
xxxxxx	0
xxxxxx	0
xxxxxxxx	0
xoxxxxox	1
oxxxxo	1
xxxxxx	0
xoooooox	6
xxxxxxxx	0
xxxxxx	0
xxxxxx	0
xxxxxx	0
xxxxxx	0
xxxxxx	0
xxxxxx	0
xxxxxx	0
xxxxxx	0
xxxxxxxx	0
xxxxxx	0
xxxxxx	0
xxxxxx	0
xoooox	4
xxxxxx	0
xxxooooxx	4
xxxxxx	0
xxxxxx	0
xxxxxxxx	0
xxxxxx	0
xxxxxx	0
xxxxxxxx	0
xxxxxx	0
xxxxxx	0
xxxxxx	0
xxxxxx	0
xxxxxx	0
xxxxxx	0
xoxxxxox	1
xxxxxx	0
xxxxxx	0
xxxxxxxx	0
xxxxxx	0
xxxxxxxx	0
xxxxxxxx	0

oxxxxo	1
xxxxxx	0
xxxxxxxxxx	0
xxxxxx	0
xoxxxxxxxo	1
xxxxxx	0
xxxxxxxxxx	0
xxxxxx	0
xxxxxx	0
xxxxxx	0
xxxxxx	0
xxxxxxxxxx	0
xxxxxx	0
xxxxxx	0
xxooxx	2
xxxxxx	0
oxxxxo	1
xxxxxx	0
xxxxxxxx	0
xxxxxx	0
xxxxxx	0
xxxxxx	0
xxxxxx	0
xxxxxx	0
xoooox	4
xoxxox	1
xxxxxx	0
xxxxxx	0
xxxxxxxx	0
xxxxxx	0
xxxxxx	0
oxxxooxxx	2
oxxxxo	1
xxooxx	2
xoooooox	6
xoxxxxox	1
xxxxxx	0
oxxxxo	1
xxxxxx	0
xxxxxxxx	0
xxxxxxxx	0
xxxxxxxx	0
xxxxxxxx	0
xxxxxxxx	0
oxxxxo	1
xxxxxx	0
xxxxxx	0

xxxxxx	0
xxxxxxxx	0
xxxxxx	0
xoxxox	1
xxxxxx	0
xxxxxxxx	0
xxooxx	2
xoooooox	6
xxxxxxxx	0
xxxxxx	0
xxxxxx	0
xxxxxx	0
oxxxxxxo	1
xxxxxx	0
xxxxxx	0
xxxxxx	0
xxxxxx	0
xxxxxx	0
xoxxox	1
xoxxxxxxxx	1
xxxxxx	0
xxxxxxxx	0
xxxxxxxxxx	0
xxxxxx	0
xxxxxxxx	0
xxooxx	2
xxxxxx	0
xxxxxxxx	0
xxxxxx	0
xxxxxxxxxx	0
oxxxxo	1
xxooxx	2
xoooooox	6
xxxxxxxx	0
oxxxxo	1
xxxooooxx	4
xxxxxxxx	0
xxxxxx	0
xxooxx	2
xxxxxx	0
xxxxxx	0
xxooxx	2
xxxxxxxx	0
xoxxxxox	1
oxxxxo	1
xxxooooxx	4
xxoxxxxoxx	1

xxxxxx	0
xxxxxxxxxx	0
xxxxxx	0
xoxxxxxxox	1
xxxxxx	0
xxxxxxxxxx	0
xxxxxx	0
xxooxx	2
xxxxxx	0
xxooxx	2
xxooxx	2
xxxxxxxx	0
xxxxxx	0
xxxxxx	0
xxxxxx	0
xxxxxxxx	0
xxxxxx	0
xxxxxx	0
xxxxxxxxxx	0
xxxxxx	0
xxxxxx	0
xxxxxx	0
oxxxxo	1
xxxxxx	0
xxxxxxxx	0
xxxxoooox	4
xxxxxx	0
xxxxxx	0
xxxxxx	0
xxxxxx	0
xxxxxx	0
xxxxxx	0
xxxxxx	0
xxxxxxxx	0
xxxxxx	0
xxxxxx	0
xxxxxx	0
xxxxxxxx	0
oxxxxo	1
xxxxxx	0
xxxxxx	0
xxooxx	2
xxooxx	2
xxxxxx	0
xxxxxx	0
oxxxxxxo	1
xxxxxxxx	0

xxxxxx	0
xxxxxx	0
xxxxxx	0
xxxxxx	0
xoooox	4
xxxxxx	0
oxxxxxxo	1
xxxxxx	0
xxxxxx	0
xxxxxx	0
xxxxxx	0
oxxxxo	1
xxxooooxx	4
xxxxxxxx	0
xxxxxx	0
xxxxxx	0
xoooox	4
xxxxxx	0
xxxxxxxx	0
xoxxox	1
xxxxxxxx	0
xxxxxxxx	0
xxxxxxxx	0
xxxxxx	0
xxxxxx	0
oxxxxo	1
xxxxxx	0
xxoxxxxoxx	1
xxxxxxxxxx	0
xxxooxxx	2
xxxxxx	0
xxxxxx	0
xoxxxxox	1
xxxxxx	0
xxxxxx	0
xxxxxx	0
xxxxoooox	4
xxxxxx	0
xoxxxxxxxx	1
xxxxxx	0
xxxxxxxx	0
xxxxxxxxxx	0
xxxxxx	0
xxxxxxxx	0
xxxooxxx	2
xxooxx	2
xxxxxx	0

oxxxxo	1
xxxxxxxx	0
xxxxxx	0
xxxxxx	0
xxxxxx	0
xxxxxxxx	0
xxooxx	2
xxxxxxxxxx	0
oxxxxo	1
xxooxx	2
xoooooox	6
xxxxxxxx	0
xxooxx	2
xoooox	4
xxxxxx	0
xxxxxxxx	0
xxxxxx	0
xxooxx	2
xxxxxx	0
xoooox	4
xxxxxx	0
xxxxxx	0
xxooxx	2
oxxxxo	1
xxxxxx	0
xxxxxx	0
xxxxxx	0
xxxxxx	0
xxooxx	2
xxxxxx	0
oxxxxo	1
xxxxxx	0
xxxxxx	0
xxooxx	2
xxxxxx	0
xxxxxx	0
xxxxxxxx	0
oxxxxxxo	1
xxxxxx	0
xxxxxx	0
xxxxxx	0
xxxxxx	0
xxxxxx	0
oxxxxxxxxx	1
xxooxx	2
xxxxxxxx	0
xxxxxxxx	0

oxxooooxx	4
xxxxxx	0
xoooox	4
xxxxxx	0
xxxxxx	0
xxxxxxxx	0
xxxxxx	0
xoooox	4
xxxooxxx	2
xxxxxx	0
xxxxxx	0
xxxxxxxx	0
xxxxxx	0
xxxxxx	0
xxxxxx	0
xxxxxx	0
xxxxxxxx	0
xxxxxxxx	0
xxxxxx	0
oooxxooo	3
xxxxxx	0
xxxxxx	0
xxxxxx	0
xxooxx	2
xxoxxoxx	1
xxxxxx	0
xxxxxx	0
xxxxxx	0
oxxxxxxxxo	1
xxxxxx	0
xxxxxx	0
xxxxxx	0
xxxxxx	0
xxooxx	2
xxxxxx	0
xxxxxxxxxx	0
xxxxxx	0
xxxxxx	0
xxxxxxxx	0
xxooxx	2
xxxxxxxx	0
xxxxxx	0
xoooox	4
xxxooxxx	2
xxxxxx	0
xxxxxx	0
xxxxxxxx	0

xxxxxx	0
xxxxxx	0
xxxxxx	0
xxxxxx	0
xxxxxxxx	0
xxxxxxxx	0
xxxxxx	0
xxxxxxxx	0
xxxxxx	0
xxxxxx	0
xxxxxx	0
xxxxxx	0
xxxxxx	0
xoooox	4
xxxxxxxx	0
xxxxxxxx	0
oxxxxo	1
xxxxxx	0
xoooox	4
xxxxxx	0
xxxxxx	0
xxxxxx	0
xxxxxxxx	0
xoxxxxox	1
xxxxxxxx	0
xxxxxx	0
xxxxxx	0
xxxxxxxxxx	0
xxxxxx	0
xxxxxx	0
xxxxxx	0
oxxxxo	1
xxxooooxx	4
xxooxx	2
xxxxxx	0
xxxxxx	0
xxxxxxxxxx	0
xxxxxxxxxx	0
xxooxx	2
xxxxxx	0
xxxxxxxxxx	0
xxxxxx	0
xxxxxx	0
xxxxxxxx	0
xxooxx	2
xxxxxxxx	0
xxxxxx	0

xxxxxx	0
xxxxxxxx	0
xxxxxx	0
xxxxxx	0
xxxxxx	0
xoooox	4
xxxxxxxxxxx	0
xxxxxxxxxxx	0
oxxxxo	1
xxxxxxxx	0
xxxxxx	0
xoooox	4
xxxxxx	0
xxxxxxxx	0
xxxxxxxx	0
xoxxox	1

# genes: **13**
# genes and isoforms: **35**

5UTR

Gene name	RPKM (early embryo)	Embryo stage
osk	362	0-2
CG3071	83	0-2
santa-maria	<1	0-2
Dtg	8	0-2
CG31145	1	0-2
Syx1A	10	0-2
Cam	97	0-2
GalT1	27	0-2
Drip	<1	0-2
bun	34	0-2
CoRest	58	0-2
Acn	56	0-2
CG4612	40	0-2
CG12177	9	0-2

RPKM (highest expression during developmental)	Stage exhibiting the highest expression
83	0-2 embryo
51	pupae
254	older embryo
25	pupae
30	22-24 embryo
162	older embryo
27	0-2 embryo
30	larvae
53	pupae
63	older embryo
124	older embryo
42	pupae
68	older embryo

Stau target in stage 0-2 embryo (*)
Yes
No
No
No
No
No
No
No
No
No
No
No
No
No

(*) Laver, J. D. et al. Genome-wide analysis of Staufen-associated mRNAs identifies secondary structure

s that confer target specificity. Nucleic Acids Res 41, 9438-9460, doi:10.1093/nar/gkt702 (2013).

# genes: **38**

# genes and isoforms: **101**

		CDS
Gene name	RPKM (early embryo)	Embryo stage
osk	362	0-2
CG3703	1	0-2
Pc	35	0-2
CG9804	1	0-2
eIF3a	220	0-2
tkv	34	0-2
CG10631	13	0-2
alpha-Est3	<1	0-2
MRG15	55	0-2
GATAe	<1	0-2
RhoGEF2	35	0-2
ND-75	106	0-2
shot	5	0-2
TppII	131	0-2
CG8858	21	0-2
gammaTry	<1	0-2
CAP	<1	0-2
ND-49	60	0-2
CG15117	<1	0-2
CG34408	21	0-2
didum	36	0-2
SdhA	106	0-2
CG43394	1	0-2
px	18	0-2
MED14	33	0-2
l(3)72Ab	37	0-2
Camta	8	0-2
Ccz1	7	0-2
CG15643	16	0-2
dome	103	0-2
pip	<1	0-2
Pep	243	0-2
CG10516	12	0-2
upSET	38	0-2
scyl	94	0-2
Rbfox1	5	0-2
msl-3	55	0-2
Cbs	97	0-2
l(1)G0004	67	0-2

RPKM (highest expression during developmental)	Stage exhibiting the highest expression
10	older embryo
35	0-2 embryo
3	older embryo
344	older embryo
86	older embryo
13	0-2 embryo
4	adult male
82	older embryo
59	older embryo
37	larvae
448	adult male
67	older embryo
131	0-2 embryo
21	0-2 embryo
4	larvae
49	older embryo
188	older embryo
36	older embryo
28	older embryo
49	adult male
464	adult male
28	older embryo
24	older embryo
42	older embryo
104	older embryo
8	0-2 embryo
22	older embryo
16	0-2 embryo
179	older embryo
28	older embryo
684	older embryo
141	pupae
38	0-2 embryo
168	pupae
21	older embryo
55	0-2 embryo
133	larvae
96	larvae

Stau target in stage 0-2 embryo (*)
Yes
No
Yes
No
No
No
No
No
No
No
No
No
No
No
No
No
No
No
No
No
No
No
No
No
Yes
No
No
No
No
No
No
No
No
No
No
No
No
No
No

(*) Laver, J. D. et al. Genome-wide analysis of Staufen-associated mRNAs identifies secondary structure

s that confer target specificity. Nucleic Acids Res 41, 9438-9460, doi:10.1093/nar/gkt702 (2013).

# genes: 9
# genes and isoforms: 19

3UTR

Gene name	RPKM (early embryo)	Embryo stage
osk	362	0-2
shv	51	0-2
α-Est3	<1	0-2
MetRS-m	11	0-2
CG16953	61	0-2
CG11076	84	0-2
B52	116	0-2
CG42724	16	0-2
Ank2	<1	0-2
CG7546	55	0-2

RPKM (highest expression during developmental)	Stage exhibiting the highest expression
80	older embryo
4	adult male
16	older embryo
90	larvae
105	older embryo
302	older embryo
52	older embryo
10	older embryo
68	older embryo

Stau target in stage 0-2 embryo (*)
Yes
No
No
No
No
No
No
No
No
No

(*) Laver, J. D. et al. Genome-wide analysis of Staufen-associated mRNAs identifies secondary structure

s that confer target specificity. Nucleic Acids Res 41, 9438-9460, doi:10.1093/nar/gkt702 (2013).

# genes: 9
# genes and isoforms: 19

3UTR

Gene name	RPKM (early embryo)	Embryo stage
osk	362	0-2
shv	51	0-2
α-Est3	<1	0-2
MetRS-m	11	0-2
CG16953	61	0-2
CG11076	84	0-2
B52	116	0-2
CG42724	16	0-2
Ank2	<1	0-2
CG7546	55	0-2

RPKM (highest expression during developmental)	Stage exhibiting the highest expression
80	older embryo
4	adult male
16	older embryo
90	larvae
105	older embryo
302	older embryo
52	older embryo
10	older embryo
68	older embryo

Stau target in stage 0-2 embryo (*)
Yes
No
No
No
No
No
No
No
No
No

(*) Laver, J. D. et al. Genome-wide analysis of Staufen-associated mRNAs identifies secondary structure

s that confer target specificity. Nucleic Acids Res 41, 9438-9460, doi:10.1093/nar/gkt702 (2013).

# genes: **13**
# genes and isoforms: **35**

5UTR

Gene name	RPKM (early embryo)	Embryo stage
osk	362	0-2
CG3071	83	0-2
santa-maria	<1	0-2
Dtg	8	0-2
CG31145	1	0-2
Syx1A	10	0-2
Cam	97	0-2
GalT1	27	0-2
Drip	<1	0-2
bun	34	0-2
CoRest	58	0-2
Acn	56	0-2
CG4612	40	0-2
CG12177	9	0-2

RPKM (highest expression during developmental)	Stage exhibiting the highest expression
83	0-2 embryo
51	pupae
254	older embryo
25	pupae
30	22-24 embryo
162	older embryo
27	0-2 embryo
30	larvae
53	pupae
63	older embryo
124	older embryo
42	pupae
68	older embryo

Stau target in stage 0-2 embryo (*)
Yes
No
No
No
No
No
No
No
No
No
No
No
No
No

(*) Laver, J. D. et al. Genome-wide analysis of Staufen-associated mRNAs identifies secondary structure

s that confer target specificity. Nucleic Acids Res 41, 9438-9460, doi:10.1093/nar/gkt702 (2013).

# genes: **38**

# genes and isoforms: **101**

		CDS
Gene name	RPKM (early embryo)	Embryo stage
osk	362	0-2
CG3703	1	0-2
Pc	35	0-2
CG9804	1	0-2
eIF3a	220	0-2
tkv	34	0-2
CG10631	13	0-2
alpha-Est3	<1	0-2
MRG15	55	0-2
GATAe	<1	0-2
RhoGEF2	35	0-2
ND-75	106	0-2
shot	5	0-2
TppII	131	0-2
CG8858	21	0-2
gammaTry	<1	0-2
CAP	<1	0-2
ND-49	60	0-2
CG15117	<1	0-2
CG34408	21	0-2
didum	36	0-2
SdhA	106	0-2
CG43394	1	0-2
px	18	0-2
MED14	33	0-2
l(3)72Ab	37	0-2
Camta	8	0-2
Ccz1	7	0-2
CG15643	16	0-2
dome	103	0-2
pip	<1	0-2
Pep	243	0-2
CG10516	12	0-2
upSET	38	0-2
scyl	94	0-2
Rbfox1	5	0-2
msl-3	55	0-2
Cbs	97	0-2
l(1)G0004	67	0-2

RPKM (highest expression during developmental)	Stage exhibiting the highest expression
10	older embryo
35	0-2 embryo
3	older embryo
344	older embryo
86	older embryo
13	0-2 embryo
4	adult male
82	older embryo
59	older embryo
37	larvae
448	adult male
67	older embryo
131	0-2 embryo
21	0-2 embryo
4	larvae
49	older embryo
188	older embryo
36	older embryo
28	older embryo
49	adult male
464	adult male
28	older embryo
24	older embryo
42	older embryo
104	older embryo
8	0-2 embryo
22	older embryo
16	0-2 embryo
179	older embryo
28	older embryo
684	older embryo
141	pupae
38	0-2 embryo
168	pupae
21	older embryo
55	0-2 embryo
133	larvae
96	larvae

Stau target in stage 0-2 embryo (*)
Yes
No
Yes
No
No
No
No
No
No
No
No
No
No
No
No
No
No
No
No
No
No
No
No
No
Yes
No
No
No
No
No
No
No
No
No
No
No
No
No
No

(*) Laver, J. D. et al. Genome-wide analysis of Staufen-associated mRNAs identifies secondary structure

s that confer target specificity. Nucleic Acids Res 41, 9438-9460, doi:10.1093/nar/gkt702 (2013).

osk Fw	CCGGAACCGACAAAATAAGA
osk Rv	ATGGCATTGGCAAAACAAAT
stau Fw	AGAAGCCGGAGACTAGACAGG
stau Rv	ATTGGGGTCTTGTCCTTGCC
Gapdh2 Fw	CCGATGAGGAGGTCGTTTCTA
Gapdh2 Rv	GTGGGTAGTGTTCTTGGTGTTC
eGFP Fw	GCAAGCTGACCCTGAAGTTC
eGFP Rev	GCATGGCGGACTTGAAGAAG
Act5c Fw	AAGTACCCCATTGAGCACGG
Act5C Rev	ACATACATGGCGGGTGTGTT

SUPPLEMENTAL FIGURE 1: Characterizing mRNA clustering of *osk* in embryos using smFISH. **A** Fitting the distribution of *osk* fluorescent puncta to a two-peak (black) and a single-peak (orange) Gaussian curve for WT embryos. Significant deviation of the fit from the data is observed for the single-peak Gaussian fit (see also Supplemental Table 1). **B** mRNA expression levels of *osk* mRNA determined for WT, WT late, RNAi/*matα*, RNAi/*nosp Gal4*, *osk*^{54}*/Df* and *osk*^{54}/+ embryos using qRT-PCR analysis. Mean±STDEV mRNA levels from three biological replicates are shown. *osk* mRNA levels were normalized to *GAPDH2*. Statistical significance: two-tailed *t*-test. *: P = 0.01, **: P = 0.008 and ****: P < 0.001. n.s.: not significant. 3**C** Representative smFISH images of *osk* mRNA at the posterior pole of WT and *stau*^{1}*/stau*^{2} emb3ryos. 20 embryos per genotype were analyzed. **D** Egg hatching rates for *stau*^{1}*/stau*^{2} embryos and their siblings (*stau*^{1}*/cyo* or *stau*^{1}*/cyo*) derived from the same cross and *stau*^{1}*/stau*^{2}*;osk*$^{+/-}$ embryos laid by *stau*^{1}*/stau*^{2}*;oskDef/*+ females. Data represent a mean±STDEV of three biological replicates. Between 123 and 792 sibling, 214 and 414 *stau*^{1}*/stau*^{2}, and 268 and 524 *stau*^{1}*/stau*^{2}*;osk*$^{+/-}$ embryos were laid per apple juice plate. Statistical significance: two-tailed *t*-test. ****: P < 0.001. **E** Validation of the smFISH quantification. *osk* mRNA concentration (in nM) measured by smFISH was extrapolated from a standard curve using its respective expression levels determined by RNA-sequencing data obtained from Flybase(mod *et al.*, 2010). The standard curve was generated by correlating concentrations determined by smFISH and sequencing of six reference mRNAs (*gcl*, *tao1*, *ccr4*, *sra*, *pum* and *eIF4G2*; dashed line R^2=0.72). Green circle: somatic concentration of 1.59 nM for *osk* mRNA estimated from the standard curve. Magenta circle and error bars: somatic concentration of 1.43±0.2 nM for *osk* measured by smFISH. **Fi,ii** Image of *osk* mRNA clusters in WT embryos (i; taken from Figure 1Eii). Yellow dashed lines depict lines scans used to determine the dimensions and linear fluorescence intensity profiles (ii) of *osk* mRNA clusters of different sizes. Mean±STDEV of 20 lines scans for each cluster size are plotted. **G** Mean±STDEV of 20 lines scans for ccr4 monomers (blue) and dimers (gray). **H** Mean±STDEV of 20 lines scans for 100 nm TetraSpeck microspheres.

SUPPLEMENTAL FIGURE 2: Concentration-driven enhancement of *osk* clustering across different genetic backgrounds. **A** Average *osk* cluster size plotted against the corresponding *osk* mRNA concentration recorded for six different genotypes and developmental stages (Materials and Methods). For comparison, the average (magenta line) ± STDEV (orange line) cluster size of

12 reference mRNAs (*aret, ccr4*, *CG18446*, *eIF4G2*, *gcl*, *orb, Pi3K*, *p53*, *pum*, *shutdown*, *sra* and *tao1*) and their somatic concentrations characterized previously(Trcek *et al.*, 2020) are shown. **B** Distribution of *osk* mRNA across clusters of varying size. For each cluster size, mean±STDEV from seven WT, WT late, RNAi/*nosp Gal4*, osk^{54}/Df and $osk^{54}/+$ and three RNAi/*matα* embryos is shown.

SUPPLEMENTAL FIGURE 3: *osk* clustering is diminished in the absence of Staufen expression. **A** Dependence of average *osk* mRNA cluster size on its concentration (nM). Data represent mean±STDEV from seven WT, $stau^1/stau^2$ and $stau^1/stau^2;osk^{+/-}$ embryos. Data for WT and $stau^1/stau^2;osk^{+/-}$ are from Figure 2F. Statistical significance: two-tailed *t*-test. *: P = 0.01, **: P = 0.002, ****: P < 0.001. n.s.: not significant. **B** *osk* mRNA expression levels determined for WT, $stau^1/stau^2$ and $stau^1/stau^2;osk^{+/-}$ embryos using qRT-PCR analysis. Mean±STDEV mRNA levels from three biological replicates are shown. *osk* mRNA levels were normalized to *GAPDH2*. Statistical significance: two-tailed *t*-test. *: P = 0.04, **: P = 0.003. **C** *stau* mRNA expression levels determined for WT embryos and those overexpressing *stau* using qRT-PCR analysis. Mean±STDEV mRNA levels from three biological replicates are shown. *stau* mRNA levels were normalized to *GAPDH2*. Statistical significance: two-tailed *t*-test. **: P = 0.007, ***: P = 0.002. n.s.: not significant. **D** Maximal projection of five consecutive optical sections of an ROI, spaced 150 nm apart of *osk* mRNA in $stau^1/stau^2$ embryos (i) with a corresponding heat map. **E** Distribution of *osk* mRNA across clusters of varying size. For each cluster category, mean±STDEV of seven embryos per genotype is shown. Data for WT is from Figure 2G. In blue, the mean±STDEV molarity (n = seven) of *osk* mRNA monomers for each genotype is shown.

SUPPLEMENTAL FIGURE 4: Evaluating expression and localization of eGFP transgenes. **A** Crossing scheme that generated females that expressed *eGFP-WT* or *eGFP-AA* but lacked endogenous *osk* mRNA expression. osk^{A87} is an RNA null allele of the *osk* gene(Jenny *et al.*, 2006), while $Df(3R)p^{XT103}$ is an *osk* gene deficiency(Ephrussi *et al.*, 1991). **B** The UAS-Gal4/*nanos* promoter system did not drive sufficient expression of *eGFP-WT* and *eGFP-AA* transgenes in $osk^{A87}/Df(3R)p^{XT103}$ females, which lack endogenous *osk* RNA expression, to restore egg laying. Per genotype, 21 three-to-four-day old females were crossed with 10 WT males an allowed to lay eggs on an apple juice plates for 24h (Materials and Methods). Mean±STDEV of six, five, three

and four apple juice plates for *eGFP-WT*, *WT-SIB*, *eGFP-AA* and *AA-SIB* females generated in A is shown. Between 0 and 21 eggs were laid per apple juice plate by *eGFP-WT* and *eGFP-AA* females and 176 to 856 eggs were laid per apple juice plate by *WT-SIB* and *AA-SIB* females. Statistical significance: two-tailed *t*-test. **: P = 0.007 and 0.006 for *eGFP-WT* and *WT-SIB* and *eGFP-AA* and *AA-SIB* comparison, respectively. **C,D** mRNA expression levels of *eGFP-WT* and *eGFP-AA* (C) and of *eGFP-WT* and *eGFP-AA* along with the endogenous *osk* mRNA (D). Data represent mean±STDEV mRNA levels from three biological replicates. mRNA levels were normalized to *GAPDH2*. Statistical significance: two-tailed *t*-test. n.s.: not significant. **E,F** Representative smFISH images of embryos expressing endogenous *osk* mRNA (Ei,Fi) and of *eGFP-WT* (Eii) and *eGFP-AA* mRNA (Fii). **G,H** Fold enrichment of *eGFP-WT* and *eGFP-AA* mRNA at the posterior pole (G). Data represent mean±STDEV of 10 (*eGFP-WT*) and 9 (*eGFP-AA*) embryos. Statistical significance: two-tailed *t*-test. ****: P < 0.001. Fold enrichment was calculated by determining the ratio of eGFP fluorescent levels, normalized by the area between the posterior (magenta ROI) and outside (green ROI, Material and Methods) (H). **I** Distribution of *eGFP-WT* and *eGFP-AA* mRNA across clusters of varying sizes. Clusters that co-localized with the endogenous osk were removed from the analysis. Bars represent mean±STDEV cluster size from 13 *eGFP-WT* and 12 *eGFP-AA* embryos. In blue, the mean±STDEV molarity of *eGFP-WT* and *eGFP-AA* mRNA monomers (determined from 13 and 12 embryos, respectively) for each genotype is shown.

SUPPLEMENTAL FIGURE 5: Evaluating co-localization between endogenous *osk* and *eGFP-WT* and *eGFP-AA* mRNA using spot detection analysis. A,B Representative smFISH images of co-localizing endogenous *osk* (magenta) and *eGFP-WT* mRNA (A) and *eGFP-AA* mRNA (B) (green). White circles show co-localized *osk* and *eGFP-WT* mRNA or *eGFP-AA* mRNAs at the 250nm and 100 nm cutoff. Scale bar: 10 µm. **C,D** Percent of *eGFP-WT* mRNA and *eGFP-AA* mRNA identified as true monomers, dimers and oligomers using a 250 nm (C) and 100 nm (D) co-localization cut-off. Bars represent mean±STDEV cluster size from 13 *eGFP-WT* and 12 *eGFP-AA* embryos. Pie charts show the composition of each *eGFP* cluster category based on its association with endogenous *osk* mRNA. Differences in heterotypic cluster size between *eGFP-WT* and *eGFP-AA* were evaluated using a two-tailed *t*-test.

SUPPLEMENTAL FIGURE 6: Search for exposed GC-rich palindromes through the early *Drosophila* transcriptome. A Scatter plot of the total number of palindromes identified within transcript expressed in the early *Drosophila* embryo as a function of transcript length. **B** Box plot of the palindrome density of ubiquitous and posterior localized *Drosophila* early embryo transcripts. 20 transcripts are selected randomly from each group and overlaid onto the box plot.

Supplemental_Table_1: The average fluorescence intensity of a single mRNAs hybridized with smFISH probes derived from fitting the distribution of fluorescent puncta to a Gaussian curve.

Supplemental_Table_2: List of all ≥ 6 nt long palindromes in the early *Drosophila* transcriptome.

Supplemental_Table_3: List of candidate mRNAs that may contain ≥ 6 nt long, exposed GC-rich palindromes in the early *Drosophila* transcriptome.

Supplemental_Table_4: List of qRT-PCR primers used in this study.
