# Supplementary material for: In vivo clustering of oskar mRNA is driven by RNA concentration, RNA binding proteins, and an RNA palindrome
Source: Mol Biol Cell. 2026 Aug 12;37(10):ar92. doi: 10.1091/mbc.E26-04-0176 (PMC13549266; doi:10.1091/mbc.E26-04-0176)
Supplement: Supplementary file 2 [file reviewer_reportE26-04-0176.pdf]

In vivo clustering of oskar mRNA is driven by RNA concentration, RNA binding proteins, and an RNA palindrome

Ziqing Ye, Siran Tian, Ayse Ecer, and Tatjana Trcek

Corresponding author(s): Tatjana Trcek, Johns Hopkins University

Review Timeline:		
	Submission Date:	2026-04-19
	Editorial Decision:	2026-06-02
	Revision Received:	2026-07-08
	Editorial Decision:	2026-07-22
	Revision Received:	2026-08-03
	Accepted:	2026-08-04

Editor-in-Chief: John Aitchison

Transaction Report:

(Note: With the exception of the correction of typographical or spelling errors that could be a source of ambiguity, letters and reports are not edited. The original formatting of letters and referee reports may not be reflected in this compilation.)

RE: Manuscript #E26-04-0176
TITLE: Synergy of RNA Concentration, RNA Binding Proteins, and RNA Palindrome Drives clustering of oskar mRNA in vivo

Dear Dr. Trcek:

As you can see from the reviewer comments, both reviewers find the work to be carefully executed and they see the work as advancing the field.

However, there is some concern that some of the conclusions may be stronger than supported by the specific data presented. The authors could address this concern by tempering some of their conclusions or mentioning potential caveats or other interpretations of the results. One of the reviewers specifically states that the authors should "discuss false positive rates and whether any of these palindromes overlap known structured elements." Three are also some minor points and definitions that should be addressed.

Sincerely,

Anita Corbett, PhD
Monitoring Editor
Molecular Biology of the Cell

Dear Dr. Trcek,

The review of your manuscript, referenced above, is now complete. The Monitoring Editor has decided that your manuscript is not acceptable for publication at this time, but may be deemed acceptable after specific revisions are made, as described in the Monitoring Editor's decision letter above and the reviewer comments below.

A reminder: Please do not contact the Monitoring Editor directly regarding your manuscript. If you have any questions regarding the review process or the decision, please contact the MBoC Editorial Office (mboc@ascb.org).

When submitting your revision include a rebuttal letter that details, point-by-point, how the Monitoring Editor's and reviewers' comments have been addressed. (The file type for this letter must be "rebuttal letter"; do not include your response to the Monitoring Editor and reviewers in a "cover letter.") Please bear in mind that your rebuttal letter will be published with your paper if it is accepted, unless you have opted out of publishing the review history.

Authors are allowed 180 days to submit a revision. If this time period is inadequate, please contact us at mboc@ascb.org.

Revised manuscripts are assigned to the original Monitoring Editor whenever possible. However, special circumstances may preclude this. Also, revised manuscripts are often sent out for re-review, usually to the original reviewers when possible. The Monitoring Editor may solicit additional reviews if it is deemed necessary to render a completely informed decision.

In preparing your revised manuscript, please follow the instruction in the Information for Authors (www.molbiolcell.org/info-for-authors). In particular, to prepare for the possible acceptance of your revised manuscript, submit final, publication-quality figures with your revision as described.

To submit the rebuttal letter, revised manuscript, and figures, use this link: Link Not Available

MBoC offers the option to publish your work with immediate open access. Open access can increase the discoverability and usability of your research. If you would like to publish your paper with immediate open access but did not select this option during initial submission, please contact the editorial office at mbc@ascb.org.

Authors of Articles and Brief Communications are also encouraged to create a short video abstract to accompany their article when it is published. These video abstracts, known as Science Sketches, are up to 2 minutes long and will be published on YouTube and then embedded in the article abstract. Science Sketch Editors on the MBoC Editorial Board will guide you as you prepare your video. Information about how to prepare and submit a video abstract is available at www.molbiolcell.org/science-sketches. Please contact mboc@ascb.org if you are interested in creating a Science Sketch.

Sign up for MBoC's new content alerts here https://tinyurl.com/3bcsyk3e

Thank you for submitting your manuscript to MBoC. We look forward to receiving your revised paper.

Sincerely,

Eric Baker
Journal Production Manager
MBoC Editorial Office
mbc@ascb.org

Reviewer #1 (Remarks to the Author):

In this manuscript the authors combined super resolution imaging, quantitative smFISH, genetic perturbations, and computational analysis to dissect how RNA concentration, Staufen, and an RNA palindrome synergize to drive oskar mRNA clustering in vivo. The work is important, the manuscript is clearly written and the figures are compelling. Several comments should be addressed to strengthen the manuscript before publication.

Major comments

1. The manuscript argues that Stau doubles the likelihood of osk dimerization and lowers the concentration threshold (Figure 2). Interestingly, Stau binds the stem containing the palindrome even when the palindrome is mutated. Why then does Stau not rescue dimerization of eGFP AA?
2. The increase in osk mRNA levels in stau mutants may be due to decreased osk mRNA decay, as stated by the authors. If feasible, this could be tested.
3. The authors identify 60 mRNAs with GC rich palindromes and highlight eIF3a as a candidate. However, the criteria for "GC rich" and "exposed" palindromes are somewhat arbitrary ({greater than or equal to}50% GC, {greater than or equal to}6 bp pairable positions). RNAfold predictions are known to be unreliable for long UTRs and coding regions. The authors should discuss false positive rates and whether any of these palindromes overlap known structured elements.

Minor Comments

1. Clarify the definition of "cluster size"
2. The authors repeatedly describe their findings in the introduction, which is unconventional and weakens the logical structure of the paper. The introduction should set up background, motivation, and hypotheses, not present results.
3. The authors note that nanos Gal4 was insufficient to rescue egg laying. A brief explanation of why mata Gal4 is stronger or more appropriate would help readers unfamiliar with Drosophila genetics.
4. Several typos (e.g., "paring" instead of "pairing" in Keywords, "ration" instead of ratio).
5. Some sentences are overly long and could be streamlined.

Reviewer #2 (Remarks to the Author):

This manuscript combines quantitative smFISH, super-resolution imaging, and genetic perturbations to investigate how RNA concentration, Staufen, and palindrome-mediated RNA-RNA interactions contribute to oskar mRNA clustering in vivo. The work is technically strong, carefully executed, and addresses an important question in RNA granule biology. In particular, the attempt to quantitatively separate dimerization from higher-order oligomerization represents a potentially important advance beyond prior work on osk granules. The manuscript also extends the field conceptually by proposing that exposed GC-rich palindromes may contribute more broadly to embryonic mRNA organization.

However, several of the central mechanistic conclusions appear stronger than the data directly support. My primary concern is that the perturbation phenotypes presented throughout the manuscript are more consistent with defects in higher-order oligomerization than in the initial dimerization step itself. In both the Staufen-deficient and palindrome-mutant conditions, oligomeric species collapse strongly whereas dimers remain abundant and in some cases become proportionally enriched. Thus, the data could also support a model in which dimers can still form, but their progression into larger assemblies is impaired. The repeated conclusions that Staufen and the palindrome directly "promote dimerization" or "lower the concentration threshold for dimerization" therefore seem overstated, especially because dimerization is inferred indirectly from static fluorescence-intensity distributions rather than measured directly.

A second major concern is the absence of epistasis analysis between Staufen loss and palindrome disruption. Because the central model of the paper is that RNA concentration, Staufen, and palindrome-mediated pairing act synergistically, it is important to determine whether Staufen and the palindrome function in the same pathway, sequentially, or independently. The lack of a combined Staufen/palindrome perturbation substantially limits mechanistic interpretation of the proposed synergy model.

I also have concerns regarding the interpretation of fluorescence-intensity classes as bona fide physical dimers and oligomers.

Although the Gaussian-based analysis is reasonable and carefully performed, diffraction-limited overlap, local crowding, and spot-segmentation effects could contribute to the observed intensity distributions. The manuscript relies heavily on these inferred species assignments to draw mechanistic conclusions about dimerization and oligomerization without orthogonal validation.

In addition, several conceptual claims would benefit from more cautious framing. The manuscript frequently invokes ideas such as "critical concentration thresholds" and cooperative assembly behavior, but these are inferred from static comparisons across genotypes rather than from quantitative concentration titrations or kinetic analyses. Similarly, the claim that the palindrome is the "major determinant" of heterotypic clustering may be stronger than warranted, particularly since the reporter constructs retain numerous additional osk 3′UTR features, including binding sites for Bruno, PTB, and Staufen.

Finally, the extension to other embryonic mRNAs, while interesting, currently feels preliminary. The computational identification of exposed GC-rich palindromes is potentially valuable, but the experimental support for generalization beyond osk remains limited, as the eIF3a analysis is descriptive and does not directly test palindrome dependence.

Overall, this is an interesting and impactful study that provides valuable quantitative observations about osk clustering in vivo. However, the mechanistic interpretation currently exceeds what the data directly demonstrate. Addressing the distinction between dimerization and oligomerization more carefully, and clarifying the relationship between Staufen-dependent and palindrome-dependent assembly pathways, would substantially strengthen the manuscript.

RESPONSE TO ALL REVIEWERS:

We thank the Reviewers for their careful evaluation of our manuscript and for their constructive and thoughtful feedback. We were encouraged that both Reviewers recognized the conceptual advances of our study. Reviewer #1 noted that "The work is important, the manuscript is clearly written and the figures are compelling." Reviewer #2 similarly commented that "The work is technically strong, carefully executed, and addresses an important question in RNA granule biology. In particular, the attempt to quantitatively separate dimerization from higher-order oligomerization represents a potentially important advance beyond prior work on *osk* granules."

At the same time, both Reviewers raised several concerns, primarily regarding the interpretation of critical concentration thresholds, the synergistic mechanisms underlying *oskar* mRNA clustering, and the computational identification of candidate mRNAs that may cluster through exposed palindromic sequences. In the revised manuscript, we have addressed each of these concerns through additional clarification of our interpretations, and revisions to the text (marked in red in the revised manuscript). We believe that these changes have strengthened the manuscript while further supporting our proposed model. Below, we respond to each Reviewer's comments individually.

Reviewer #1:

Major comments:

Q1. The manuscript argues that Stau doubles the likelihood of osk dimerization and lowers the concentration threshold (Figure 2). Interestingly, Stau binds the stem containing the palindrome even when the palindrome is mutated. Why then does Stau not rescue dimerization of eGFP AA?

A1: The Reviewer raises an important question. While we do not yet have a definitive answer, the observation raised by the Reviewer supports a central conclusion of our study: efficient *osk* dimerization and oligomerization require both the GC-rich palindrome and Stau. Neither factor can fully substitute for each other's loss.

In vivo, we observed that Stau alone cannot promote dimerization as effectively as the palindrome itself. Consistent with this interpretation, mutation of the palindrome reduced dimerization approximately twofold more strongly than loss of Stau. Thus, even in the presence of Stau, dimerization of eGFP-AA would not be expected to reach wild-type levels because Stau cannot fully compensate for disruption of the palindrome.

These findings suggest that the palindrome and Stau make distinct, non-redundant contributions to *osk* dimerization that differ in effectiveness. We have discussed these observations in our initially submitted manuscript (see **Lines 587-595**). In the revised manuscript, we have included additional clarifications in the results section: (see **Lines 378-393**). Future studies will be needed to determine the precise mechanistic relationship between these two factors.

Q2. The increase in osk mRNA levels in stau mutants may be due to decreased osk mRNA decay, as stated by the authors. If feasible, this could be tested.

A2: We plan to pursue these experiments in future work. However, we do not believe that these data would be appropriate for inclusion in the present manuscript, as they would detract from its central focus: that optimal *oskar* mRNA clustering is promoted by three factors—RNA concentration, the RNA-binding protein Staufen, and an RNA palindrome.

Establishing a role for Staufen in regulating *osk* mRNA stability would require a substantially expanded set of experiments, including validation that decay occurs through a Staufen-dependent pathway. This would necessitate testing the involvement of Upf1 in *osk* mRNA decay in both the presence and absence of Staufen, as well as assessing that other nonsense-mediated decay factors, including Upf2 and Upf3, do not contribute to this process (excluding the possibility of NMD).

Given the scope of the required mechanistic dissection, we believe that these experiments are better suited for a separate, dedicated study. Although potentially highly informative, they fall outside the scope of the current work. Since a higher *osk* concentration was observed in *stau*$^{-/-}$ mutant embryos we considered it appropriate to propose a mechanistically grounded explanation, supported by published literature, for this unexpected observation, without additional experimental validation in the present study.

Q3. The authors identify 60 mRNAs with GC rich palindromes and highlight eIF3a as a candidate. However, the criteria for "GC rich" and "exposed" palindromes are somewhat arbitrary ({greater than or equal to}50% GC, {greater than or equal to}6 bp pairable positions). RNAfold predictions are known to be unreliable for long UTRs and coding regions. The authors should discuss false positive rates and whether any of these palindromes overlap known structured elements.

A3: In the revised manuscript we further clarified the criteria for 50% GC-rich, exposed, 6-nt long palindrome in our search. We now write that: "While RNA motifs consisting of only a few nucleotides may contribute to intermolecular interactions in vivo, we restricted our analysis to palindromes that were at least six nucleotides long as well as at least 50% GC-rich. In addition to resembling the *osk* palindrome, this threshold was supported by our previous work, which demonstrated using non-denaturing agarose gel electrophoresis that in vitro-transcribed RNAs containing shorter palindromes or palindromes with less than 66% GC content failed to stably dimerize[25].

Using these criteria, we analyzed 11657 non—localizing transcripts (termed "ubiquitous") representing isoforms of 4304 genes (Suppl Table 2)." (see **Lines 459-468**).

Furthermore, we have discussed the limitations of our computational search, discussed the potential over and underestimation of the number of mRNAs with exposed palindromes and discussed potential overlap with previously mapped and predicted RNA structures (see **Lines 495-499; 522-529; 641-661**).

Minor Comments

Q1: Clarify the definition of "cluster size"

A1: We have now more clearly defined the term "*oskar* cluster size" (see **Lines 172-173**).

Q2: The authors repeatedly describe their findings in the introduction, which is unconventional and weakens the logical structure of the paper. The introduction should set up background, motivation, and hypotheses, not present results.

A2: In the revised manuscript, we removed the majority of the descriptions of the results from the introduction as the Reviewer suggested. Now we simply conclude it by stating: "Here, we demonstrate that RNA concentration, Staufen, and the RNA palindrome together promote *osk* mRNA clustering. Our work suggests that this multi-layered requirement likely increases the stringency of clustering, making it a tightly regulated and context-dependent process in vivo." (see **Lines 157-160**).

Q3: The authors note that nanos Gal4 was insufficient to rescue egg laying. A brief explanation of why mata Gal4 is stronger or more appropriate would help readers unfamiliar with Drosophila genetics.

A3: We have provided additional explanations about the gene expression activities of both the UAS-Gal4/*nanos promoter* system and the UAS-Gal4/*matα* promoter system (see **Lines 342-347**).

Q4: Several typos (e.g., "paring" instead of "pairing" in Keywords, "ration" instead of ratio).

A4: We have fixed these typos in the revised manuscript (edited in red) – thank you for pointing them out to us.

5. Some sentences are overly long and could be streamlined.

Reviewer #2:

Q1: My primary concern is that the perturbation phenotypes presented throughout the manuscript are more consistent with defects in higher-order oligomerization than in the initial dimerization step itself. In both the Staufen-deficient and palindrome-mutant conditions, oligomeric species collapse strongly whereas dimers remain abundant and in some cases become proportionally enriched. Thus, the data could also support a model in which dimers can still form, but their progression into larger assemblies is impaired. The repeated conclusions that Staufen and the palindrome directly "promote dimerization" or "lower the concentration threshold for dimerization" therefore seem overstated, especially because dimerization is inferred indirectly from static fluorescence-intensity distributions rather than measured directly.

A1: We agree with the Reviewer's interpretation of our data and the model they propose. This is, in fact, the model we intended to convey in the original manuscript, although we now realize that it was not presented clearly enough.

Our data indicate that both dimerization and oligomerization are affected by Stau depletion and palindrome mutations, with oligomerization being considerably more sensitive to these perturbations. Importantly, while the effects on dimerization are more modest than those on oligomerization, they are nonetheless evident. Specifically, we observe an accumulation of *osk* and eGFP monomers relative to their respective WT controls. If dimerization was unaffected and only the transition from dimers to higher-order oligomers was impaired, one would expect dimers to accumulate substantially. Instead, we observe a pronounced decrease in oligomers accompanied by an increase in monomers, indicating that both dimerization and oligomerization are disrupted. Since oligomerization was more strongly impacted, our observations further suggest that oligomerization requires additional intermolecular interactions beyond those necessary for dimerization, making it more sensitive to perturbations in Stau function, palindrome-mediated interactions, and mRNA concentration.

In the revised manuscript, we have clarified this model by stating: "Given this accumulation of *osk* monomers, which suggested an impaired dimer formation, our data suggest that dsRBP Stau promoted *osk* dimerization in vivo by approximately doubling its probability.

Collectively, our data support a model in which dimers can still form in the absence of Stau, although at reduced frequencies compared to WT embryos, whereas their progression into larger *osk* mRNA clusters is much more severely impaired. These findings indicate that *osk* clustering is governed by both *osk* mRNA concentration and dsRBP Stau, with the higher-order oligomer formation being more acutely sensitive to perturbations in these factors than dimerization. This differential sensitivity suggests that dimerization and oligomerization rely on partially distinct combinations of intermolecular interactions, with oligomerization requiring additional interactions beyond those necessary for dimer formation." (see **Lines 311-322**).

The same revision applies for our studies of the *osk* palindrome. We now write that: "These results revealed that *eGFP-AA* mRNA with a disrupted palindrome still dimerized, albeit nearly fourfold less efficiently that *eGFP-WT* containing a WT palindrome. Thus, the palindrome is required for efficient *osk* dimerization and subsequent oligomerization. Notably, although mutation of the palindrome reduced dimerization, higher-order oligomerization was affected much more severely, suggesting that oligomerization requires additional intermolecular interactions than those sufficient for dimerization.

Furthermore, mutation of the palindrome reduced dimerization approximately twofold more strongly than loss of Stau (Fig. 2G), indicating that Stau alone cannot promote dimerization as effectively as the palindrome itself. Consistent with this interpretation, Stau was unable to fully compensate for the disruption of the palindrome. Thus, while both factors contribute to *osk* dimerization, the palindrome appears to play the more prominent role.

Taken together, these findings suggest that the palindrome and Stau make distinct, non-redundant contributions to *osk* dimerization, with efficient oligomerization requiring the contribution of both factors." (see **Lines 378-393**).

We have also removed all mention of Stau or the palindrome affecting the concentration threshold for dimerization as the concentration-dependent studies of this process have not been conducted (also **please see Q4 and A4 below**).

Q2: I also have concerns regarding the interpretation of fluorescence-intensity classes as bona fide physical dimers and oligomers. Although the Gaussian-based analysis is reasonable and carefully performed, diffraction-limited overlap, local crowding, and spot-segmentation effects could contribute to the observed intensity distributions. The manuscript relies heavily on these inferred species assignments to draw mechanistic conclusions about dimerization and oligomerization without orthogonal validation.

A2: We agree with the Reviewer that fluorescence imaging alone cannot definitively establish physical interactions between individual *osk* mRNAs. However, our interpretation is supported by a substantial body of prior work demonstrating that *osk* mRNAs organize into clusters through specific intermolecular interactions.

Specifically, previous studies showed that localizing *osk* mRNAs hitchhike on one another to enhance posterior enrichment (1), and that this process is driven by a GC-rich palindromic sequence located within the stemloop structure located in its 3′UTR (1,2). Importantly, this palindrome mediates intermolecular base pairing both in vitro and in vivo (1,3). Furthermore, our previous work using stochastic optical reconstruction microscopy (STORM) demonstrated that the palindrome engages in persistent intermolecular interactions within *osk* mRNA clusters in vivo (4). These experiments also revealed that, despite containing multiple *osk* mRNAs, the physical dimensions of the clusters do not exceed ~100 nm, which is below the resolution limit of the structured illumination microscopy approach used in the current study. Therefore, although our imaging approach cannot directly resolve intermolecular contacts between individual mRNAs, previous studies have established that *osk* molecules within these diffraction-limited clusters physically interact through sequence-specific intermolecular base pairing. Consequently, our interpretation of the clustering data is consistent with the known organization of *osk* in vivo.

In addition, we performed several control experiments demonstrating that the observed clustering behavior is a specific property of *osk* and not an imaging artifact. First, regression analysis showed that *osk* mRNA abundance can be accurately quantified regardless of clustering state, allowing identification of different cluster populations, including monomers (Suppl. Fig. 1E). Second, clustering was not observed for mRNAs expressed at higher concentrations than *osk* (Fig. 1I), indicating that *osk* clustering is not a consequence of crowding coupled with insufficient image resolution. Third, all *osk* cluster populations exhibited similar diffraction-limited dimensions (Suppl. Fig. 1F–H), consistent with multiple mRNAs occupying the same sub-resolution volume characterized by STORM. Finally, *ccr4* mRNA, which displayed a similar density of fluorescent

spots as *osk*, did not exhibit clustering behavior (Fig. 1E–H), further demonstrating that clustering is not an artifact of image acquisition or analysis.

Finally, endogenous *osk* and the eGFP-WT transgene co-localized and formed mixed oligomers (Fig. 3). The simplest explanation for this behavior is that the two RNAs engage in the same intermolecular interactions that drive endogenous *osk* clustering (see **Lines 402-404**).

Taken together, our findings are consistent with extensive previous evidence demonstrating that intermolecular interactions drive *osk* mRNAs clustering. We therefore believe that the imaging approach used here is appropriate for investigating the mechanisms governing *osk* dimerization and oligomerization. We have discussed these published observations in the introduction (see **Lines 121-135**) and the result section (see **Lines 325-332**).

1: Jambor, H., Brunel, C. & Ephrussi, A. Dimerization of oskar 3' UTRs promotes hitchhiking for RNA localization in the Drosophila oocyte. *RNA* **17**, 2049-2057, doi:10.1261/rna.2686411 (2011).

2: Jambor, H., Mueller, S., Bullock, S. L. & Ephrussi, A. A stem-loop structure directs oskar mRNA to microtubule minus ends. *RNA* **20**, 429-439, doi:10.1261/rna.041566.113 (2014).

3: Bose, M., Rankovic, B., Mahamid, J. & Ephrussi, A. An architectural role of specific RNA-RNA interactions in oskar granules. *Nat Cell Biol* **26**, 1934-1942, doi:10.1038/s41556-024-01519-3 (2024).

4: Trcek, T. *et al.* Sequence-Independent Self-Assembly of Germ Granule mRNAs into Homotypic Clusters. *Mol Cell* **78**, 941-950 e912, doi:10.1016/j.molcel.2020.05.008 (2020).

Q3: A second major concern is the absence of epistasis analysis between Staufen loss and palindrome disruption. Because the central model of the paper is that RNA concentration, Staufen, and palindrome-mediated pairing act synergistically, it is important to determine whether Staufen and the palindrome function in the same pathway, sequentially, or independently. The lack of a combined Staufen/palindrome perturbation substantially limits mechanistic interpretation of the proposed synergy model.

A3: It was not our intention to suggest that the three factors cooperate in driving *oskar* mRNA clustering. We simply wanted to state that the three factors are all required for this process. Therefore, we have omitted the word "synergy" from our manuscript as well as from the title, which now states that "In vivo clustering of *oskar* mRNA is driven by RNA concentration, RNA binding proteins, and an RNA palindrome".

Q4: The manuscript frequently invokes ideas such as "critical concentration thresholds" and cooperative assembly behavior, but these are inferred from static comparisons across genotypes rather than from quantitative concentration titrations or kinetic analyses.

A4: The Reviewer is correct in stating that we have not determined the concentration threshold required for *oskar* mRNA dimerization nor the effect of Staufen and the palindrome in changing this threshold. We agree with the Reviewer that quantitative titration measurements or kinetic analysis would be required to obtain such measurements. We have removed reference to the critical concentration thresholds in the revised manuscript.

Q5: Similarly, the claim that the palindrome is the "major determinant" of heterotypic clustering may be stronger than warranted, particularly since the reporter constructs retain numerous additional osk 3′UTR features, including binding sites for Bruno, PTB, and Staufen.

A5: We agree with the Reviewer that the term "major determinant" of heterotypic clustering is misleading. In the revised manuscript we now state that "Notably, insertion of the *osk* palindrome into a reporter mRNA markedly increased its association with the endogenous *osk*, identifying the palindrome as the potent driver of heterotypic mRNA clustering while further supporting its role in mediating intermolecular base pairing." (see **Lines 41, 395, 402-404, 436, 446, 606, 609, 1212, 1252**).

Q6: Finally, the extension to other embryonic mRNAs, while interesting, currently feels preliminary. The computational identification of exposed GC-rich palindromes is potentially valuable, but the experimental support for generalization beyond osk remains limited, as the eIF3a analysis is descriptive and does not directly test palindrome dependence.

A6: We agree with the Reviewer that extending our findings to other mRNAs is preliminary and that further experimental validations are necessary to establish these candidate mRNAs as bona fide mRNAs whose clustering is driven by palindrome-mediated intermolecular base pairing. We did not intend to present these findings as definitive. Accordingly, we were careful to state that these mRNAs are candidate mRNAs whose clustering may be driven by palindromes (the underlined words point to the original wording). To further emphasize the preliminary nature of these findings we revised the Abstract to state: "Finally, computational analyses identified a subset of candidate mRNAs in the early embryo that are predicted to harbor *oskar*-like palindromes. Among these, *eIF3a* mRNA emerged as a potential candidate whose clustering may likewise be driven by intermolecular base pairing. These preliminary observations raise the possibility that mRNA clustering driven by palindrome-mediated intermolecular base pairing may be more widespread than previously appreciated and may represent an important mechanism for controlling mRNA spatial organization during *Drosophila* development."

Furthermore, we have discussed the limitations of our computational search, discussed the potential over and underestimation of the number of mRNAs with exposed palindromes and

discussed potential overlap with previously mapped and predicted RNA structures (see **Lines 495-499, 522-529, 641-661**).

RE: Manuscript #E26-04-0176R
TITLE: "In vivo clustering of oskar mRNA is driven by RNA concentration, RNA binding proteins, and an RNA palindrome"

Dear Dr. Trcek:

Congratulations on this excellent work, and thank you for your thorough and thoughtful responses to the reviewers. I am pleased to inform you that your manuscript has been provisionally accepted for publication in Molecular Biology of the Cell (MBoC) pending completion of the minor revisions listed below.

To finalize acceptance, please address the following items:

Please address the minor points of concern regarding the wording of your conclusions that are still of concern to Reviewer 2. This can be done by further tempering the conclusions drawn.

Once these revisions are submitted and verified, we will proceed with formal acceptance.

Thank you again for your contribution to MBoC.

Sincerely,
Anita Corbett
Monitoring Editor
Molecular Biology of the Cell

Dear Dr. Trcek,

The review of your manuscript, referenced above, is now complete. The Monitoring Editor has decided that your manuscript requires minor revisions before it can be published in Molecular Biology of the Cell, as described in the Monitoring Editor's decision letter above and the reviewer comments (if any) below.

A reminder: Please do not contact the Monitoring Editor directly regarding your manuscript. If you have any questions regarding the review process or the decision, please contact the MBoC Editorial Office (mboc@ascb.org).

When submitting your revision include an authors' response letter that details, point-by-point, how the Monitoring Editor's and reviewers' comments have been addressed. (The file type for this letter must be "response letter"; do not include your response to the Monitoring Editor and reviewers in a "cover letter.") Please bear in mind that your response letter will be published with your paper if it is accepted, unless you have opted out of publishing the review history.

Authors are allowed 180 days to submit a revision. If this time period is inadequate, please contact us immediately at mboc@ascb.org.

In preparing your revised manuscript, please follow the instructions in the Information for Authors (www.molbiolcell.org/info-for-authors). In particular, to prepare for the possible acceptance of your revised manuscript, submit final, publication-quality figures with your revision as described.

To submit the response letter, revised version, and figures, please use this link (please enable cookies, or cut and paste URL):
Link Not Available

MBoC offers the option to publish your work with immediate open access. Open access can increase the discoverability and usability of your research. If you would like to publish your paper with immediate open access but did not select this option during initial submission, please contact us at mbc@ascb.org.

Authors of Articles and Brief Communications whose manuscripts have returned for minor revision ("provisional acceptance") are encouraged to create a short video abstract to accompany their article when it is published. These video abstracts, known as Science Sketches, are up to 2 minutes long and will be published on YouTube and then embedded in the article abstract. Science Sketch Editors on the MBoC Editorial Board will guide you as you prepare your video. Information about how to prepare and submit a video abstract is available at www.molbiolcell.org/science-sketches. Please contact mboc@ascb.org if you are interested in creating a Science Sketch.

Thank you for submitting your manuscript to MBoC. Please do not hesitate to contact this office if you have any questions.

Sincerely,

Eric Baker
Journal Production Manager
MBoC Editorial Office
mbc@ascb.org

Reviewer #1 (Remarks to the Author):

The authors have done a great job addressing my comments.

Reviewer #2 (Remarks to the Author):

The authors have engaged constructively with all of the reviewers' concerns and have made useful textual revisions. In particular, removing the "critical concentration threshold" language, softening the heterotypic clustering claim, and clarifying that the eIF3a analysis is preliminary are appropriate and improve the manuscript. The rebuttal is generally clear and professional.

However, the response is primarily based on reframing rather than new data, and the central mechanistic concerns remain only partially resolved. The revised interpretation still infers effects on dimerization from static fluorescence-intensity distributions, while the strongest phenotypic effect remains a loss of higher-order oligomers. The lack of a combined Staufen/palindrome perturbation continues to limit the mechanistic conclusion that these factors act as distinct, nonredundant contributors. Removing the word "synergy" helps, but the remaining claim that all three factors are "required" may still be too strong as it stands.

RESPONSE TO ALL REVIEWERS:

We thank the Reviewers for their careful evaluation of our manuscript and for their feedback. In the revised manuscript, we have addressed the remaining concerns raised by the Reviewer #2 (marked in red in the revised manuscript). These revisions are as follows:

Reviewer #1:

Q1: The authors have done a great job addressing my comments.

A1: Thank you.

Reviewer #2:

Q1: The authors have engaged constructively with all of the reviewers' concerns and have made useful textual revisions. In particular, removing the "critical concentration threshold" language, softening the heterotypic clustering claim, and clarifying that the eIF3a analysis is preliminary are appropriate and improve the manuscript. The rebuttal is generally clear and professional.

However, the response is primarily based on reframing rather than new data, and the central mechanistic concerns remain only partially resolved. The revised interpretation still infers effects on dimerization from static fluorescence-intensity distributions, while the strongest phenotypic effect remains a loss of higher-order oligomers. The lack of a combined Staufen/palindrome perturbation continues to limit the mechanistic conclusion that these factors act as distinct, nonredundant contributors. Removing the word "synergy" helps, but the remaining claim that all three factors are "required" may still be too strong as it stands.

A1: To improve the clarity of our model, we have made the following modifications to our manuscript, as suggested by the Reviewer (see edits made in red):

Lines 857-860: Notably, our interpretation of changes in *osk* mRNA clustering relies on fluorescence-intensity distributions. This approach provides greater phenotypic resolution when assessing differences between monomers and higher-order mRNA clusters, as they exhibit the largest difference in integrated fluorescence intensity.

Figure 4, Panel F – Model: We have drawn a single arrow from the three factors—RNA concentration, the osk palindrome, and the Staufen RBP—to emphasize that these factors drive efficient osk mRNA clustering and to avoid the potential interpretation that Staufen and the palindrome act independently. We have also clarified this point in the manuscript by writing:

Line 56: Although these factors drive efficient *oskar* clustering, they contribute distinctly to different stages of cluster assembly. We replaced the words “are required” with the word “drive”.

Lines 157-159: Here, we demonstrate that RNA concentration, Staufen, and the RNA palindrome together promote *osk* mRNA clustering, with *osk* oligomerization being the most strongly affected by the perturbations to these three factors.

Line 538: … stringent and are driven by high RNA concentration, and Stau and the RNA palindrome. We replaced the words "require the combined action" with the "are driven by".

Lines 545-546: …palindrome alone is insufficient for efficient HIV-1 RNA dimerization, and additional factors participate. We replaced the words "are required" with the "participate".

We also added the following two clarifications:

Lines 553-556: Notably, Stau and the palindrome may function independently to promote *osk* clustering, or they may act interdependently. Future analysis of the combined Stau/palindrome mutations are required to distinguish between these mechanisms and clarify their respective contributions to *osk* clustering.

Lines 1255-1257: Importantly, Stau and the palindrome may function as distinct, nonredundant contributors to *osk* mRNA dimerization and oligomerization, or may have interdependent activities.

RE: Manuscript #E26-04-0176RR
TITLE: "In vivo clustering of oskar mRNA is driven by RNA concentration, RNA binding proteins, and an RNA palindrome"

Dear Dr. Trcek:

I am pleased to accept your manuscript for publication in Molecular Biology of the Cell.

Thank you for addressing the remaining reviewer comments by tempering some of the conclusions and the wording.

Sincerely,
Anita Corbett
Monitoring Editor
Molecular Biology of the Cell

Dear Dr. Trcek:

Congratulations on the acceptance of your manuscript! Thank you for publishing your work in Molecular Biology of the Cell (MBoC).

Within 10 days, an unedited PDF of your manuscript will be published on MBoC in Press, an early-release journal version. The date your manuscript appears on this site is the official publication date.

Your copyedited and typeset manuscript will be scheduled for publication in the next available issue of MBoC. Once your paper is ready for review, our production team will notify you. In the summer of 2024, our production provider will introduce an online proofing solution, which will streamline the process of checking and validating author changes. This will result in improved typesetting quality, enhanced digital content, and a faster overall process.

We invite you to submit images related to your accepted manuscript to be considered for the journal cover.
Cover images must contain color, TIF Format
Size image at 7.4" wide x 5.85" deep
RGB and CMYK color (please submit two separate files, one in each format)
300 dpi resolution

Please also send a 1-paragraph description of the image that a general audience could understand. Include with the caption the name and institution of the individual(s) the image should be attributed to. Please provide this description in a Word file. You can send the file via email or by your favorite file transfer service to mboc@ascb.org.

Authors of Articles and Brief Communications are also encouraged to create a short video abstract to accompany their article when it is published. These video abstracts, known as Science Sketches, are up to 2 minutes long and will be published on YouTube and then embedded in the article abstract. Science Sketch Editors on the MBoC Editorial Board will guide you as you prepare your video. Information about how to prepare and submit a video abstract is available at www.molbiolcell.org/science-sketches. Please contact mboc@ascb.org if you are interested in creating a Science Sketch.

We look forward to publishing your paper in MBoC.

Sincerely,

Eric Baker
Journal Production Manager
MBoC Editorial Office
mbc@ascb.org
